# A Kinetic Platform to Determine the Fate of Nitric Oxide in *Escherichia coli*

**DOI:** 10.1371/journal.pcbi.1003049

**Published:** 2013-05-02

**Authors:** Jonathan L. Robinson, Mark P. Brynildsen

**Affiliations:** Department of Chemical and Biological Engineering, Princeton University, Princeton, New Jersey, United States of America; University of Illinois at Urbana-Champaign, United States of America

## Abstract

Nitric oxide (NO•) is generated by the innate immune response to neutralize pathogens. NO• and its autoxidation products have an extensive biochemical reaction network that includes reactions with iron-sulfur clusters, DNA, and thiols. The fate of NO• inside a pathogen depends on a kinetic competition among its many targets, and is of critical importance to infection outcomes. Due to the complexity of the NO• biochemical network, where many intermediates are short-lived and at extremely low concentrations, several species can be measured, but stable products are non-unique, and damaged biomolecules are continually repaired or regenerated, kinetic models are required to understand and predict the outcome of NO• treatment. Here, we have constructed a comprehensive kinetic model that encompasses the broad reactivity of NO• in *Escherichia coli*. The incorporation of spontaneous and enzymatic reactions, as well as damage and repair of biomolecules, allowed for a detailed analysis of how NO• distributes in *E. coli* cultures. The model was informed with experimental measurements of NO• dynamics, and used to identify control parameters of the NO• distribution. Simulations predicted that NO• dioxygenase (Hmp) functions as a dominant NO• consumption pathway at O_2_ concentrations as low as 35 µM (microaerobic), and interestingly, loses utility as the NO• delivery rate increases. We confirmed these predictions experimentally by measuring NO• dynamics in wild-type and mutant cultures at different NO• delivery rates and O_2_ concentrations. These data suggest that the kinetics of NO• metabolism must be considered when assessing the importance of cellular components to NO• tolerance, and that models such as the one described here are necessary to rigorously investigate NO• stress in microbes. This model provides a platform to identify novel strategies to potentiate the effects of NO•, and will serve as a template from which analogous models can be generated for other organisms.

## Author Summary

Nitric oxide (NO•) is a highly reactive metabolite used by immune cells to combat pathogens. Since the biological effects of NO• are governed by its broad reactivity, it is desirable to determine how NO• distributes among its many targets inside a cell. A quantitative understanding of this distribution and how it is controlled will facilitate the development of novel NO•-potentiating therapeutics. Here, we have constructed and experimentally validated a comprehensive kinetic model of NO• biochemistry within *Escherichia coli* that includes NO• autoxidation, respiratory inhibition, enzymatic detoxification, and damage and repair of biomolecules. Using this model, we investigated the control of NO• dynamics in *E. coli* cultures, and found that the primary aerobic detoxification system, NO• dioxygenase (Hmp), functions as a dominant NO• consumption pathway under microaerobic conditions (35 µM O_2_), and loses utility as the NO• delivery rate increases. We confirmed these predictions experimentally, thereby demonstrating the predictive power of the model. This model will serve as a quantitative platform to study nitrosative stress, provide a template from which models for other organisms can be generated, and facilitate the development of antimicrobials that synergize with host-derived NO•.

## Introduction

**Figure 1:**
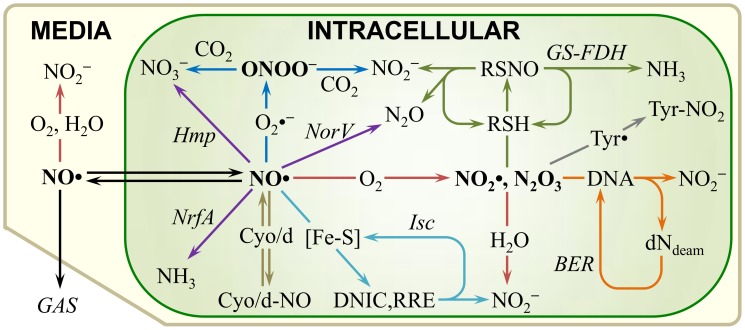
Simplified diagram of the NO• biochemical reaction network in an *E. coli* culture. The intracellular and extracellular (media) compartments are represented by the green and tan shaded regions, respectively. The lower-left corner represents the gas phase in direct contact with the liquid media. Species in bold text represent NO• and its reactive oxidized forms (NO_2_•, N_2_O_3_, and ONOO^−^). Italic text indicates the enzyme or group of enzymes responsible for the associated reaction/pathway. Red reaction arrows represent NO• autoxidation; purple, enzymatic detoxification; blue, ONOO^−^ formation and degradation; tan, cytochrome inhibition; teal, [Fe-S] nitrosylation and repair; green, thiol nitrosation and denitrosation; gray, tyrosine nitration; orange, DNA deamination and repair.

NO• is an uncharged, highly diffusible, membrane-permeable metabolite, generated by mammalian NO• synthases (NOS) for use in signaling and defense [1], [2]. The diversity of functions performed by NO•, from pathogen detoxification to vasodilation, reflect its broad reactivity. NO• directly reacts with iron-sulfur ([Fe-S]) clusters, superoxide (O_2_•^−^), and O_2_, whereas its oxidized forms (NO_2_•, N_2_O_3_, and N_2_O_4_) damage thiols, tyrosine residues, and DNA bases [2]–[5]. Such widespread activity has made the biological effects of NO• difficult to predict [2]. For instance, if 1,000 NO• molecules entered a cell, what would become of them? How many would disrupt an [Fe-S] cluster to form a protein-bound dinitrosyl-iron complex (DNIC)? How many would autoxidize to form nitrogen dioxide (NO_2_•) and then react with another NO• to form nitrous anhydride (N_2_O_3_)? How many N_2_O_3_ would deaminate DNA bases? These questions are representative of one unifying, fundamental question of NO• metabolism: how does NO• distribute within a cell? The answer to this question lies in understanding the kinetic competition of NO• with its many intracellular targets. However, the NO• biochemical network is complex (Figure 1), contains numerous short-lived intermediates at low concentrations [6], converges to only a few stable end-products [4], and involves various damaged biomolecules that are continually digested or repaired [7]. Such complexity has necessitated the use of computational models to both interpret and predict the outcome of NO• treatment [4].

A number of kinetic models have been developed to simulate NO• chemistry in biological contexts [3], [4], [6]–[17]. Many of these models have focused on mammalian systems due to the importance of NO• in human physiology. Nalwaya and Deen [9] calculated steady-state concentration profiles of NO•, CO_2_, O_2_•^−^, and peroxynitrite (ONOO^−^) in idealized mammalian cell cultures using a reaction–diffusion model, and explored the effect of varying the rates and locations (extracellular, mitochondrial, or cytosolic) of their generation. Their results suggested negligible spatial variation in species concentrations, and identified conditions under which the different cellular compartments serve as dominant sources or sinks. However, their model did not include the reactions of numerous intracellular metabolites that either directly react with NO•, or its autoxidation products (NO_2_•, N_2_O_3_, and N_2_O_4_). Lancaster [3] constructed a non-diffusive, but more extensive kinetic model to encompass the complex reaction network of NO• and its autoxidation products with glutathione (GSH) and tyrosine in mammalian systems. This model allowed for predictions regarding the relative importance of the various NO•-consuming pathways under inflammatory and non-inflammatory regimes, and highlighted the dominance of oxidative reactions. Lim *et al.* [4] built upon the work of Lancaster [3] by incorporating additional antioxidants, as well as a separate membrane compartment to account for partitioning of certain species in the lipid-phase. Their model was developed to be representative of inflamed tissue *in vivo* and used to estimate steady-state intracellular concentrations of different reactive nitrogen species (RNS), in addition to identifying their major sources and sinks in the cytosol and membrane compartments of mammalian cells. Interestingly, none of these models considered the interaction of NO• and its autoxidation products with [Fe-S] clusters, cytochromes, or DNA, and their treatment of the relevant enzymatic processes was limited to NO• dioxygenase and superoxide dismutase. Recently, Tórtora *et al.* [7] measured rates of ROS- and RNS-induced damage to the mitochondrial aconitase [4Fe-4S] cluster, and incorporated the reactions into a kinetic model of aconitase inactivation in the presence of O_2_•^−^ and NO•. Since their focus was specifically on the inactivation of aconitase, they did not consider much of the extensive reaction network of NO•, O_2_•^−^, and their products. Bagci *et al.* [14] merged a mitochondrial apoptotic network [18] with a kinetic model of NO• chemistry [10] and extended treatment to include formation of N_2_O_3_, NO_2_• and ONOO^−^, as well as their interactions with GSH, non-heme iron, and mitochondrial cytochrome *c*. However, their attention was primarily on the dynamics of the apoptotic response, and many RNS-related reactions and biological species that were not directly involved in apoptosis were omitted. Though previous models provide a firm foundation for modeling NO• in biological systems, none are sufficiently comprehensive to quantify the distribution of NO• among its many intracellular consumption pathways.

Here, we describe the construction, experimental validation, and utility of a comprehensive model of NO• metabolism in *Escherichia coli*. This model includes NO• autoxidation, enzymatic detoxification, [Fe-S] damage, thiol and tyrosine nitrosation, DNA base deamination, tyrosine nitration, and the repair steps responsible for regeneration of RNS targets. A model of NO• stress with this level of detail has not been previously recognized for any organism. Using this model, we quantitatively explored the distribution of NO• consumption in *E. coli*, and predicted that the utility of the major aerobic NO• detoxification system (Hmp) depends on the NO• delivery rate and extends to environments with O_2_ concentrations as low as 35 µM (microaerobic). We went on to experimentally confirm these predictions, thereby demonstrating the utility of this model to the study of NO• metabolism. This computational model will serve as a platform to quantitatively interrogate the kinetic competition of NO• with its many targets in *E. coli*, and assess the influence of various parameters on its distribution.

## Results

### Kinetic model of NO• metabolism in *E. coli*

Upon diffusing into *E. coli*, NO• may be consumed directly through enzymatic detoxification (Hmp, NorV, NrfA), or reactions with [Fe-S] clusters, O_2_•^−^, or O_2_ (Figure 1, Figures S1, S2). Several resulting nitrosative species, including NO_2_• and N_2_O_3_, can further react to deaminate DNA bases, nitrosate protein and low molecular weight thiols, and nitrate tyrosine residues. To quantify how NO• distributes within a cell, we have constructed a comprehensive kinetic model of the NO• biochemical reaction network in *E. coli*, where autoxidation, detoxification (Hmp, NorV, NrfA), [2Fe-2S] and [4Fe-4S] damage and repair, thiol nitrosation and denitrosation, DNA base deamination and repair, enzyme expression and degradation, tyrosine nitration, and reversible cytochrome inhibition are included (Figure 1). The model consists of 179 reactions, 132 chemical and biochemical species, and 163 kinetic parameters (Tables S1, S2, S3, Text S1). Of the kinetic parameters, 24 have values that are uncertain, either due to variability or unavailability in literature (Table S4). An overview describing the construction of the model is presented in the Materials and methods section, whereas a more detailed description has been presented in Text S1. Due to its scope and completeness, the model is suited to predict the distribution of NO• consumption among the available pathways in *E. coli*. For example, the fraction of NO• detoxified by Hmp, the amount of NO_2_•, N_2_O_3_, and ONOO^−^ formed, the quantity of [Fe-S] clusters and DNA bases damaged and repaired, the extent and duration of cytochrome inhibition, and amount of thiols nitrosated can all be calculated from model simulations. Further, the model allows parameter variation (for example, enzyme mutation/deletion) and quantification of the impact these alterations have on NO• metabolism. To substantiate the utility of the model, we first validated that the model could reproduce experimentally-measured NO• dynamics and make accurate predictions of experimental outcomes.

### Experimental validation

**Figure 2:**
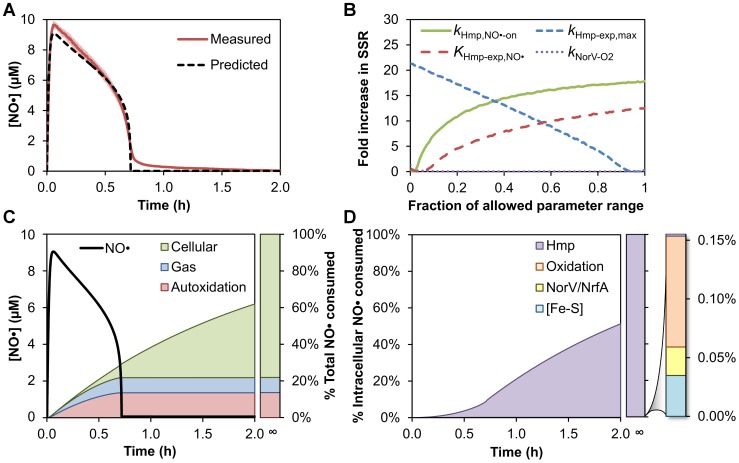
Dynamics of NO• in aerobic wild-type *E. coli* cultures. **(A)** NO• concentration following delivery of 0.5 mM DPTA to a culture of aerobic, exponential-phase, wild-type *E. coli* at an OD_600_ of 0.05 was measured experimentally (solid red line) and predicted by the model (dashed black line). Error bars (light red) represent the standard error of the mean for 3 independent experiments. **(B)** Fold increase in SSR between the experimentally measured and predicted NO• concentration as a function of parameter value for uncertain parameters that significantly affected the SSR upon variation. The remaining 35 parameters exhibited a negligible increase in SSR when varied. **(C)** Simulated NO• concentration profile (black line) and corresponding cumulative distribution of total NO• consumption following the addition of 0.5 mM DPTA to wild-type *E. coli*. The stacked, shaded regions represent the predicted cumulative fraction of NO• consumed by each pathway, where the bar to the right of the plot represents the final distribution of NO• consumption at the limit *t*→∞. “Cellular” refers to NO• consumed by any intracellular pathway, “Gas” is loss of NO• to the gas phase, and “Autoxidation” is the reaction of NO• with O_2_ in the growth media. **(D)** Predicted cumulative distribution of intracellular NO• consumption in wild-type culture following addition of 0.5 mM DPTA. Additional bar at far right shows the contribution of other pathways that are not visible on the full 0–100% scale. “Hmp” is detoxification of NO• by Hmp, “Oxidation” is NO• consumed through reaction with O_2_ or O_2_•^−^, “NorV/NrfA” is the reduction of NO• by NorV or NrfA, and “[Fe-S]” is NO• consumed by the nitrosylation of iron-sulfur clusters.

We sought to validate that the model could capture NO• dynamics in *E. coli* cultures. Since extracellular NO• loss, including autoxidation and gas phase transport, was non-negligible, we bridged the intracellular model to the experimental system by adding an extracellular (growth media) compartment that accounted for autoxidation and gas-phase transport (Materials and methods). Kinetic parameters specific to the extracellular compartment (NO• delivery rate, NO• and O_2_ gas phase mass transfer coefficients, and NO• autoxidation rate) were determined from experimental NO• and O_2_ measurements in the absence of cells (Materials and methods, Figure S3, Text S1). In the experimental system, exponential-phase wild-type *E. coli* were treated with 0.5 mM dipropylenetriamine (DPTA) NONOate, and the concentration of NO• in the culture was monitored over time (Materials and methods). The NO• concentration peaked rapidly to 9.7 µM following delivery of DPTA, and decreased at a steady rate for ∼0.6 hours, after which the concentration dropped quickly to submicromolar levels (Figure 2A). Using a nonlinear least squares optimization algorithm, 39 uncertain parameters (24 kinetic constants and 15 species concentrations) from the model were optimized to capture the experimentally-measured NO• concentration profile (Materials and methods, Table S4). Uncertain parameters were defined as those that were absent from literature, or those whose literature values had a high degree of variability. All other parameters were either set to their literature values, or measured independently in our experimental apparatus (Tables S1, S2, S3, Text S1). Given that the optimization algorithm does not guarantee identification of the globally optimal solution, 100 independent sets of random initial parameter values were used (Materials and methods). The optimized parameter set yielding the lowest sum of squared residuals (SSR) between the simulated and experimental [NO•] curve is presented in Figure 2A, and demonstrates the model's ability to capture NO• dynamics in a wild-type *E. coli* culture. For comparison, we took the three most comparable NO• models [3], [4], [9], adapted them to our experimental conditions, and performed an analogous nonlinear least squares optimization in an attempt to capture the NO• dynamics of wild-type *E. coli* cultures (Materials and methods). As depicted in Figure S4, none of the alternative models could accurately simulate *E. coli* NO• dynamics. Quantitatively, the SSRs between the experimental data and the [NO•] curves predicted by the adapted, alternative models of Lim *et al.* [4], Lancaster [3], and Nalwaya and Deen [9] were, respectively, 200-, 200-, and 70-fold greater than that of the model presented here. These data convincingly demonstrate that the model presented here far exceeds current state-of-the-art kinetic models for simulation of microbial NO• metabolism.

With an ability to simulate NO• dynamics confirmed, we sought to identify which of the 39 parameters adjusted by the nonlinear optimization procedure were informed by the process, and which had a negligible influence under these conditions. We varied each parameter individually and calculated the corresponding increase in SSR, keeping all other parameters at their optimized values (Figure 2B). The analysis revealed that the Hmp NO• binding (*k*_Hmp,NO•-on_), and Hmp expression (*k*_Hmp-exp,max_ and *K*_Hmp-exp,NO•_) parameters were the most influential, whereas the oxidation of NorV (*k*_NorV-O2_) was of minor significance, but exhibited a greater effect than the remaining parameters, which were all negligible (less than a 5% increase in SSR) (Figure S5). This prompted us to identify the minimum biochemical reaction network necessary to simulate NO• dynamics in aerobic, wild-type *E. coli* cultures (Materials and methods). As depicted in Table S5, the model presented here can be simplified to include 17 reactions, 18 chemical and biochemical species, and 14 kinetic parameters without exceeding an overall 5% increase in SSR. While this simplified model can capture the NO• dynamics presented in Figure 2A, we note that it is not suitable for the calculation of additional NO• outcomes, such as the degree of [Fe-S] cluster damage or cytochrome inhibition, and it is not generally translatable to other experimental conditions, such as anaerobic environments. The comprehensive model, on the other hand, can perform such calculations and be applied under many more experimental conditions.

The importance of parameters governing Hmp detoxification activity suggested a dominant role for this enzyme in the consumption of NO• under aerobic conditions, a result that is consistent with previous studies of NO• sensitivity in *E. coli* [19]–[22]. To quantitatively investigate the contribution of Hmp to NO• consumption, we calculated the cumulative, time-dependent distribution (overall and intracellular) of NO• for wild-type *E. coli* treated with DPTA using the optimized parameter values (Figures 2C and 2D). The simulated distributions predicted that autoxidation of NO• in the media accounts for the majority of NO• removal shortly after DPTA addition, with loss to the gas phase comprising most of the remaining flux. By 45 min after delivery, the model predicted that cellular consumption of NO• had accumulated to match that of gaseous loss, and after 1 h became the primary sink. The predicted concentration of NO• dropped rapidly to submicromolar levels at 43 minutes post-dose, where it remained for the duration of the simulation, as Hmp continued to remove NO• as it was released by DPTA. The majority (78.1%) of the total NO• released by DPTA was predicted to be consumed by the cells, while autoxidation in the media and loss to the gas phase accounted for 13.6% and 8.3% of the total NO• consumption, respectively. Virtually all (99.85%) of the NO• consumed by the cells was predicted to be through Hmp detoxification, with most of the remaining 0.15% through oxidation by O_2_ and O_2_•^−^. Reduction by anaerobic detoxification enzymes (NorV and NrfA) and nitrosylation of [Fe-S] clusters was predicted to account for less than 0.03% and 0.04% of the cellular NO• consumption, respectively. To provide additional experimental evidence in support of these intracellular distributions, we experimentally validated that a mutant lacking the NorV enzyme (Δ*norV*) consumed NO• at the same rate as wild-type under the experimental conditions tested (Figure S6).

**Figure 3:**
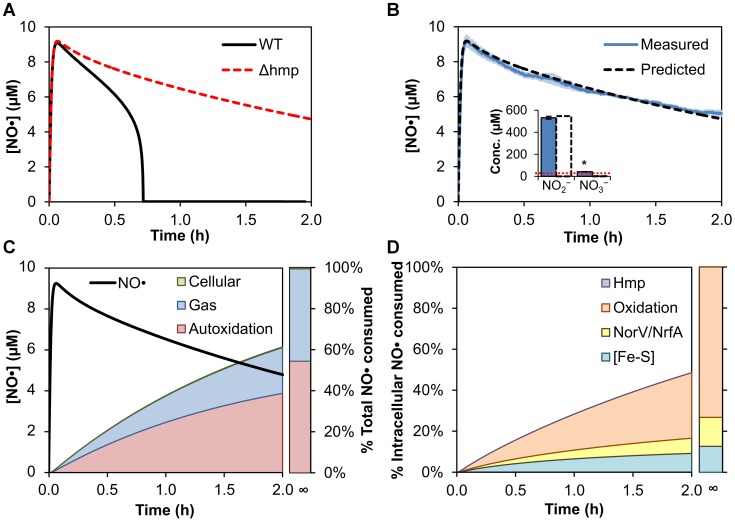
Effect of Δ*hmp* on NO• dynamics in aerobic *E. coli* cultures. **(A)** Simulated NO• profiles for wild-type (solid black line) and Δ*hmp* (dashed red line) cultures following addition of 0.5 mM DPTA. **(B)** NO• concentration following delivery of 0.5 mM DPTA NONOate to a culture of exponential-phase, Δ*hmp E. coli* at an OD_600_ of 0.05 was measured experimentally (solid blue line) and predicted by the model (dashed black line). Error bars (light blue) represent the standard error of the mean for 3 independent experiments. The inset shows the measured (blue bars) and predicted (dashed white bars) NO_2_^−^ and NO_3_^−^ concentrations at 10 h after DPTA delivery to the Δ*hmp* culture. Error bars represent the standard error of the mean for 3 independent experiments. The dotted red line represents the limit of detection for the assay, with the asterisk (*) indicating that the measured [NO_3_^−^] was negligible, as it did not differ significantly from the detection limit (one-sample *t*-test, 95% confidence). **(C)** Simulated NO• concentration profile (black line) and corresponding cumulative distribution of total NO• consumption following addition of 0.5 mM DPTA to Δ*hmp E. coli*. **(D)** Predicted cumulative distribution of intracellular NO• consumption in a Δ*hmp* culture following addition of 0.5 mM DPTA.

Given the importance of Hmp to the removal of NO•, we assessed the predictive power of the model by determining whether it could accurately predict NO• dynamics in a Δ*hmp* mutant culture. We simulated a Δ*hmp* mutant by fixing the Hmp expression rate to zero. All other model parameter values were left unchanged. As expected, the removal of Hmp was predicted to have a considerable effect on the cells' ability to remove NO• from the environment (Figure 3A). Although the [NO•] curve simulated for the Δ*hmp* culture closely matched that predicted for wild-type at early times (0 to ∼10 min) after DPTA delivery, it started to diverge rapidly as Hmp began to dominate the consumption of NO• in the wild-type culture (Figure 2C). The model predicted that the wild-type culture would quickly consume NO• to reach a submicromolar NO• concentration by 43 min, while the concentration of NO• in the Δ*hmp* culture would gradually decline, requiring over 6.4 hours to achieve submicromolar levels. In contrast to wild-type cultures where it was predicted that most NO• would be converted to NO_3_^−^ by Hmp, the model predicted that the majority of NO• in Δ*hmp* cultures would be converted to NO_2_^−^ through autoxidation (Figure 3C). To experimentally confirm the Δ*hmp* model predictions, we measured the concentration of NO• in a Δ*hmp* culture after treatment with 0.5 mM DPTA under identical conditions as wild-type (Figure 3B). In addition, we measured NO_2_^−^ and NO_3_^−^ in the Δ*hmp* culture at 10 h post-dose, when it was predicted that over 99% of the donor had dissociated. The model-predicted NO• concentration curve and final NO_2_^−^ and NO_3_^−^ concentrations were in excellent agreement with the experimental data (without further optimization of any parameters) (Figure 3B), validating the ability of the model to make accurate predictions regarding major perturbations to the system.

To further investigate NO• clearance from the Δ*hmp* culture, we simulated the corresponding intracellular distribution of NO• (Figure 3D). In the Δ*hmp* culture, consumption of NO• by cells was predicted to account for less than 1% of the total NO• delivered, compared to the 78.1% for wild-type cells. Over 73% of the NO• that was consumed through intracellular pathways was predicted to be by reaction with O_2_ or O_2_•^−^, while anaerobic enzymatic reduction (NorV and NrfA) and [Fe-S] nitrosylation accounted for the remaining 14.1% and12.6%, respectively (Figure 3D).

### Analysis of experimentally-accessible model parameters

**Figure 4:**
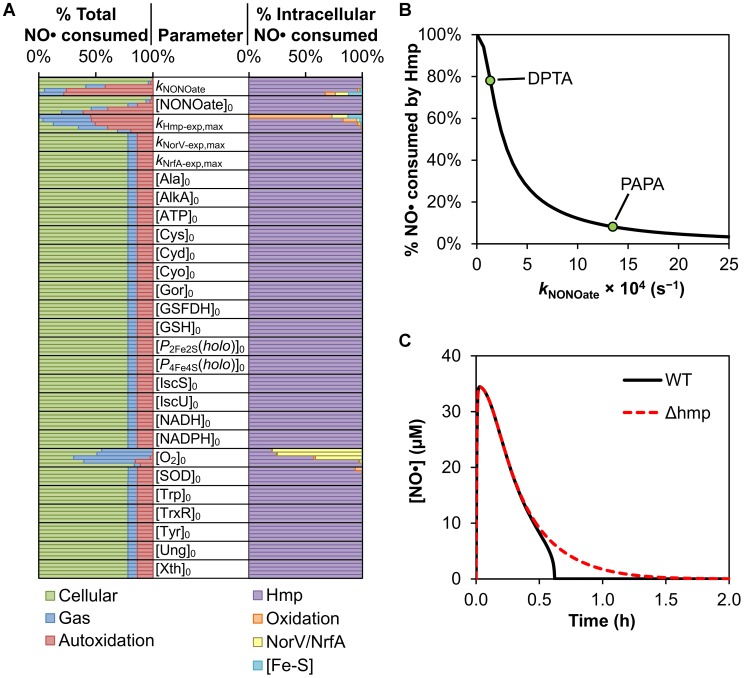
Effect of experimentally-accessible parameters on NO• dynamics. **(A)** Predicted total (left column) and intracellular (right column) NO• distributions corresponding to variation in 27 experimentally-accessible parameters. Each parameter name is listed adjacent to the 5 total and intracellular NO• distributions obtained as a result of varying that parameter logarithmically within its physiological range. The distributions are ordered such that the parameter is increasing in value from top to bottom. **(B)** Percentage of total NO• predicted to be consumed by Hmp as a function of NO• donor release rate. Points (green circles) correspond to the release rates measured for DPTA and PAPA. **(C)** Simulated NO• concentration profile for wild-type (solid black line) and Δ*hmp* (red dashed line) cultures treated with 0.5 mM PAPA.

After validating the model, we sought to identify parameters that control the NO• distribution in *E. coli* cultures. We focused on experimentally-accessible model parameters to enable experimental validation of predictions. To identify control parameters, we performed a parametric analysis (Materials and methods) to assess the effect of varying each parameter on the distribution of NO•. Varied parameters included enzyme concentrations or maximum expression rates, initial concentration and release rate of the NO• donor, O_2_ concentration in the environment, and intracellular concentrations of GSH, amino acids, and energy metabolites (Figure 4A, Table S6). In addition to Hmp expression, the parametric analysis revealed NO• donor concentration and release rate, as well as O_2_ concentration, as important parameters governing the distribution of NO• consumption. Anaerobic NO• detoxification enzymes became the dominant mode of NO• removal within *E. coli* at lower O_2_ concentrations due to the loss of Hmp NO• dioxygenase activity, a decrease in the O_2_-mediated deactivation of NorV, and reduced repression of NrfA expression. The lower O_2_ concentration also decreased the rate of NO• autoxidation in the media, leaving intracellular reactions and escape to the gas phase as the two primary modes of NO• removal. Although it had little impact on the total NO• distribution, removing superoxide dismutase activity resulted in a small, but noticeable increase in the fraction of intracellular NO• consumed through reaction with O_2_•^−^ to form ONOO^−^. Interestingly, the model predicted that higher donor release rates decrease the utility of Hmp in detoxifying NO• (Figure 4B). This decrease can be attributed to the higher NO• concentrations achieved with faster release rates, which in turn enhance substrate inhibition due to the binding of NO• to the Hmp active site before O_2_ [23].

**Figure 5:**
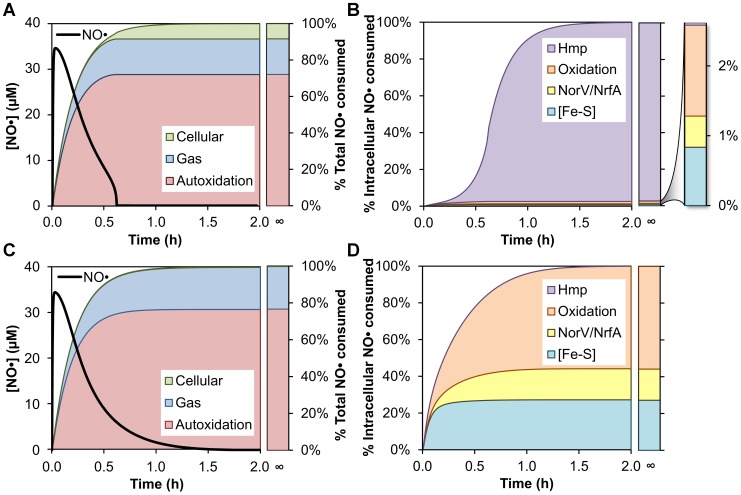
Increasing the NO• delivery rate alters the dynamics and distribution of NO• consumption. **(A)** Simulated NO• concentration curve (black line) and corresponding cumulative distribution of total NO• consumption in a wild-type culture following addition of 0.5 mM PAPA. **(B)** Predicted cumulative distribution of intracellular NO• consumption in a wild-type culture following addition of 0.5 mM PAPA. **(C)** Simulated NO• concentration curve (black line) and corresponding cumulative distribution of total NO• consumption in a Δ*hmp* culture following addition of 0.5 mM PAPA. **(D)** Predicted cumulative distribution of intracellular NO• consumption in a Δ*hmp* culture following addition of 0.5 mM PAPA.

To further examine the effect of donor release rate on model dynamics, we simulated delivery of NO• to cultures at an increased rate, where Hmp contribution to NO• consumption was predicted to be largely reduced. The initial concentration of donor was maintained at 0.5 mM, but the release rate was increased from 1.34×10^−4^ s^−1^ (1.4 h half-life, DPTA) to 1.35×10^−3^ s^−1^ (8.6 min half-life), the measured rate for the NO• donor propylamine propylamine (PAPA) NONOate (Figure S7, Text S1). We performed simulations for wild-type and Δ*hmp* cultures, and generated the corresponding NO• concentration profiles (Figure 4C). The strong influence of NO• delivery kinetics on model dynamics are readily apparent when comparing the NO• concentration profiles simulated for PAPA (Figure 4C) with those for DPTA (Figure 3A). The faster release rate of PAPA predicted a peak NO• concentration nearly four times that of DPTA (34 µM compared to 9 µM, respectively), and a large increase in similarity between the simulated wild-type and Δ*hmp* [NO•] curves was observed. Although the predicted NO• concentration in the PAPA-treated wild-type culture dropped rapidly to submicromolar levels at a time similar to that of DPTA (37 min and 43 min, respectively), Δ*hmp* entered this regime after 1.2 h when treated with PAPA, compared to the 6.4 h predicted for DPTA. We simulated the corresponding NO• distributions for PAPA-treated cultures to examine the participation of the different pathways in NO• removal. The elevated NO• concentrations simulated for the faster-releasing PAPA greatly increased flux through various consumption pathways, where over 99% of the total NO• consumption was predicted to occur within the first hour after dose for both wild-type and Δ*hmp* (Figures 5A and 5C, respectively). The activity of Hmp, however, was attenuated by the higher NO• concentration due to substrate inhibition (see Text S1), reducing its ability to participate in detoxification. When Hmp activity was restored and became the most rapid NO• removal pathway after ∼30 minutes, simulation results showed that over 90% of the total NO• had already been consumed through autoxidation and gas transfer pathways (Figure 5A). As a result, the fraction of total NO• consumed by cellular pathways in the wild-type culture was predicted to decrease by nearly 10-fold (78.1% to 8.4%) due to the increased NO• delivery rate (compare Figures 2C and 5A). When treating with DPTA, the NO• concentration profile and distribution simulated for the Δ*hmp* mutant (Figure 3B) were observed to differ greatly from those of wild-type (Figure 2C), but were significantly more similar to wild-type when using PAPA as the donor (Figures 5A and 5C) due to the large reduction in Hmp-mediated NO• consumption predicted for the wild-type culture. The intracellular distribution simulated for wild-type treated with PAPA (Figure 5B) was still dominated by Hmp, despite its large reduction in activity. However, the proportion of intracellular NO• consumed through pathways other than Hmp was predicted to increase by over 15-fold (0.15% to 2.6%) upon increasing the NO• delivery rate, suggesting that these other pathways maintain activity while Hmp is inhibited. Thus, the reduction in Hmp activity predicts a 15-fold increase in contribution by other intracellular pathways to the removal of NO• within the cell, including damage to biomolecules such as [Fe-S] clusters.

### Experimental validation of the dependence of Hmp on NO•-delivery kinetics

**Figure 6:**
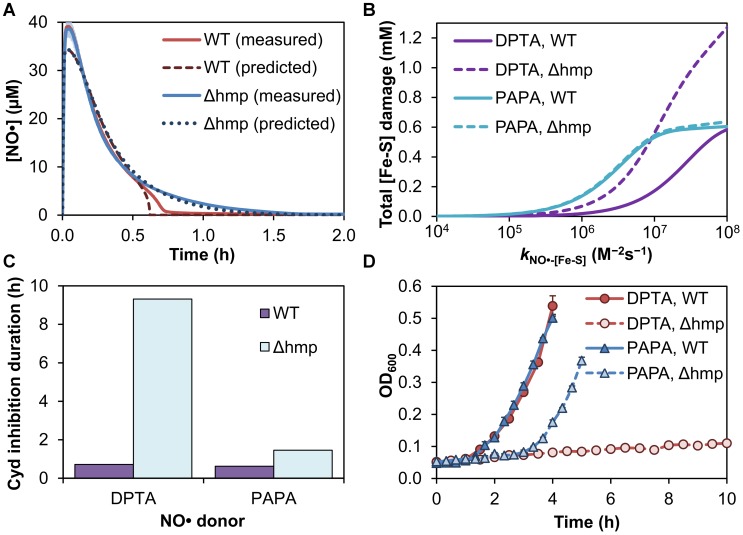
The utility of Hmp for NO• consumption and tolerance decreases with increased NO• delivery rate. **(A)** Experimentally measured NO• concentration profiles following addition of 0.5 mM PAPA to a culture of wild-type (solid red line) or Δ*hmp* (solid blue line) *E. coli* at an OD_600_ of 0.05. Lines depict the mean of three independent experiments, and error bars (light red and light blue for wild-type and Δ*hmp*, respectively) represent the standard error of the mean. Also shown are the corresponding model-predicted NO• concentration profiles for wild-type (dark red dashed line) or Δ*hmp* (dark blue dotted line) cultures. **(B)**. Predicted cumulative concentration (per unit cellular volume) of [2Fe-2S] and [4Fe-4S] clusters damaged following DPTA (purple) or PAPA (teal) treatment of wild-type (solid lines) and Δ*hmp* cultures (dashed lines) is plotted as a function of the model parameter governing the rate of [Fe-S] nitrosylation by NO• (*k*_NO•-[Fe-S]_). **(C)** Predicted durations of cytochrome *bd* (Cyd) inhibition by NO•, defined as the time required for the percentage of NO•-bound Cyd to drop below 50% of the total Cyd concentration. **(D)** Experimentally measured growth curves (quantified by OD_600_) for wild-type and Δ*hmp* cultures following treatment with 0.5 mM of DPTA or PAPA demonstrate more comparable duration of bacteriostasis between wild-type and Δ*hmp* for treatment with PAPA than DPTA. Due to the faster NO• delivery kinetics associated with PAPA, OD_600_ readings were taken more frequently (20 min intervals) than with DPTA (30 min intervals). After addition of DPTA to Δ*hmp* cells, growth resumption was not observed within the 10 h timeframe of the experiment.

To experimentally validate the prediction that the utility of Hmp decreases as the delivery rate of NO• increases, we measured and compared the ability of wild-type and Δ*hmp* to remove NO• from the culture when dosed with PAPA. We observed excellent agreement between model-predicted and experimentally-measured NO• concentration profiles for the addition of PAPA to wild-type and Δ*hmp* cultures, with no further optimization of model parameters (Figure 6A). The peak concentration of NO• was underestimated by approximately 10%, which was also observed when measuring NO• release from PAPA in media without cells (Figure S7), suggesting that the disagreement was not associated with cellular parameters. In addition, the rate of NO• clearance by the wild-type cells was slightly overestimated. This could originate from the treatment of Hmp expression in the model, where a more extensive implementation of its governing regulatory network may improve the accuracy of the simulated transcriptional response of *hmp* expression to elevated levels of NO•. As predicted, the measured difference in time required to remove NO• from the culture between wild-type and Δ*hmp* was small for PAPA (0.6 h difference in time to reach submicromolar levels), highlighting the decreased utility of Hmp under conditions of more rapid NO• release. These results demonstrate that the model can accurately identify parameters that control the distribution of NO• in bacterial cultures, and quantify the impact of their manipulation.

In addition to NO• removal from the cell interior and surrounding environment, the model can be used to calculate the extent to which NO• affects various cellular targets, including [Fe-S] nitrosylation [24]–[26] and cytochrome inhibition [27], [28]. Therefore, we utilized the model to evaluate the protective effect of Hmp with respect to [Fe-S] cluster damage (Figure 6B) and cytochrome *bd* inhibition (Figure 6C). Due to the wide range of reaction rates reported for the nitrosylation of [Fe-S] clusters by NO• (*k*_NO•-[Fe-S]_), the parameter value was varied across this range when predicting the extent of [Fe-S] damage. Simulated exposure of wild-type and Δ*hmp E. coli* to 0.5 mM DPTA predicted a 2- to 4-fold reduction in the total concentration of [Fe-S] clusters damaged as a result of Hmp activity. When simulations were repeated for PAPA, however, the total [Fe-S] damage predicted for wild-type and Δ*hmp* cultures differed by a maximum of 5%, in agreement with the predicted dependence of Hmp utility on NO• release rate. Furthermore, the duration of NO•-mediated cytochrome *bd* inhibition following DPTA treatment was predicted to greatly increase for the Δ*hmp* culture relative to wild-type, requiring over 9 h (compared to 0.7 h for wild-type) for the concentration of NO•-bound cytochromes to drop below 50% of the total. Treatment with PAPA resulted in more similar cytochrome inhibition between the strains, with durations of 0.6 h and 1.5 h predicted for wild-type and Δ*hmp*, respectively. Collectively, the results from these damage descriptors, in addition to the rate and distribution of NO• consumption, predicted a greater similarity in recovery from bacteriostasis between wild-type and Δ*hmp* when treated with PAPA than with DPTA. To test the prediction, we monitored the optical density (OD_600_) of each strain following treatment with 0.5 mM DPTA or PAPA (Figure 6D). In agreement with the prediction, the duration of NO•-induced stasis was more similar between wild-type and Δ*hmp* strains when using a faster NO• donor. Growth inhibition of Δ*hmp* following PAPA treatment was less severe than that observed for DPTA, where cells exited stasis less than 2 h after wild-type, compared to over 10 h for DPTA.

### Experimental validation of the microaerobic utility of Hmp

**Figure 7:**
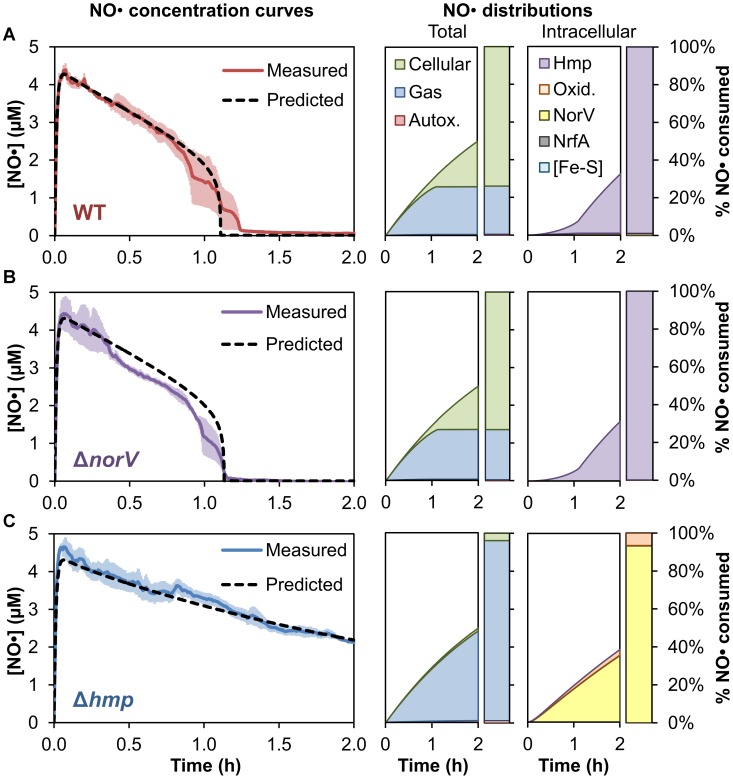
NO• dynamics and distribution in *E. coli* cultures under microaerobic conditions. Shown along the left are the simulated and experimentally-measured NO• concentration profiles for **(A)** wild-type, **(B)** Δ*norV*, and **(C)** Δ*hmp* cultures following addition of 0.5 mM DPTA, where the O_2_ concentration was maintained at 35 µM by bubbling with N_2_. Error bars (light red, light purple, and light blue for wild-type, Δ*norV*, and Δ*hmp*, respectively) represent the standard error of the mean for at least 3 independent experiments. Shown directly to the right of the NO• concentration profiles are the corresponding predicted cumulative distributions of total (left) and intracellular (right) NO• consumption.

Hmp is considered the major aerobic enzyme responsible for NO• detoxification [19], [21], [29], whereas NorV is considered the major anaerobic detoxification system [22], [30], [31]. Surprisingly, the parametric analysis suggested that Hmp remains dominant at O_2_ concentrations as low as 25 µM (∼14% air saturation [32]) (Figure 4A). To experimentally confirm that this was the case, we adjusted the experimental setup by adding N_2_-bubbling at a rate of 1 ml/s. In the presence of wild-type *E. coli* at an OD_600_ of 0.05, an O_2_ concentration of 35 µM was achieved and maintained constant throughout the time course of a DPTA experiment (Figure S8). This concentration was over 5-fold less than air-saturated media (185 µM), but also above the 25 µM used in the parametric analysis. Due to the adjustment in experimental conditions, the model was similarly optimized for NO• dynamics from microaerobic wild-type *E. coli* cultures (Material and methods), and found to capture the data very well (Figure 7A). We note that N_2_-bubbling increased fluctuations in the NO• measurements, but the increased error was minor compared to the range of NO• concentrations investigated. Using the optimized model, we predicted the effect of genetic deletions of *norV* and *hmp* on the NO• dynamics. Consistent with the previous parametric analysis, NorV was identified as a negligible consumption pathway under microaerobic conditions (35 µM O_2_), whereas Hmp was identified as the major NO• sink. These predictions were experimentally validated, and the results are presented in Figures 7B and 7C. These data demonstrate that the model is useful for studying sub-aerobic environmental conditions, and that the switch between Hmp-dominated and NorV-dominated NO• consumption regimes occurs at very low O_2_ concentrations.

The corresponding extracellular and intracellular NO• distributions for wild-type, Δ*hmp*, and Δ*norV* under microaerobic conditions were simulated, and are presented in Figure 7. Loss of NO• to the gas phase was predicted to largely increase for all strains (26% and 27% of the total NO• consumption for wild-type and Δ*norV*, respectively, and 95% for Δ*hmp*), due to the increased air-liquid surface area caused by the bubbling of N_2_ through the culture, as well as the reduced rate of autoxidation. Autoxidation was predicted to have negligible NO• consumption activity compared to the cellular and gas transport pathways (0.5% of the total for wild-type and Δ*norV*, and 1.2% for Δ*hmp*), owing to the reduced O_2_ concentration, as well as the lower peak NO• concentration (∼4.5 µM for all strains) than was achieved under aerobic, non-bubbling conditions using DPTA (∼8–10 µM). Cellular consumption of NO• was still predicted to be the greatest sink of NO• for the wild-type and Δ*norV* strains (accounting for 74% and 73% of the total consumption, respectively), but only a minor pathway in the Δ*hmp* culture (3.8%). The intracellular distributions (Figure 7) for wild-type and Δ*norV* cultures were still predicted to be dominated by Hmp detoxification (both exceeding 98% of intracellular NO• consumed by Hmp), as was seen under aerobic conditions. The NO• consumed by Δ*hmp* cells, however, was now predicted to occur primarily through NorV reduction (93% of the intracellular NO•), compared to the 14% contribution predicted for Δ*hmp* in aerobic conditions. Overall, the simulation results predicted Hmp to be the primary mode of NO• consumption under O_2_ concentrations as low as 35 µM, but suggested an increased role of NorV reduction in the event that Hmp detoxification becomes unavailable.

## Discussion

NO• is a critical antimicrobial of the innate immune response whose utility originates from its ability to diffuse through cellular membranes [33], deactivate bacterial enzymes [26], inhibit respiration [28], and react with O_2_ and O_2_•^−^ to yield the reactive nitrogen species, NO_2_•, N_2_O_3_, N_2_O_4_, and ONOO^−^ [24]. The biochemical reaction network of NO• includes both spontaneous and enzymatic reactions involving many short-lived species that decompose to several common end-products [4]. Increasing the complexity of this system is the continuous degradation and repair of damaged biomolecules, which regenerates targets for NO• and its reactive intermediates [34]. A quantitative description of how NO• distributes among these many pathways is critical to understanding immune function and pathogenesis, as well as to designing NO•-based and NO•-synergizing therapeutics [35]–[37]. However, the complexity of the NO• reaction network renders exhaustive experimental monitoring infeasible, and interpretation of measurements difficult [4], [6]. To address these challenges, experimentally-informed computational models are required to explore the NO• reaction network.

Though several kinetic models have been developed to study the chemistry of NO• in biological systems, of which the majority are mammalian, none have had sufficient breadth and depth to address the full range of effects of NO• exposure [1], [38]. The model presented here is far more comprehensive than those constructed previously, incorporating the damage, modification, and repair of biomolecules, as well as enzymatic detoxification and transcriptional control. These functionalities allow focused investigation of intracellular components of the NO• network, such as [Fe-S] cluster and DNA damage, but also culture-wide prediction of the NO• distribution. We validated the utility of the model by demonstrating that it can reproduce NO• dynamics in a bacterial culture, make accurate predictions regarding large perturbations to the system, and identify parameters that control the distribution of NO• in bacterial cultures. Specifically, model simulations predicted that NO• autoxidation and Hmp-catalyzed detoxification were the primary sinks for NO• consumption in aerobic wild-type *E. coli* cultures. Oxidation of NO• has been shown in the past to be a major contributor to the consumption of NO• under certain conditions [3], and the dominant role of Hmp in aerobic detoxification is in agreement with previous studies that have demonstrated its importance in tolerating NO• stress [21]–[23]. In addition, we used the model to (1) uncover a novel dependency of Hmp utility on the NO• delivery rate, and (2) discover that Hmp is the dominant cellular NO• detoxification system at dissolved O_2_ concentrations as low as 35 µM (microaerobic). Both of these predictions were validated experimentally, thereby demonstrating the utility of the model for the study of NO• metabolism. Specifically, when treated with a fast-releasing NO• donor (PAPA), the consumption of NO• and recovery from bacteriostasis was far more similar between wild-type and Δ*hmp E. coli* than with a slower NO• donor (DPTA). This effect arises from substrate inhibition of the Hmp active site caused by high NO•/O_2_ concentration ratios and the time required to synthesize Hmp [23]. An effect of NO• delivery on its toxicity has been observed previously in a mammalian system [39], [40], where it was shown that killing of human lymphoblastoid cells (TK6 and NH32) was a function of both NO• concentration and cumulative dose. Here, we have demonstrated an influence of NO• delivery rate on the dynamics of NO• consumption and recovery in bacterial cultures, and also offered a detailed, mechanistic description of the observed dependence. In addition, we discovered that Hmp remains the major cellular detoxification system at dissolved O_2_ concentrations as low as 35 µM. This effect originates from the strong induction of Hmp expression upon NO• exposure even under anaerobic conditions [41], [42], and the rapid O_2_-mediated deactivation of NorV, the alternative NO• detoxification system that has been previously identified as critical for resisting NO• stress under anaerobic conditions [22], [31]. These data demonstrate the flexibility of this method to different environmental conditions (microaerobic), and provide support for the role of Hmp as a virulence factor [43], [44], since O_2_ concentrations at infections sites/in macrophages and neutrophils are typically hypoxic (less than 50 µM O_2_ [4], [45], [46]). Interestingly, both NorV- and Hmp-type enzymes have been found to be virulence factors for numerous organisms [36], [47]–[50], and thus a quantitative understanding of the conditions under which each contributes to NO• clearance would be valuable for the study of their importance to virulence.

The work presented here demonstrates the predictive accuracy and utility of a comprehensive model of NO• metabolism in *E. coli*. The scope of this model allows for detailed, quantitative exploration of numerous NO• network features and environmental conditions, including future investigations of the roles of O_2_ concentration and indirect NO• delivery, such as that observed for S-nitrosothiols [41]. Further, this model will prove useful for the optimization of NO•-synergizing and NO•-based therapeutics, which are being investigated as antibiotic alternatives for the treatment of both gram-positive and gram-negative infections, including those caused by *Mycobacterium tuberculosis*, *Staphylococcus aureus*, *Pseudomonas aeruginosa*, *E. coli*, and *Acinetobacter baumannii* [35]–[37]. Such therapies include NO•-releasing nanoparticles [36], NO•-releasing dressings [37], and rhodanines, which kill non-replicating mycobacteria through the potentiation of host-derived NO• [35]. Interestingly, the study by Sulemankhil and colleagues identified NO• release rate and dosage as important parameters governing the effectiveness of the examined dressings. The modeling approach presented here could provide a more quantitative understanding of how these potential therapeutics neutralize pathogens, and would prove useful for identifying methods to increase their potency through the quantitative identification of the NO• distribution pathways used by specific organisms. To achieve this potential utility, the modeling method described here must be adapted for use in organisms other than *E. coli*. To do this, the enzymatic reactions within the model would need to be removed, replaced, or augmented based on the systems harbored by the pathogen of interest, and uncertain parameters would need to be identified by training the model on experimental data, as performed here. In the event that an important reaction is missing from a model, stable NO• end products (such as NO_2_^−^ and NO_3_^−^) would be measured and both metabolic databases and the organism's genome would be mined for model additions capable of capturing the experimental data. Potential additions would then be experimentally validated by measuring *in vitro* kinetics of samples purified from cultures of interest. Execution of these steps will produce models of NO• metabolism in pathogens, that will mirror utility and capabilities achieved by the kinetic platform described here.

## Materials and Methods

### Bacterial strains

All strains used in this study were *E. coli* K-12 MG1655. The Δ*hmp* and Δ*norV* mutants were obtained from the Keio collection [51], and transferred into the MG1655 background using the P1 phage method. Proper chromosomal integration and absence of gene duplication were checked by PCR. The *hmp* primers used were 5′-CCGAATCATTGTGCGATAACA-3′ (forward) and 5′-ATGATGGATACTTTCTCGGCAGGAG-3′ (reverse) for accurate integration, and 5′- TCCCTTTACTGGTGGAAACG-3′ (forward) and 5′-CACGCCCAGATCCACTAACT-3′ (reverse) for gene duplication. The *norV* primers used were 5′-CCAGCACATCAACGGAAAAA-3′ (forward) and 5′-ATGATGGATACTTTCTCGGCAGGAG-3′ (reverse) for accurate integration, and 5′-GACTGGGAAGTGCGTGATTT-3′ (forward) and 5′-CGGAAGCGTAAACCAGTCAT-3′ (reverse) for gene duplication.

### Chemicals

NO• donors (Z)-1-[N-(3-aminopropyl)-N-(3-ammoniopropyl)amino]diazen-1-ium-1,2-diolate (DPTA NONOate) and (Z)-1-[N-(3-aminopropyl)-N-(n-propyl)amino]diazen-1-ium-1,2-diolate (PAPA NONOate) were purchased from Cayman Chemical Company. All other chemicals and reagents were purchased from Sigma Aldrich or Fisher Scientific, unless otherwise noted.

### Cell growth and NO• consumption assays

*E. coli* from a frozen −80°C stock were inoculated into 1 ml of fresh LB broth and grown for 4 hours at 37°C and 250 r.p.m. 10 µl of the LB culture were used to inoculate 1 ml of MOPS minimal media (Teknova) containing 10 mM glucose. The minimal glucose culture was grown at 37°C and 250 r.p.m. overnight (16 h) and used to inoculate 20 ml fresh MOPS glucose (10 mM) in a 250 ml baffled shake flask to a final OD_600_ of 0.01. The flask culture was grown at 37°C and 250 r.p.m. to exponential phase (OD_600_ = 0.2), at which point 4 ml was transferred to separate microcentrifuge tubes in 1 ml aliquots and centrifuged at 15,000 r.p.m. for 3 min at 37°C. To remove the culture media, 980 µl of the supernatant was removed and cell pellets were resuspended in 1 ml of pre-warmed (37°C) 10 mM MOPS glucose media. Samples were combined in a 15 ml Falcon tube, and returned to the shaker (37°C, 250 r.p.m.). After 5 minutes, the resuspended culture was diluted to an OD_600_ of 0.03 in fresh, pre-warmed (37°C) MOPS glucose (10 mM) in a 50 ml Falcon tube with a final culture volume of 10 ml. The culture was stirred with a sterilized magnetic stirring bar, and immersed in a stirred water bath to maintain the temperature at 37°C. Growth was monitored until the OD_600_ reached a value of 0.05 (approximately 45 minutes after diluting to OD_600_ of 0.03), at which time the NO• donor solution (DPTA or PAPA) was added. On the day of use, the NONOate powder was dissolved in a chilled (4°C), sterile solution of 10 mM NaOH in deionized H_2_O, and stored on ice prior to delivery. After NONOate delivery, every half-hour (DPTA) or twenty minutes (PAPA), 75 µl aliquots were removed to measure the OD_600_ (Synergy H1 Microplate Reader, BioTek Instruments, Inc.). The concentration of NO• in the culture was monitored continuously over the course of the experiment using an ISO-NOP NO• sensor (World Precision Instruments, Inc.). The electrode was calibrated daily, prior to use, according to the manufacturer's specifications.

The microaerobic NO• consumption assay was performed using the same procedure, except N_2_ bubbling was included to reduce the dissolved O_2_ concentration. Immediately following the dilution of cells to an OD_600_ of 0.03 in the 50 ml Falcon tube, N_2_ gas (99.998% pure) was bubbled into the culture through a sterile pipet tip at a constant flow rate of 1 ml/s. The O_2_ concentration was observed to drop quickly and stabilize at approximately 19% air saturation (35 µM) within 15 minutes of initiating the N_2_ bubbling, where it remained for the duration of the experiment. The concentration of O_2_ was monitored continuously to ensure stable conditions throughout the assay (Figure S8).

### O_2_ measurements

The concentration of dissolved O_2_ was measured using the FireStingO2 fiber-optic O_2_ meter with the OXF1100 fixed needle-type minisensor (PyroScience GmbH). The sensor was calibrated according to the manufacturer's specifications, and the signal was automatically compensated for temperature fluctuations using the TDIP15 temperature sensor (PyroScience GmbH) during all O_2_ measurements.

### NO_2_^−^ and NO_3_^−^ quantification

The concentration of NO_2_^−^ and NO_3_^−^ were measured using the Nitrate/Nitrite Colorimetric Assay Kit from Cayman Chemical Company, following the manufacturer's instructions. Briefly, Griess reagents were added to diluted samples to convert the NO_2_^−^ to a purple azo compound, and quantified by measuring the absorbance at 540 nm using a microplate reader [52]. A calibration curve was generated using varying dilutions of a standard NO_2_^−^ solution. The total NO_2_^−^+NO_3_^−^ concentration of the samples were obtained by first converting the NO_3_^−^ to NO_2_^−^ using nitrate reductase, and then treating with Griess reagents. The NO_3_^−^ concentration in the samples was calculated as the difference between the total NO_2_^−^+NO_3_^−^ concentration and the NO_2_^−^ concentration. All samples were measured in triplicate.

### Model development

#### Framework

The model simulates the dynamics of intracellular metabolites and cellular components upon exposure to NO• using a system of differential mass balances,
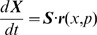
where ***X*** is the vector of species concentrations, and ***S*** is a matrix of stoichiometric coefficients describing all model reactions. Reaction rates, defined by vector ***r***, are a function of the relevant species concentrations *x*, and the associated kinetic parameter(s) *p*. Table S1 lists the metabolites and enzymes considered in the model, as well as their initial concentrations. Table S2 lists reactions and rate constants for reactions governed by elementary-type rate equations (for example, *r* = *k*[A][B]), while Table S3 presents reactions, rate expressions, and associated kinetic parameters for reactions with more complex rate equations. The following sections briefly describe specific reactions and assumptions of the model, whereas more detail can be found in Text S1.

#### Compartmentalization

The model was partitioned into intracellular and extracellular compartments to facilitate experimental validation, as measurements inherently involve the culture media. Given the ability of NO• and O_2_ to diffuse freely across membranes, we assumed equal intracellular and extracellular concentrations [33], [53]. To account for the difference in volume between the compartments, the NO• and O_2_ mass balances were scaled according to their relative volumes,
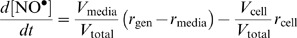
where *V* is the total or compartment-specific volume, *r*_gen_ is the rate of generation of NO• in the media, and *r*_media_ and *r*_cell_ are the rates of extracellular and intracellular NO• consumption, respectively. The volume fractions were calculated as a function of OD_600_, assuming an OD-specific cell concentration of 11.1±1.1×10^8^ cells.ml^−1^.OD^−1^, and a single-cell volume of 3.2×10^−15^ L [54]. In our experiments, the culture was treated with NO• when it reached an OD_600_ of 0.05, which corresponds to a cell volume fraction of 1.78×10^−4^.

#### Intracellular pH

The intracellular pH was assumed to be 7.6, reflective of bacteria growing in a neutral medium [55]. The pH was used to select appropriate reaction rate constants, or derive them from related kinetic parameters, such as pKa, when necessary (Tables S2, S3).

#### NO• delivery

The delivery of NO• to the system was modeled as originating from an NO• donor (such as DPTA or PAPA), where the initial concentration of the donor species (in the extracellular compartment) was set accordingly. The release of NO• from the NONOates was modeled according to first-order decay kinetics, releasing two moles of NO• per mole of parent compound [56]. First-order rate constants were experimentally measured for DPTA and PAPA in our experimental system (Figures S3 and S7, Text S1). Although NO• generation is simulated as originating from chemical donors in this study, the model can just as easily be modified to represent NO• delivery to the system by another method.

#### NO• autoxidation

In aqueous solutions, NO• will undergo autoxidation at a rate that is second-order in NO• and first-order in O_2_ to generate nitrogen dioxide (NO_2_•), which may combine with another molecule of NO• to form nitrous anhydride (N_2_O_3_) [57], [58]. These oxidized forms of NO• contribute to a number of cytotoxic effects such as DNA damage and thiol nitrosation [59], [60]. Equations 1–7 of Table S2 describe the NO• autoxidation reactions used in this study.

#### Enzymatic NO• detoxification

The main enzymatic NO• detoxification systems identified in *E. coli* are NO• dioxygenase (Hmp) [19], flavorubredoxin reductase (NorV) [31], and periplasmic formate-dependent nitrite reductase (NrfA) [61]. Hmp is the primary enzyme responsible for detoxifying NO• under aerobic conditions via dioxygenation to NO_3_^−^ [19]–[22], though it has been shown to possess low levels of NO• reductase activity in the absence of O_2_ [23], [62]. NorV confers protection from NO• in anaerobic environments, where it catalyzes the reduction of NO• to N_2_O at a rate orders of magnitude greater than the Hmp-mediated reduction [22], [63]. The reductase activity of NorV deteriorates rapidly upon exposure to oxygen, thus preventing its contribution to NO• detoxification in the presence of O_2_ [22]. The primary role of NrfA is the respiratory reduction of NO_2_^−^; however, NrfA has also been shown to catalyze the 5-electron reduction of NO• to NH_4_^+^ in the presence of NO_2_^−^ or NO_3_^−^ [61], [64]. NrfA expression is under control of the O_2_-responsive regulator FNR, restricting its role to anaerobic environments [65], [66]. Reactions 98–126 in Table S2 (Hmp), and reactions 173–174 (NorV) and 175 (NrfA) in Table S3 describe the enzyme-mediated detoxification reactions, while equations 177–179 in Table S3 and reactions 131–150 in Table S2 govern the expression and degradation of the enzymes, respectively.

#### Thiol S-nitrosation and denitrosation

Protein-bound and low molecular weight (LMW) thiols are subject to modification upon exposure to NO•, forming products such as S-nitrosothiols and thiyl radicals [67]–[69]. NO• itself does not react directly with thiols at a rate that is physiologically relevant [70]; instead, it is the oxidized forms (NO_2_• and N_2_O_3_) that are primarily responsible for the nitrosation of thiols [71], [72]. Abundant LMW thiols, such as GSH in *E. coli*, can serve as a protective sink for RNS [24], [73], but modification of protein-bound thiols may affect protein function [67], [68]. Repair of nitrosated thiols can occur spontaneously through mechanisms such as transnitrosation, whereby a damaged protein transfers the nitroso group to a LMW thiol [74]. Enzymatic processes have also been identified; for example, denitrosation can be catalyzed by glutathione-dependent formaldehyde dehydrogenase (GS-FDH), which has a high specificity for GSNO [75]. Reactions 32–64, 76, 81, and 83–84 of Table S2, and reactions 167–170 of Table S3 describe the thiol nitrosation and denitrosation, and thiol oxidation and reduction pathways included in the model.

#### Cytochrome inhibition

The high affinity of NO• for the terminal quinol oxidases (cytochromes *bo* and *bd* in *E. coli*) makes it a potent inhibitor of respiratory function, which can cause bacteriostatic effects even at low NO• concentrations [27], [28], [76]. The inhibition, caused by a reversible binding of NO• to the heme active site, is relieved upon depletion of the local NO• concentration by cellular machinery such as Hmp [21]. Equations 171–172 of Table S3 describe the reactions and equations governing NO•-mediated cytochrome inhibition used in this study.

#### O_2_•^−^ generation and ONOO^−^ formation

O_2_•^−^ is generated within microbes as a byproduct of aerobic respiration, but is generally maintained at low concentrations due to the O_2_•^−^ scavenging activity of superoxide dismutases [53], [77]. ONOO^−^ is a strong oxidant formed when NO• reacts with O_2_•^−^ at a near diffusion-controlled rate, and can have a number of deleterious effects ranging from DNA damage to lipid peroxidation [78]–[80]. Equations 8–9, 12–17, 43, 45, 55–56, 61, 65–67, 127, and 130 of Table S2 describe the O_2_•^−^ and ONOO^−^ associated reactions included in the model.

#### Tyrosine nitration

Tyrosine nitration is a result of nitrosative and oxidative stress, where ONOO^−^ has been implicated as a nitration mediator under physiological conditions [11], [81]. NO• and NO_2_• form 3-nitrosotyrosine and 3-nitrotyrosine, respectively, upon reaction with tyrosyl radicals generated from the oxidation of tyrosine by radicals such as NO_2_•, CO_3_•^−^, and •OH [82], [83]. Equations 62–63 and 69–74 of Table S2 describe the reactions involving tyrosine oxidation and nitration used in this study.

#### Iron-sulfur cluster damage and repair

The disruption of [Fe-S] clusters is known to largely contribute to the bacteriostatic effect of NO• [26], [84]–[87]. NO• reacts quickly with clusters, resulting in the formation of inactive protein-bound dinitrosyl iron complexes (DNICs) and Roussins' red esters (RREs) [87]–[91]. The repair of [Fe-S] clusters in *E. coli* is a complex process (Figure S1) involving extrusion of the damaged cluster from the protein [88], [92]–[94], *de novo* assembly of a cluster on a protein scaffold by the Isc or Suf system [95]–[98], and reinsertion of the new [Fe-S] complex into an apoprotein [99]–[102]. Equations 85–94 of Table S2 and equations 151–154 of Table S3 describe the [Fe-S] cluster damage and repair reactions incorporated into the model.

#### DNA deamination and repair

DNA damage resulting from NO• exposure has been associated with the formation of N_2_O_3_, which can deaminate DNA bases, leading to transition mutations and strand breaks [103]–[106]. The bases adenine (A), cytosine (C), and guanine (G) are deaminated to yield hypoxanthine (hX), uracil (U), and xanthine (X), respectively [103], and are primarily repaired via the base excision repair (BER) system [107]–[109]. In general, the BER pathway (Figure S2) involves glycosydic cleavage of the deaminated base to generate an apurinic/apyrimidinic (AP) site [110]–[113], backbone cleavage and AP site excision by AP endonuclease [114], [115], and nucleotide re-insertion (DNA polymerase I) and ligation (DNA ligase) [116]–[118]. Equations 95–97 of Table S2 and equations 155–166 of Table S3 describe the DNA deamination and repair reactions used in this study.

### Kinetic simulations

All simulation calculations were performed using Matlab (R2012a). The governing set of differential mass balances was integrated using the stiff numerical ODE integrator (*ode15s* function).

### Parameter optimization

Optimization of model parameters was performed in Matlab using the *lsqcurvefit* function, which solves nonlinear least-squares minimization problems. Through an iterative process, the function identified parameter values yielding the lowest sum of squared residuals (SSR) between the experimentally-measured and model-simulated NO• concentration profiles. Since the nonlinearity of the minimization problem gives rise to local minima, we performed 100 independent optimizations, each initialized with a random set of parameter values (within their allowed range).

The parameter optimization procedure was used to determine the values of extracellular parameters specific to our experimental system: NO• donor dissociation (*k*_NONOate_), transfer of NO• to the gas phase (*k*_L_ *a*_NO•_), and the rate of NO• autoxidation (*k*_NO•-O2_). Cell-free growth media was treated with 0.5 mM DPTA under conditions identical to the aerobic NO• consumption assay, and the resulting NO• concentration profile and final (10 h) NO_2_^−^ and NO_3_^−^ concentrations were measured. The optimization yielded values of 1.34×10^−4^ s^−1^ (1.4 h half-life), 4.74×10^−3^ s^−1^, and 1.80×10^6^ M^−2^s^−1^ for *k*_NONOate_, *k*_L_ *a*_NO•_, and *k*_NO•-O2_, respectively (see Text S1 for further detail). Figure S3 demonstrates excellent agreement between the predicted and measured [NO•] curve and final NO_2_^−^ and NO_3_^−^ concentrations when using the optimized parameter values.

Of the cellular-related model parameters, 39 were classified as uncertain due to variability or unavailability in literature (Table S4). A parameter optimization was conducted to identify the set of parameter values yielding the lowest SSR between the simulated and experimentally-measured NO• concentration profile resulting from the addition of 0.5 mM DPTA to an aerobic, exponential-phase culture of wild-type *E. coli*. The predicted [NO•] curve using the optimal parameter set was in excellent agreement with the experimental data (Figure 2A).

For the microaerobic (35 µM O_2_) NO• consumption assay, uncertain parameters were re-optimized for the low-O_2_ environment due to expected changes in cellular properties and the effect of N_2_ bubbling on gas transfer rates. We note that differences in N_2_ bubble properties (such as bubble size and lifetime) caused by the presence of cells prevented the use of cell-free NO• measurements in determining extracellular parameters for this experimental setup. Instead, the simulated O_2_ concentration was fixed to 35 µM based on experimental observations (Figure S8), and the remaining extracellular and uncertain parameters (total of 42 parameters) were simultaneously optimized to best capture the NO• concentration curve measured for wild-type cells treated with DPTA under microaerobic conditions (Table S7). The optimal set of parameter values was able to accurately capture the experimentally-measured NO• dynamics in the microaerobic environment (Figure 7). An individual parametric analysis of the 42 optimized parameters was performed to determine those that had a significant impact (greater than 5% increase in SSR) on the predicted [NO•] curve in the microaerobic environment (Figure S9). Similar to the aerobic parametric analysis, Hmp-associated parameters (*k*_Hmp,NO•-on,_ *k*_Hmp-exp,max_, and *K*_Hmp-exp,NO•_) were found to strongly influence the predicted NO• dynamics. Parameters governing the rate of NONOate dissociation (*k*_NONOate_) and NO• transfer to the gas phase (*k*_L_ *a*_NO•_) also demonstrated substantial control of the [NO•] curve upon variation. Finally, NorV expression (*k*_NorV-exp,max_ and *K*_NorV-exp,NO•_) and inactivation (*k*_NorV-O2_) parameters were found to have a significant impact on the SSR.

### Parametric analyses

Parametric analyses were used to evaluate the influence of model parameters on either the simulated [NO•] curve or the predicted distribution of NO• consumption in the culture. The effect of parameter variation on the [NO•] curve was quantified by the resulting change in SSR between the model-simulated and experimentally-measured NO• concentration profiles. Specifically, parameters were individually varied among 100 evenly-spaced points spanning their allowed range, and the resulting SSR at each parameter value was calculated. The effect of parameter variation on the SSR for aerobic (Figure 2B, Table S4) and microaerobic (Figure S9, Table S7) wild-type *E. coli* cultures was evaluated.

To quantify the effect of varying experimentally-accessible parameters on the predicted distribution of NO• consumption, parameters were individually varied among five logarithmically-spaced values spanning their permitted range (Table S6). Simulations were run for each different parameter set, and the final distribution of NO• consumption among the available pathways (such as autoxidation, transport to the gas phase, Hmp-mediated detoxification, and [Fe-S] damage) was calculated (Figure 4A).

### Comparison with previous NO• models

Three existing models of NO• chemistry, developed by Lim *et al.* [4], Lancaster [3], and Nalwaya and Deen [9], were individually assessed for their ability to simulate NO• dynamics in a culture of wild-type *E. coli*. The alternative models were constructed and adapted to our experimental system using the following procedure. Starting with the model presented in this study, all reactions absent in the alternative model were eliminated, except the release of NO• from a NONOate, and the NO• and O_2_ liquid-gas transport reactions. Reactions present in the alternative model that were not included in the present model (due to the consumption or production of an unknown or nonspecific species, or the simplification of a more complex process in the present model) were added to the adapted model. For Lancaster's model, the NO• formation and disappearance reactions, as well as the disappearance of NO_2_• and •OH, were not included because the rates of the disappearance reactions are user-defined, and the formation of NO• is accounted for by the NONOate dissociation reaction. The model described by Nalwaya and Deen contains a simplified reaction representing the consumption of NO• by a heme- and flavin-dependent dioxygenase, analogous to Hmp detoxification in *E. coli*. The reaction was included in the adapted model, and the associated bimolecular rate constant was allowed to vary during parameter optimization. Additionally, the rate parameters governing ONOO^−^ and ONOOH reactions used by Nalwaya and Deen were adjusted for a pH of 7.6, where the fraction of ONOO^−^ in protonated form was calculated to be 12% [9]. Although Nalwaya and Deen do not include NO• autoxidation in their model, it was incorporated into the adapted version, as autoxidation is an important effect under the aerobic experimental conditions used in this study.

Species concentrations in the alternative models were set to the same values or ranges used in the present model, except for a few minor differences. The concentrations of proteins and transition metal centers (M^n+^) in the model of Lim *et al.* were allowed a range of 5–8 mM and 1–500 µM, respectively. The protein concentration range was selected based on typical protein content reported for *E. coli* [119], while M^n+^ was allowed the same concentration range as [Fe-S] clusters in the present model, which assumes ∼5% of proteins contain [Fe-S] clusters [120].

The three adapted models were subjected to a parameter optimization procedure analogous to that used for the model presented here (see “Parameter optimization” section above), where parameters classified as uncertain were varied to minimize the SSR between the predicted and experimental [NO•] curves. Ultimately, none of the three adapted models were able to capture the dynamics of NO• measured in wild-type *E. coli* cultures, yielding [NO•] curves with SSR values that were 200-fold (Lim *et al.* and Lancaster) and 70-fold (Nalwaya and Deen) greater than the SSR achieved by the present model (Figure S4).

### Determination of a minimum NO• biochemical reaction network

In order to identify the core set of reactions required to accurately simulate NO• dynamics in aerobic wild-type *E. coli* cultures (Figure 2A), a systematic reduction of the model reaction network was performed using a two-tier process. In the first tier, reactions were sequentially deleted from the original network in a random order. After each reaction deletion, the SSR between the simulated and experimentally-measured [NO•] curve for DPTA-treated wild-type *E. coli* was calculated. If the SSR exceeded a 5% increase over the original SSR, the reaction deletion was undone. This process was repeated until no remaining reactions could be removed without exceeding the 5% increase in SSR. The entire model reduction process was repeated for a total of 100 iterations, each following a random sequence of reaction deletions. The reduced reaction network was selected as the set containing the least number of reactions. In the event of two or more minimum sets, the network yielding the lowest SSR was chosen.

In the second tier, the minimal reaction network was further reduced through a similar reaction deletion process, except with the inclusion of a parameter optimization step. After deleting a reaction, any remaining parameters in the reduced model classified as uncertain (Table S4) were re-optimized, following the nonlinear least-squares optimization procedure described above. If the optimization succeeded in decreasing the SSR to within 5% of the original SSR value, the reaction was removed from the final network. The final, minimum biochemical reaction network determined through this process is presented in Table S5.

## Supporting Information

Figure S1 **Reaction network diagram of nitrosylation and Isc-mediated repair of [Fe-S] clusters.** Shown are the reactions and species incorporated into the model to describe the NO•-mediated nitrosylation and degradation of [2Fe-2S] and [4Fe-4S] clusters, and the repair process carried out by the *Isc* system. In the model, the reductive coupling of the two IscU-bound [2Fe-2S] clusters to form an IscU-bound [4Fe-4S] cluster was combined with the subsequent insertion into an apoprotein (see Materials and methods). Enzymes involved in a reaction are shown above or below the reaction arrow in bolded italics. For simplicity, protons (H^+^) are not shown.
(TIF)

Figure S2 **Reaction network diagram of DNA deamination and base excision repair.** Shown are the model reactions for N_2_O_3_-mediated deamination of DNA bases, and the subsequent process of damaged base removal and repair mediated by the BER system, where N represents DNA base A, G, or C, and N_deam_ is the respective deamination product hX, X, or U. The surrounding DNA strand is simplified and drawn in blue, except for the newly inserted base, which is colored in red to aid in visualization. Enzymes involved in a reaction are shown above or below the reaction arrow in bolded italics. For simplicity, protons are not shown.
(TIF)

Figure S3 **Determination of extracellular parameter values.** Shown are the experimentally-measured and predicted **(A)** NO• concentration curves and **(B)** 10 h NO_2_^−^ and NO_3_^−^ concentrations following addition of 0.5 mM DPTA to cell-free media. Measured values are the mean of 3 independent experiments, with error bars (light green for NO• and black for NO_2_^−^ and NO_3_^−^) representing the standard error of the mean. The extracellular parameters *k*_NONOate_ (NO• donor dissociation), *k*_L_ *a*_NO•_ (NO• transfer to the gas phase), and *k*_NO•-O2_ (NO• autoxidation) were optimized to reproduce the experimentally-measured [NO•] curve and NO_2_^−^ concentration at 10 h post-dose (when it was predicted that over 99% of the DPTA had dissociated). The red line in (B) depicts the NO_3_^−^ assay limit of detection, where the asterisk (*) indicates that the measured NO_3_^−^ concentration was not significantly different from the detection limit (one-sample *t*-test, 95% confidence).
(TIF)

Figure S4 **Comparison of model performance with previous NO• models.** Shown is the NO• concentration (red, with light red error bars representing the standard error of the mean for 3 independent experiments) measured following addition of DPTA (0.5 mM) to a wild-type *E. coli* culture, along with the [NO•] curve predicted (dashed black line) by the present model **(A)**, and the models of **(B)** Lim *et al.* [4], **(C)** Lancaster [3], and **(D)** Nalwaya and Deen [9], which were adapted to our experimental conditions and subjected to an analogous parameter optimization procedure (see Materials and Methods).
(TIF)

Figure S5 **Sensitivity analysis of uncertain model parameters under aerobic conditions.** **(A)** Effect of varying uncertain parameters on NO• dynamics. Each uncertain parameter was varied among 5 equally-spaced values spanning its range (Table S4), and the corresponding NO• concentration profile was calculated. NO• concentration profiles resulting from varying the 35 of the 39 uncertain parameters that did not show an appreciable change in the sum of squared residuals (SSR) between the predicted and experimentally-measured NO• concentration profile (aerobic, wild-type treated with DPTA) upon variation are shown (for a total of 35×5 = 175 curves plotted). The inset shows a zoomed region of the curve, to illustrate the minor effect of varying these parameters. **(B)** For comparison, the NO• concentration profiles obtained when varying the maximum Hmp expression rate parameter (*k*_Hmp-exp,max_) are shown (red lines).
(TIF)

Figure S6 **Effect of Δ** ***norV*** **on NO• dynamics in aerobic** ***E. coli*** **cultures.** Shown are the measured and predicted NO• concentrations measured following the addition of 0.5 mM DPTA to **(A)** cell-free growth media, **(B)** wild-type *E. coli* culture, and **(C)** Δ*norV E. coli* culture. Error bars (light green, light red, and light purple for media, wild-type, and Δ*norV*, respectively) represent the standard error of the mean for 3 independent experiments. We note that these measurements were obtained with a separate ISO-NOP NO• sensor than the one used to generate Figure 2A, as a result of their limited lifetime. Due to minor probe-to-probe variations, the cell-free and wild-type NO• curves were re-measured and the model parameters re-optimized to generate the predictions shown.
(TIF)

Figure S7 **Determination of PAPA NO• release rate.** Shown is the NO• concentration following addition of 0.5 mM PAPA to MOPS glucose media measured experimentally (solid green line) or predicted by the model (dashed black line) after optimizing the NO• donor dissociation rate parameter, *k*_NONOate_, to reproduce experimental [NO•] curve. Experimental measurements were performed under identical conditions to those of the NO• consumption assays (Materials and methods), except there were no cells present. The release rate was calculated to be 1.35×10^−3^ s^−1^ (8.6 min half-life).
(TIF)

Figure S8 **Measured O_2_ concentration during microaerobic NO• consumption assays.** Shown are the dissolved O_2_ concentration profiles of the culture (average of at least 3 independent experiments for each curve) measured following addition of DPTA to wild-type, Δ*norV*, or Δ*hmp E. coli* cultures during N_2_ bubbling, which remained constant at approximately 35 µM (∼19% air saturation). Error bars (light red, light purple, and light blue for wild-type, Δ*norV*, and Δ*hmp*, respectively) represent the standard error of the mean. For comparison, the dashed black line depicts the O_2_ concentration of air-saturated growth media at 37°C in the absence of N_2_ bubbling (185 µM).
(TIF)

Figure S9 **Parametric analysis under microaerobic conditions.** Fold increase in the SSR between the experimentally measured and predicted NO• concentration (wild-type *E. coli*, treated with 0.5 mM DPTA) under microaerobic (35 µM O_2_) conditions is plotted as a function of parameter value for the 8 of 42 optimized parameters (Table S7) exhibiting a greater than 5% increase in the SSR upon variation. Fold increases colored in green, blue, and orange represent extracellular, Hmp-, and NorV-associated parameters, respectively. The remaining 34 parameters exhibited a negligible effect on the SSR.
(TIF)

Figure S10 **Effect of individual parameter variation on the predicted NO• distribution under aerobic conditions.** Shown are 175 vertical bars representing the predicted final (t→∞) distributions of **(A)** intracellular and **(B)** total NO• consumption after treatment with 0.5 mM DPTA for each parameter set during parametric analysis. Parameter sets were generated by varying each of the 35 uncertain parameters found to have negligible influence on the NO• concentration profile among 5 logarithmically-spaced values spanning their allowed range (Table S4). Parameter sets are sorted from left to right by increasing fraction of intracellular NO• consumed by Hmp. The intracellular distributions are re-plotted with a zoomed *y*-axis on the right to show the pathways with contributions too small to see on the full scale. “Hmp” is detoxification of NO• by Hmp, “Oxidation” is NO• consumed through reaction with O_2_ or O_2_•^−^, “NorV/NrfA” is the reduction of NO• by NorV or NrfA, and “[Fe-S]” is NO• consumed by the nitrosylation of iron-sulfur clusters. “Cellular” refers to NO• consumed by any intracellular pathway, “Gas” is loss of NO• to the gas phase, and “Autoxidation” is reaction of NO• with O_2_ in the media.
(TIF)

Figure S11 **Effect of combinatorial parameter variation on the predicted NO• distribution under aerobic conditions.** Shown are 100,000 vertical bars representing the predicted final (t→∞) distributions of **(A)** intracellular and **(B)** total NO• consumption after treatment with 0.5 mM DPTA calculated for each parameter set during randomized combinatorial parametric analysis. The plots are similar to those in Figure S10, except the 100,000 parameter sets were generated by assigning each of the 35 uncertain parameters to a random value within their allowed range (Table S4).
(TIF)

Figure S12 **Measurement of O_2_ volumetric mass transfer coefficient (** ***k*** **_L_** ***a*** **_O2_).** **(A)** The concentration of O_2_ was measured in stirred MOPS glucose media at 37°C in contact with air after degassing with N_2_. **(B)** The O_2_ concentration data was re-plotted as ln([O_2_]_sat_ – [O_2_]) vs. time (black line) to calculate the value of *k*_L_ *a*_O2_ (see detailed description of calculation in Text S1). A line (red) was fit to the data, where the negative of the slope (4.92 h^−1^, or 1.37×10^−3^ s^−1^) corresponds to the *k*_L_ *a*_O2_.
(TIF)

Figure S13 **Measurement of O_2_ concentration prior to NONOate addition.** The dissolved O_2_ concentration in an aerobic wild-type *E. coli* culture was measured during the period of growth prior to addition of NONOate. Conditions were identical to those used for the aerobic NO• consumption assays (Materials and Methods). O_2_ concentration was found to steadily decrease to approximately 130 µM due to cellular respiration before it reached an OD_600_ of 0.05, at which point the NONOate was added.
(TIF)

Figure S14 **Estimation of the rate constant governing cysteine-mediated removal of protein-bound DNIC.** Experimental EPR data tracking the cysteine-mediated removal of DNICs from proteins was obtained from literature [94] (black triangles), and used to approximate the associated rate constant by minimizing the SSR between prediction and experiment. The predicted curve obtained using the optimized *k*_DNIC-rem_ value is shown (dashed red line) (see Text S1 for further detail).
(TIF)

Table S1 **Biochemical species included in the model.** All metabolites, enzymes, and biomolecules are listed with their initial concentrations.
(XLSX)

Table S2 **Model reactions governed by elementary-type rate expressions.** Reactions are listed along with the value of their associated rate constant.
(XLSX)

Table S3 **Model reactions with complex rate expressions.** Reactions are listed with their associated rate expression and rate constants. Reaction numbering is continued from Table S2. Asterisks (*) denote uncertain parameter values that were varied during parametric analysis and optimization.
(PDF)

Table S4 **Uncertain model parameters.** “Reaction #s” are the numbers of the reactions governed by the rate parameter, and correspond to the numbering in Tables S2, S3, and Text S1. Allowed parameter ranges (defined by “Min.” and “Max.”) were chosen to encompass the value(s) obtained or calculated from literature, unless otherwise noted. “Optimal” are the parameter values from the optimization yielding the lowest SSR between the predicted and experimentally-measured [NO•] curve for wild-type *E. coli* treated with DPTA under aerobic conditions. Confidence intervals (C.I.) are provided for parameters that were informed by the optimization, and were calculated as the range of optimal parameter values obtained for the top 10% of optimization outcomes (those with the lowest SSR values).
(PDF)

Table S5 **Minimum biochemical reaction network necessary to simulate NO• dynamics in aerobic, wild-type** ***E. coli*** **cultures.** **(A) Reactions.** Reaction numbers correspond to those used in Tables S2, S3, and Text S1. **(B) Biochemical Species.** Species numbers and initial concentrations (M) correspond to those reported in Table S1. **(C) Kinetic parameters.** “Reaction #s” are the numbers of the reactions governed by the rate parameter, and correspond to the numbering in Tables S2, S3, and Text S1.
(PDF)

Table S6 **Experimentally-accessible model parameters varied during parametric analysis.** “Reaction #s” are the numbers of the reactions governed by the rate parameter, and correspond to the numbering in Tables S2, S3. Model parameters were varied among 5 logarithmically-spaced values spanning their allowed range. Allowed parameter ranges (defined by “Min.” and “Max.”) were chosen to span the value obtained or calculated from literature, unless otherwise noted.
(PDF)

Table S7 **Model parameters optimized for microaerobic conditions.** “Reaction #s” are the numbers of the reactions governed by the rate parameter, and correspond to the numbering in Tables S2, S3, and Text S1. Allowed parameter ranges (defined by “Min.” and “Max.”) were chosen to encompass the value(s) obtained or calculated from literature, unless otherwise noted. “Optimal” are the parameter values from the optimization yielding the lowest SSR between the predicted and experimentally-measured [NO•] curve for wild-type *E. coli* treated with DPTA under microaerobic (35 µM O_2_) conditions. Confidence intervals (C.I.) are provided for parameters that were informed by the optimization, and were calculated as the range of optimal parameter values obtained for the top 10% of optimization outcomes (those with the lowest SSR values).
(PDF)

Text S1 **Additional details on model development and analysis.** Detailed model description, parametric analysis of model parameters with uncertain values, measurement of extracellular NO• kinetic parameters, measurement of O_2_ volumetric mass transfer coefficient (*k*_L_ *a*_O2_), determination of O_2_ concentration prior to NO• stress, Hmp reaction mechanism and kinetics, O_2_-mediated inactivation of NorV, enzyme expression and degradation, and protein-bound DNIC removal and degradation.
(PDF)
