# Supplementary figures and images for: A Kinetic Platform to Determine the Fate of Nitric Oxide in Escherichia coli
Source: PLoS Comput Biol. 2013 May 2;9(5):e1003049. doi: 10.1371/journal.pcbi.1003049 (PMC3642044; doi:10.1371/journal.pcbi.1003049)

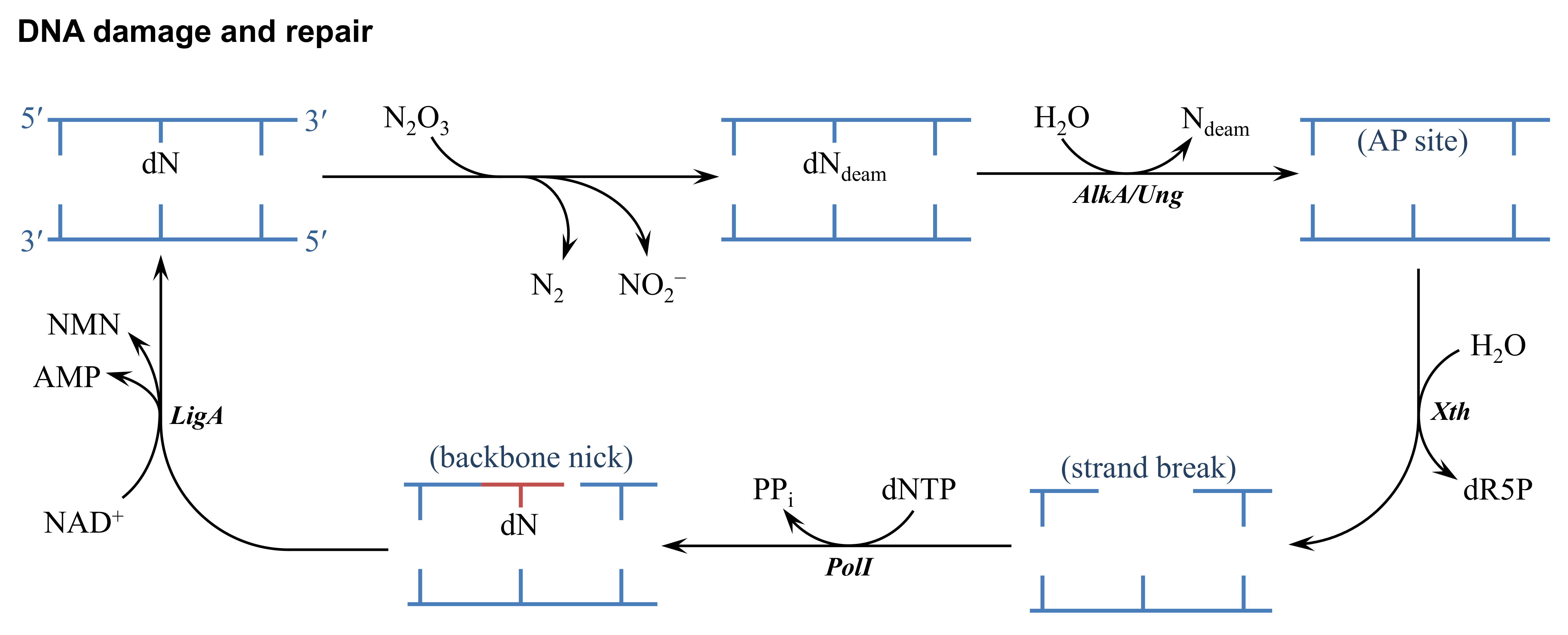

Supplement: Figure S2 — Reaction network diagram of DNA deamination and base excision repair. Shown are the model reactions for N2O3-mediated deamination of DNA bases, and the subsequent process of damaged base removal and repair mediated by the BER system, where N represents DNA base A, G, or C, and Ndeam is the respective deamination product hX, X, or U. The surrounding DNA strand is simplified and drawn in blue, except for the newly inserted base, which is colored in red to aid in visualization. Enzymes involved in a reaction are shown above or below the reaction arrow in bolded italics. For simplicity, protons are not shown. (TIF) [file pcbi.1003049.s002.tif]

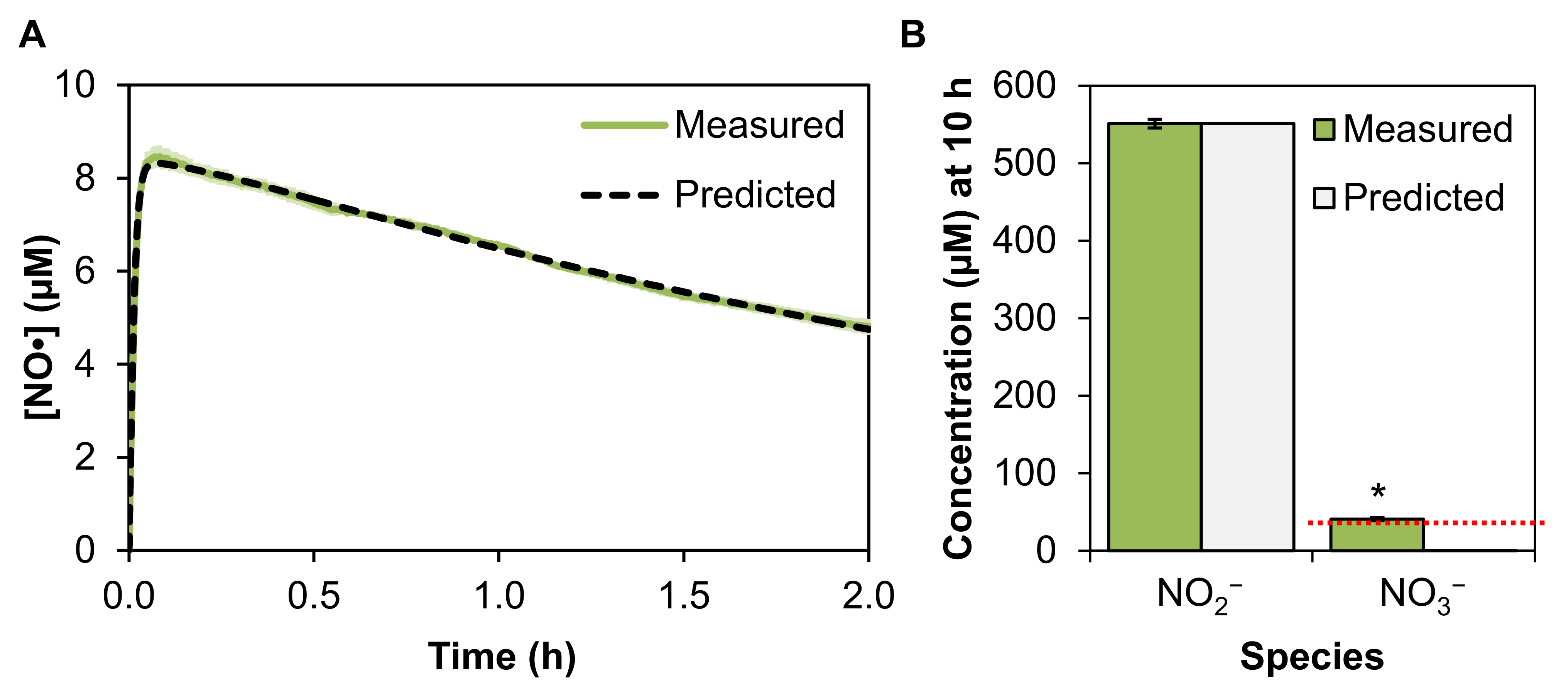

Supplement: Figure S3 — Determination of extracellular parameter values. Shown are the experimentally-measured and predicted (A) NO• concentration curves and (B) 10 h NO2 − and NO3 − concentrations following addition of 0.5 mM DPTA to cell-free media. Measured values are the mean of 3 independent experiments, with error bars (light green for NO• and black for NO2 − and NO3 −) representing the standard error of the mean. The extracellular parameters k NONOate (NO• donor dissociation), k L a NO• (NO• transfer to the gas phase), and k NO•-O2 (NO• autoxidation) were optimized to reproduce the experimentally-measured [NO•] curve and NO2 − concentration at 10 h post-dose (when it was predicted that over 99% of the DPTA had dissociated). The red line in (B) depicts the NO3 − assay limit of detection, where the asterisk (*) indicates that the measured NO3 − concentration was not significantly different from the detection limit (one-sample t-test, 95% confidence). (TIF) [file pcbi.1003049.s003.tif]

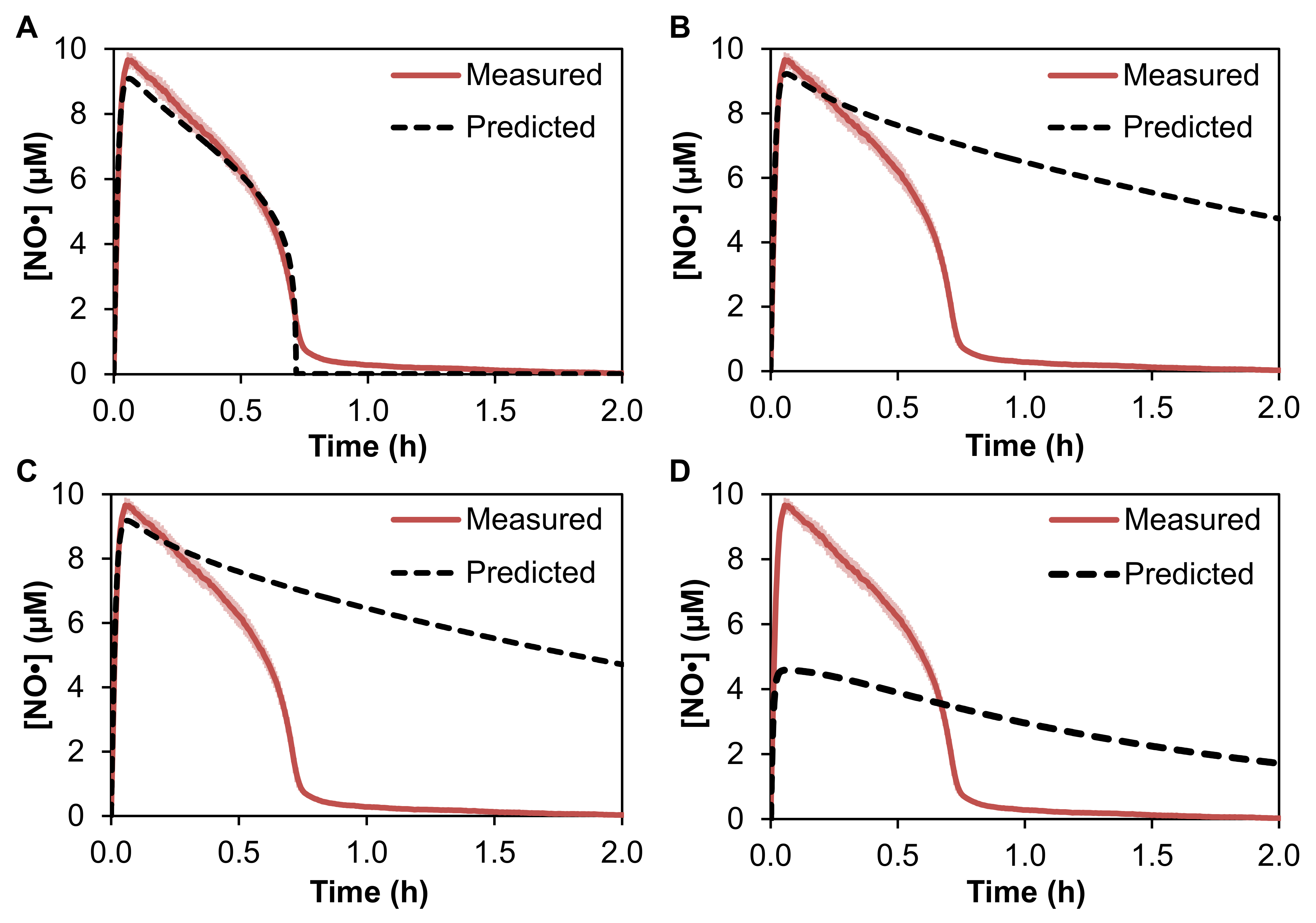

Supplement: Figure S4 — Comparison of model performance with previous NO• models. Shown is the NO• concentration (red, with light red error bars representing the standard error of the mean for 3 independent experiments) measured following addition of DPTA (0.5 mM) to a wild-type E. coli culture, along with the [NO•] curve predicted (dashed black line) by the present model (A), and the models of (B) Lim et al. [4], (C) Lancaster [3], and (D) Nalwaya and Deen [9], which were adapted to our experimental conditions and subjected to an analogous parameter optimization procedure (see Materials and Methods). (TIF) [file pcbi.1003049.s004.tif]

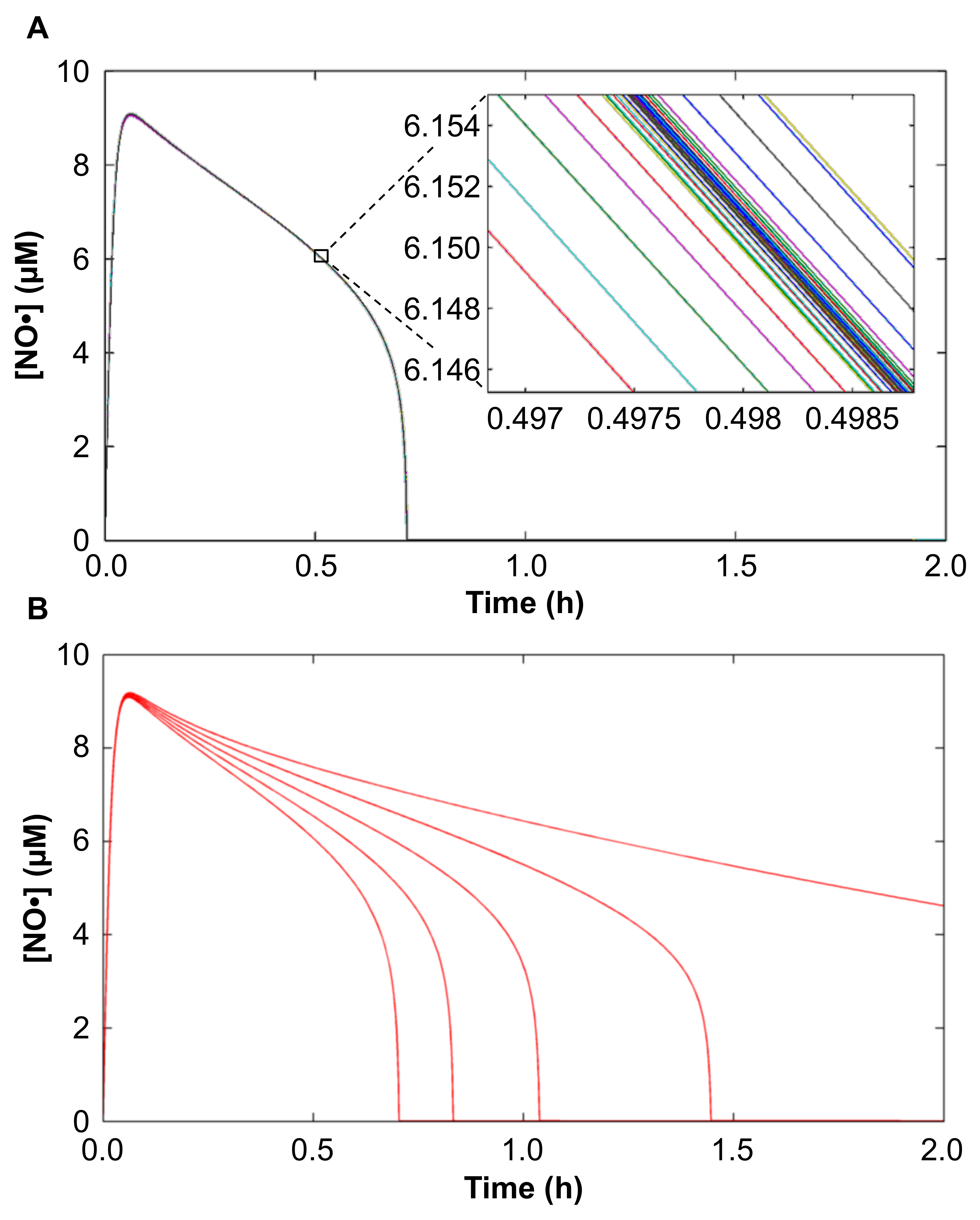

Supplement: Figure S5 — Sensitivity analysis of uncertain model parameters under aerobic conditions. (A) Effect of varying uncertain parameters on NO• dynamics. Each uncertain parameter was varied among 5 equally-spaced values spanning its range (Table S4), and the corresponding NO• concentration profile was calculated. NO• concentration profiles resulting from varying the 35 of the 39 uncertain parameters that did not show an appreciable change in the sum of squared residuals (SSR) between the predicted and experimentally-measured NO• concentration profile (aerobic, wild-type treated with DPTA) upon variation are shown (for a total of 35×5 = 175 curves plotted). The inset shows a zoomed region of the curve, to illustrate the minor effect of varying these parameters. (B) For comparison, the NO• concentration profiles obtained when varying the maximum Hmp expression rate parameter (k Hmp-exp,max) are shown (red lines). (TIF) [file pcbi.1003049.s005.tif]

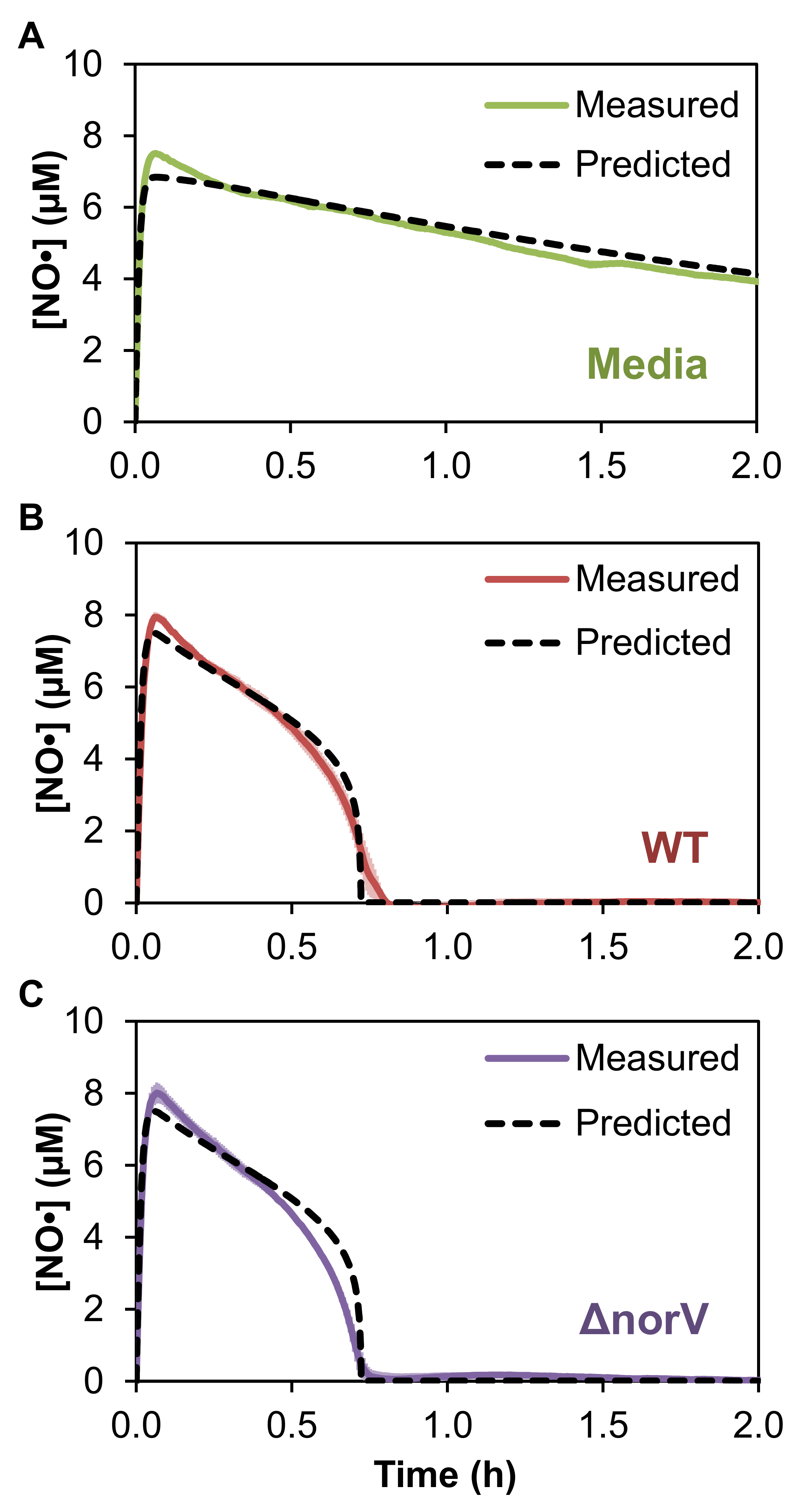

Supplement: Figure S6 — Effect of Δ norV on NO• dynamics in aerobic E. coli cultures. Shown are the measured and predicted NO• concentrations measured following the addition of 0.5 mM DPTA to (A) cell-free growth media, (B) wild-type E. coli culture, and (C) ΔnorV E. coli culture. Error bars (light green, light red, and light purple for media, wild-type, and ΔnorV, respectively) represent the standard error of the mean for 3 independent experiments. We note that these measurements were obtained with a separate ISO-NOP NO• sensor than the one used to generate Figure 2A, as a result of their limited lifetime. Due to minor probe-to-probe variations, the cell-free and wild-type NO• curves were re-measured and the model parameters re-optimized to generate the predictions shown. (TIF) [file pcbi.1003049.s006.tif]

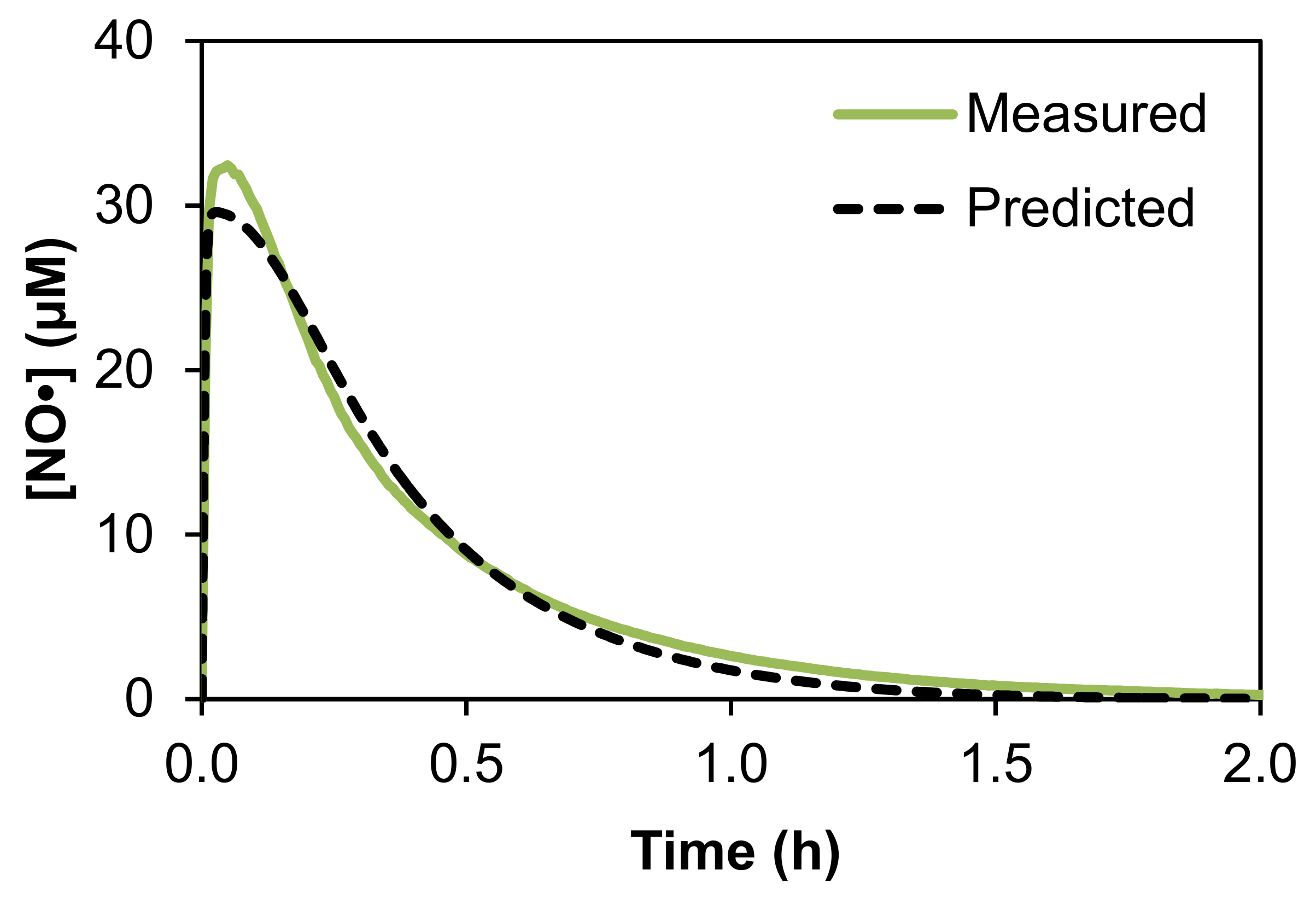

Supplement: Figure S7 — Determination of PAPA NO• release rate. Shown is the NO• concentration following addition of 0.5 mM PAPA to MOPS glucose media measured experimentally (solid green line) or predicted by the model (dashed black line) after optimizing the NO• donor dissociation rate parameter, k NONOate, to reproduce experimental [NO•] curve. Experimental measurements were performed under identical conditions to those of the NO• consumption assays (Materials and methods), except there were no cells present. The release rate was calculated to be 1.35×10−3 s−1 (8.6 min half-life). (TIF) [file pcbi.1003049.s007.tif]

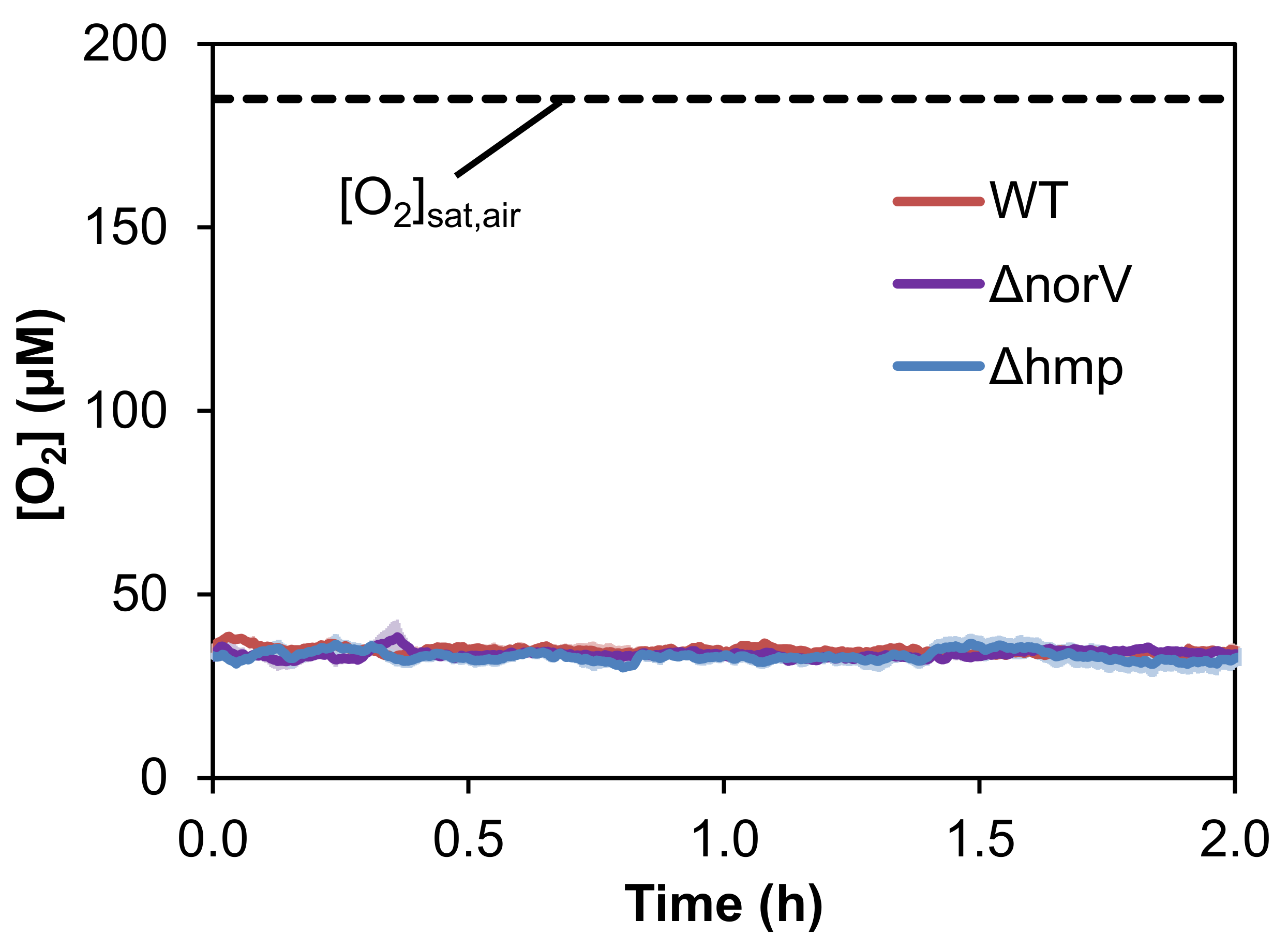

Supplement: Figure S8 — Measured O2 concentration during microaerobic NO• consumption assays. Shown are the dissolved O2 concentration profiles of the culture (average of at least 3 independent experiments for each curve) measured following addition of DPTA to wild-type, ΔnorV, or Δhmp E. coli cultures during N2 bubbling, which remained constant at approximately 35 µM (∼19% air saturation). Error bars (light red, light purple, and light blue for wild-type, ΔnorV, and Δhmp, respectively) represent the standard error of the mean. For comparison, the dashed black line depicts the O2 concentration of air-saturated growth media at 37°C in the absence of N2 bubbling (185 µM). (TIF) [file pcbi.1003049.s008.tif]

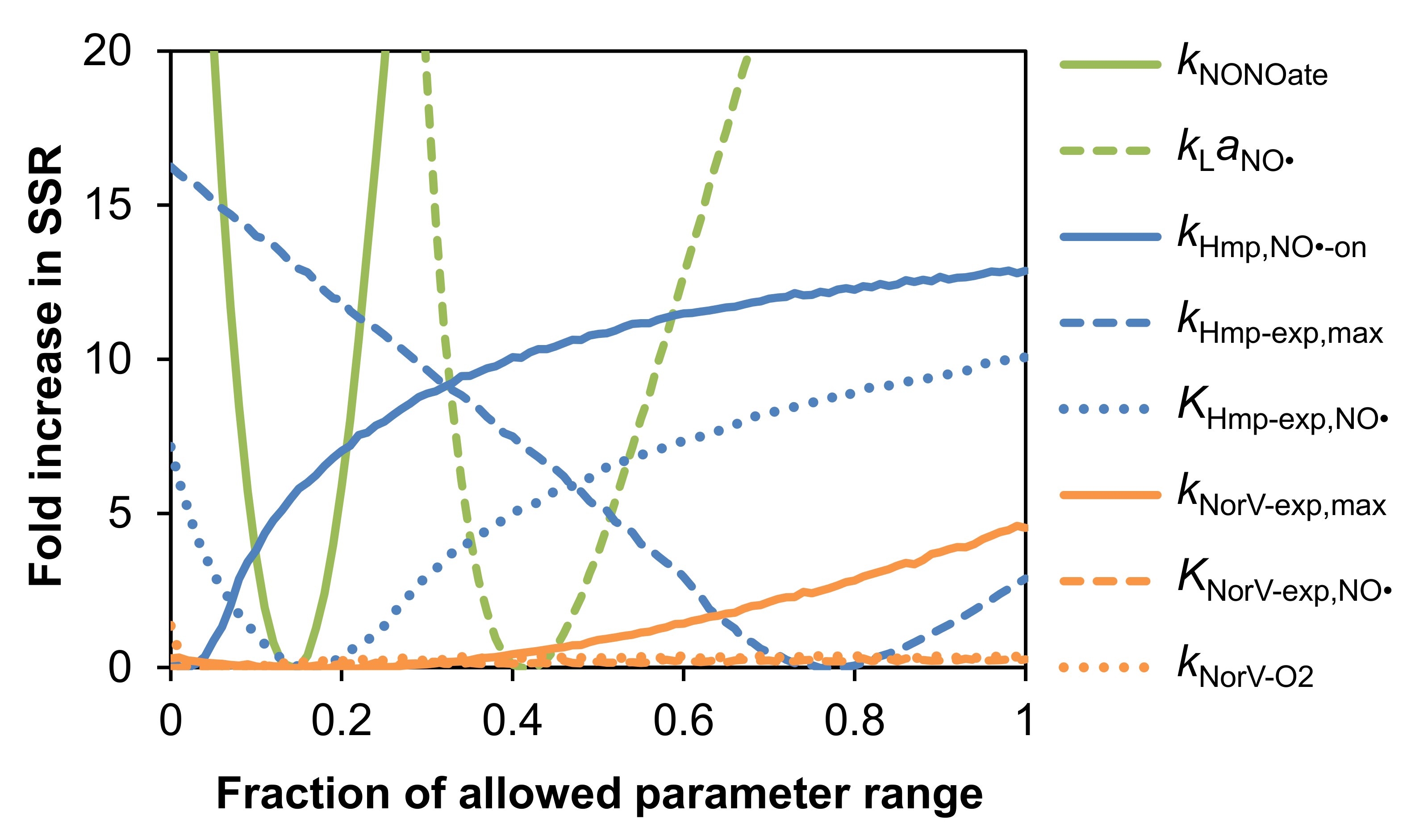

Supplement: Figure S9 — Parametric analysis under microaerobic conditions. Fold increase in the SSR between the experimentally measured and predicted NO• concentration (wild-type E. coli, treated with 0.5 mM DPTA) under microaerobic (35 µM O2) conditions is plotted as a function of parameter value for the 8 of 42 optimized parameters (Table S7) exhibiting a greater than 5% increase in the SSR upon variation. Fold increases colored in green, blue, and orange represent extracellular, Hmp-, and NorV-associated parameters, respectively. The remaining 34 parameters exhibited a negligible effect on the SSR. (TIF) [file pcbi.1003049.s009.tif]

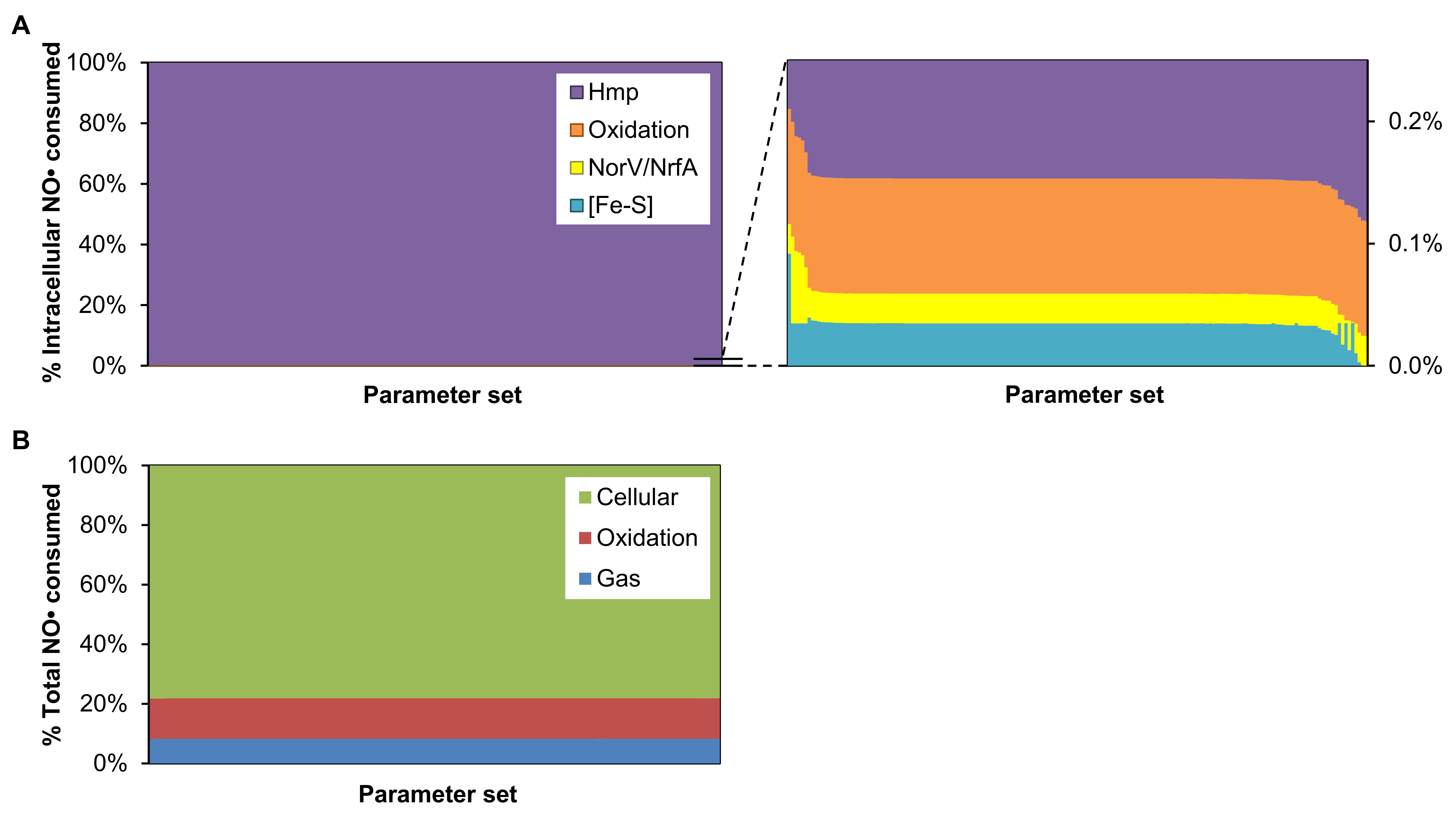

Supplement: Figure S10 — Effect of individual parameter variation on the predicted NO• distribution under aerobic conditions. Shown are 175 vertical bars representing the predicted final (t→∞) distributions of (A) intracellular and (B) total NO• consumption after treatment with 0.5 mM DPTA for each parameter set during parametric analysis. Parameter sets were generated by varying each of the 35 uncertain parameters found to have negligible influence on the NO• concentration profile among 5 logarithmically-spaced values spanning their allowed range (Table S4). Parameter sets are sorted from left to right by increasing fraction of intracellular NO• consumed by Hmp. The intracellular distributions are re-plotted with a zoomed y-axis on the right to show the pathways with contributions too small to see on the full scale. “Hmp” is detoxification of NO• by Hmp, “Oxidation” is NO• consumed through reaction with O2 or O2•−, “NorV/NrfA” is the reduction of NO• by NorV or NrfA, and “[Fe-S]” is NO• consumed by the nitrosylation of iron-sulfur clusters. “Cellular” refers to NO• consumed by any intracellular pathway, “Gas” is loss of NO• to the gas phase, and “Autoxidation” is reaction of NO• with O2 in the media. (TIF) [file pcbi.1003049.s010.tif]

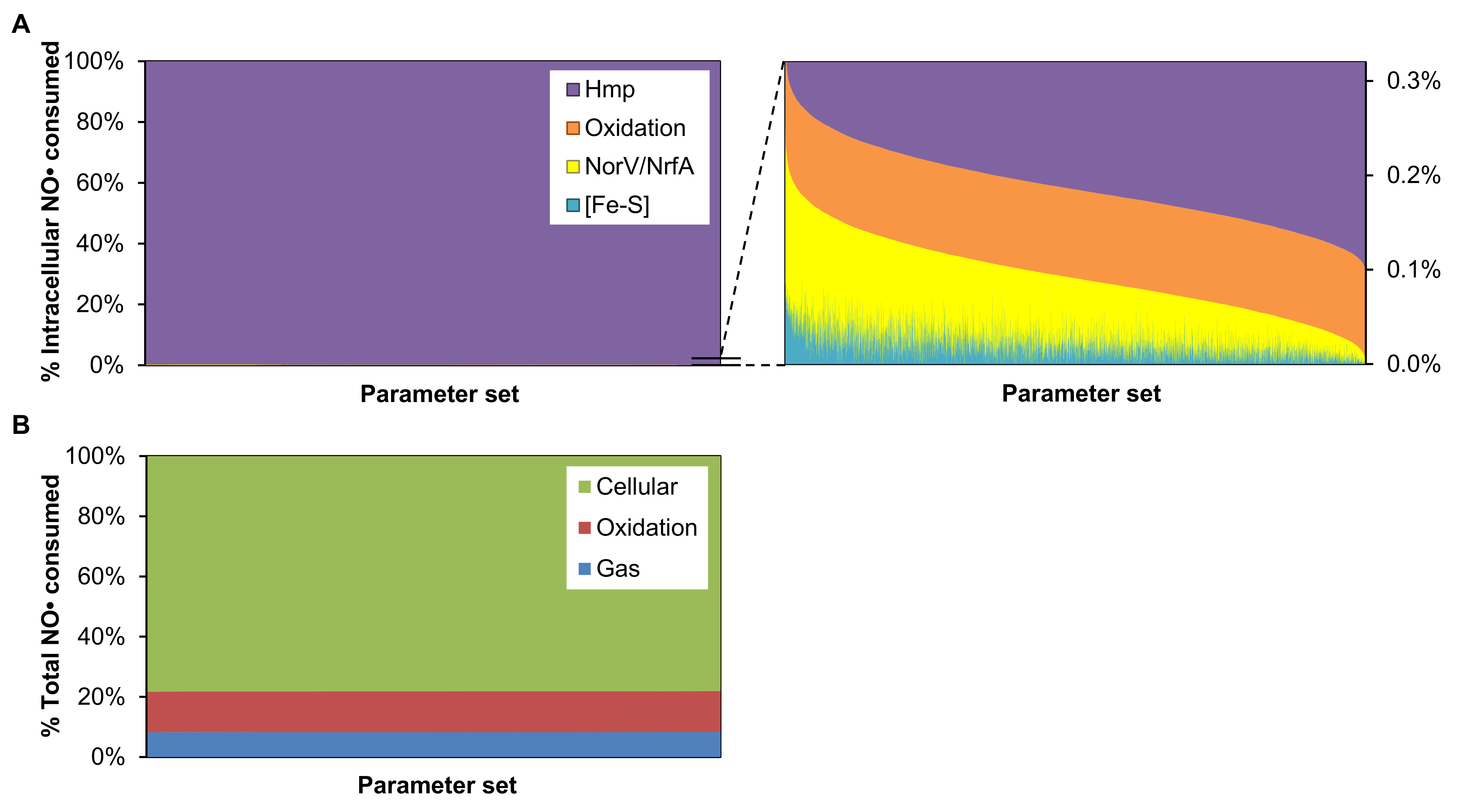

Supplement: Figure S11 — Effect of combinatorial parameter variation on the predicted NO• distribution under aerobic conditions. Shown are 100,000 vertical bars representing the predicted final (t→∞) distributions of (A) intracellular and (B) total NO• consumption after treatment with 0.5 mM DPTA calculated for each parameter set during randomized combinatorial parametric analysis. The plots are similar to those in Figure S10, except the 100,000 parameter sets were generated by assigning each of the 35 uncertain parameters to a random value within their allowed range (Table S4). (TIF) [file pcbi.1003049.s011.tif]

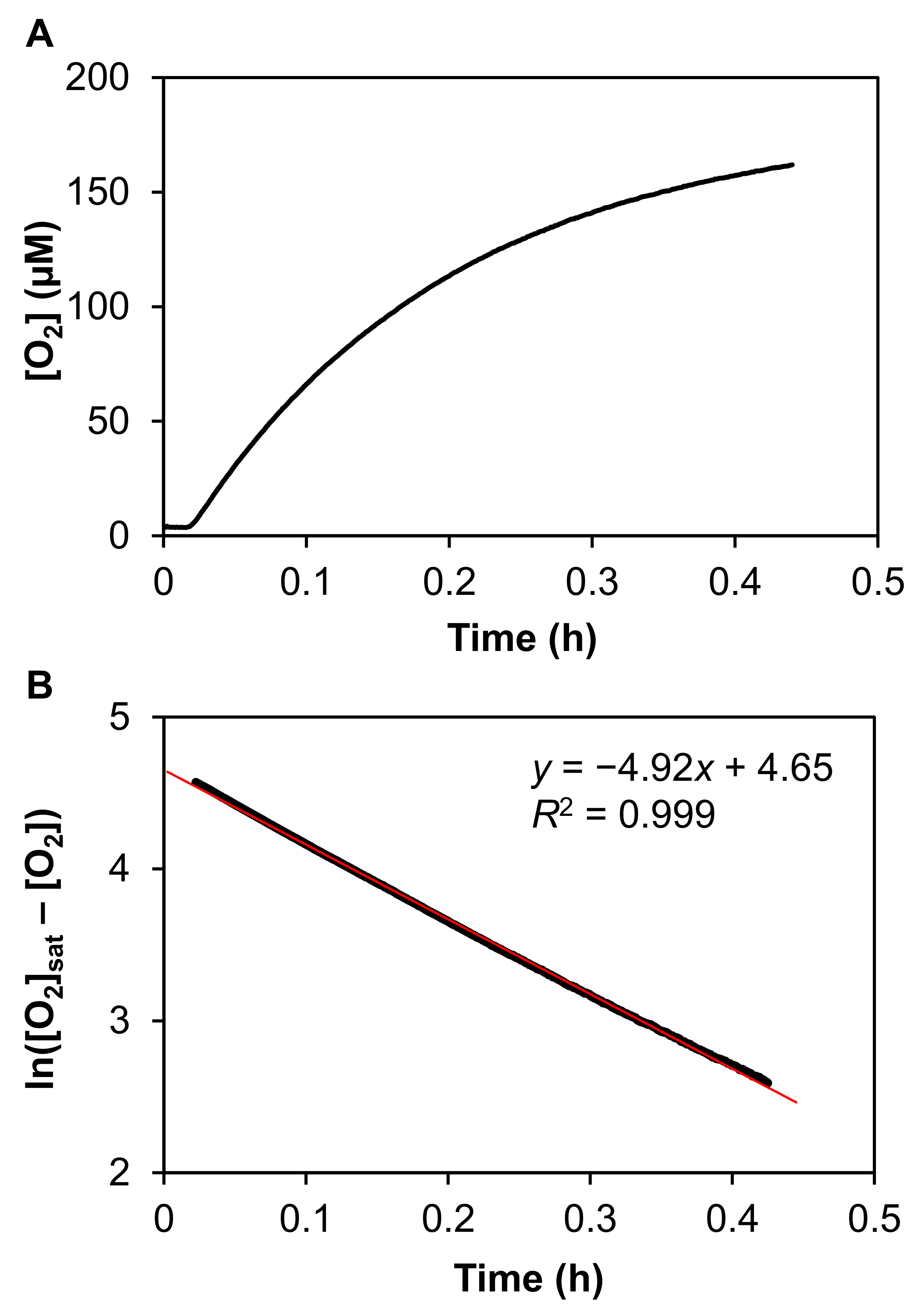

Supplement: Figure S12 — Measurement of O2 volumetric mass transfer coefficient ( k L a O2). (A) The concentration of O2 was measured in stirred MOPS glucose media at 37°C in contact with air after degassing with N2. (B) The O2 concentration data was re-plotted as ln([O2]sat – [O2]) vs. time (black line) to calculate the value of k L a O2 (see detailed description of calculation in Text S1). A line (red) was fit to the data, where the negative of the slope (4.92 h−1, or 1.37×10−3 s−1) corresponds to the k L a O2. (TIF) [file pcbi.1003049.s012.tif]

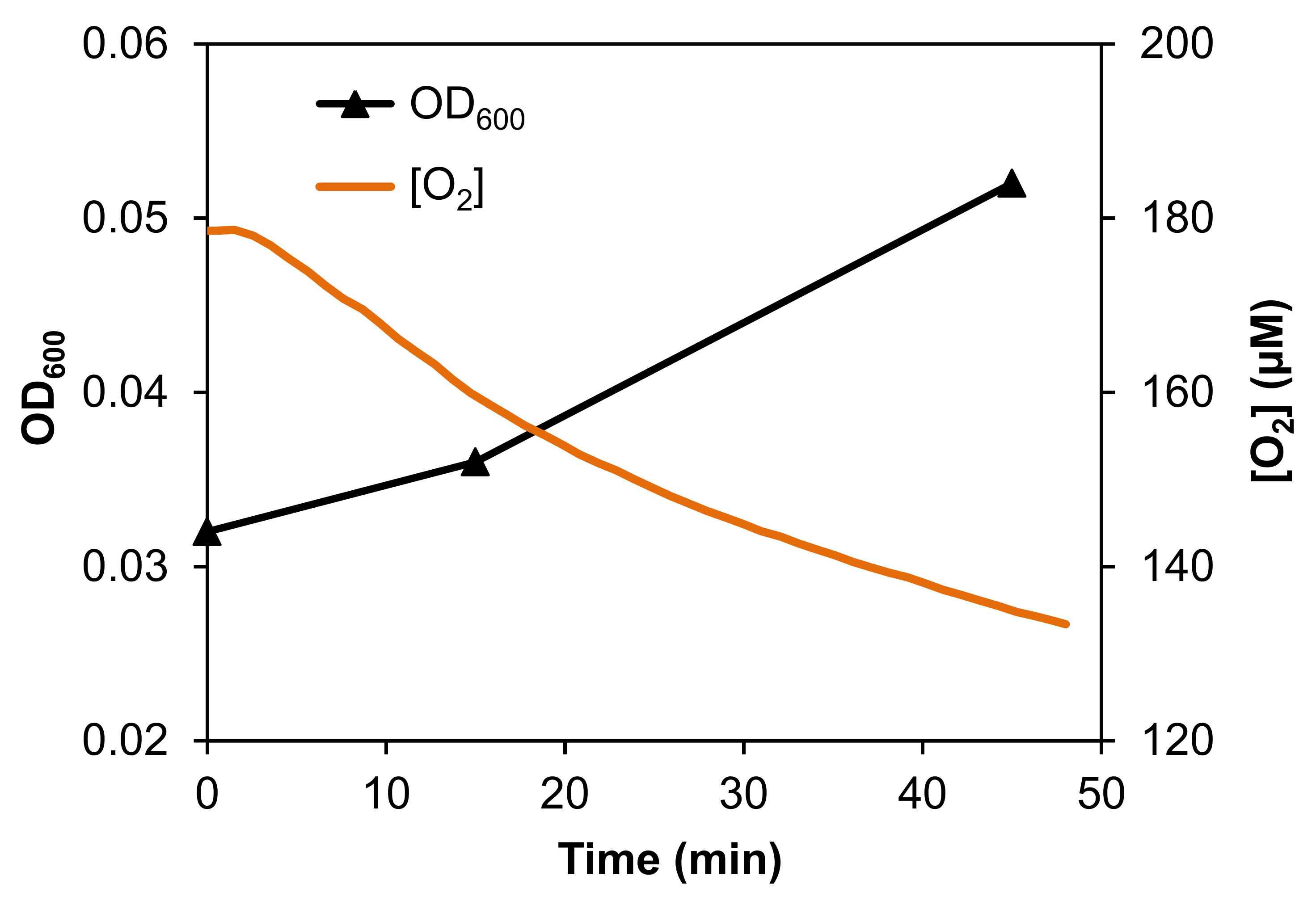

Supplement: Figure S13 — Measurement of O2 concentration prior to NONOate addition. The dissolved O2 concentration in an aerobic wild-type E. coli culture was measured during the period of growth prior to addition of NONOate. Conditions were identical to those used for the aerobic NO• consumption assays (Materials and Methods). O2 concentration was found to steadily decrease to approximately 130 µM due to cellular respiration before it reached an OD600 of 0.05, at which point the NONOate was added. (TIF) [file pcbi.1003049.s013.tif]

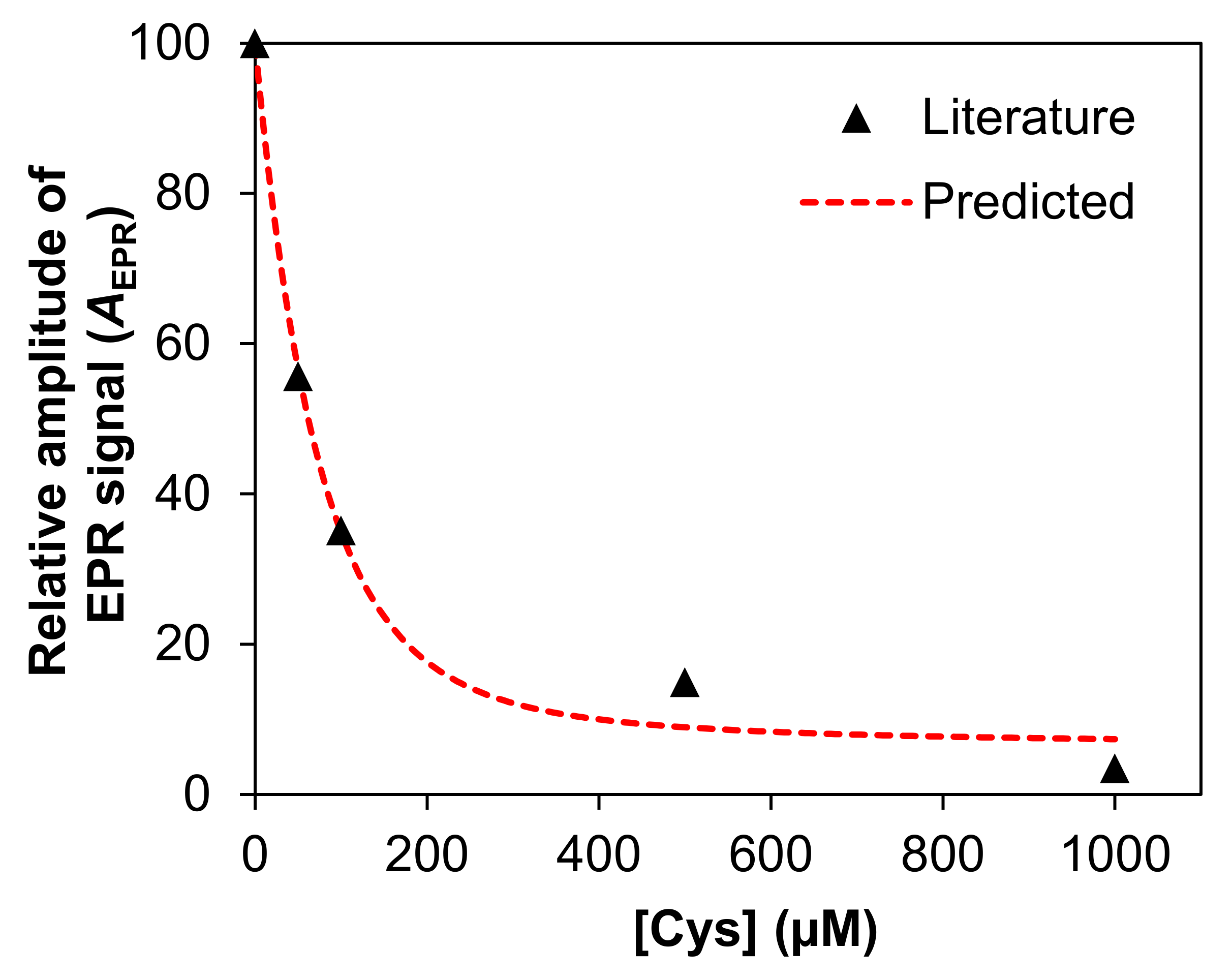

Supplement: Figure S14 — Estimation of the rate constant governing cysteine-mediated removal of protein-bound DNIC. Experimental EPR data tracking the cysteine-mediated removal of DNICs from proteins was obtained from literature [94] (black triangles), and used to approximate the associated rate constant by minimizing the SSR between prediction and experiment. The predicted curve obtained using the optimized k DNIC-rem value is shown (dashed red line) (see Text S1 for further detail). (TIF) [file pcbi.1003049.s014.tif]
