# Supplementary material for: A Kinetic Platform to Determine the Fate of Nitric Oxide in Escherichia coli
Source: PLoS Comput Biol. 2013 May 2;9(5):e1003049. doi: 10.1371/journal.pcbi.1003049 (PMC3642044; doi:10.1371/journal.pcbi.1003049)
Supplement: Table S3 — Model reactions with complex rate expressions. Reactions are listed with their associated rate expression and rate constants. Reaction numbering is continued from Table S2. Asterisks (*) denote uncertain parameter values that were varied during parametric analysis and optimization. (PDF) [file pcbi.1003049.s017.pdf]

Table S3. Model reactions with complex rate expressions. Reactions are listed with their associated rate expression and rate constants. Reaction numbering is continued from Table S2. Asterisks (*) denote uncertain parameter values that were varied during parametric analysis and optimization.

#	Reaction and kinetic expression	Parameters	References
151	$\mathrm{IscU + 2\ Cys \xrightarrow{IscS} IscU(2S)^{2-} + 2\ Ala + 2\ H^+}$<br>$r = \dfrac{k_{cat}[\mathrm{IscS}][\mathrm{IscU}][\mathrm{Cys}]}{[\mathrm{IscU}][\mathrm{Cys}] + K_{\mathrm{Cys}}[\mathrm{IscU}] + K_{\mathrm{IscU}}[\mathrm{Cys}]}$	$k_{cat} = 0.07\ s^{-1}$<br>$K_{Cys} = 2.7\times10^{-6}$ M *<br>$K_{IscU} = 2.6\times10^{-6}$ M *	[1][a]<br>[1]<br>[1,2]
152	$\mathrm{IscU([2Fe\text{-}2S])^{2-} + 2\ Cys \xrightarrow{IscS} IscU([2Fe\text{-}2S]\text{-}2S)^{4-} + Ala + 2\ H^+}$<br>$r = \dfrac{k_{cat}[\mathrm{IscS}][\mathrm{IscU(2Fe2S)}][\mathrm{Cys}]}{[\mathrm{IscU(2Fe2S)}][\mathrm{Cys}] + K_{\mathrm{Cys}}[\mathrm{IscU(2Fe2S)}] + K_{\mathrm{IscU(2Fe2S)}}[\mathrm{Cys}]}$	$k_{cat} = 0.07\ s^{-1}$<br>$K_{Cys} = 2.7\times10^{-6}$ M *<br>$K_{IscU} = 2.6\times10^{-6}$ M *	[1][a]<br>[1]<br>[1,2]
153	$P_{\mathrm{2Fe2S}}(apo) + \mathrm{IscU([2Fe\text{-}2S])^{2-} + ATP + H_2O \xrightarrow{HscAB} IscU} + P_{\mathrm{2Fe2S}}(holo) + \mathrm{ADP + P_i + H^+}$<br>$r = \dfrac{k_{cat}[\mathrm{IscU(2Fe2S)}][P_{\mathrm{2Fe2S}}(apo)]}{K_{P_{\mathrm{2Fe2S}}(apo)} + [P_{\mathrm{2Fe2S}}(apo)]}$	$k_{cat} = 3.5\times10^{-3}\ s^{-1}$ *<br>$K_{P2Fe2S(apo)} = 2.7\times10^{-5}$ M *	[3][b]<br>[3]
154	$P_{\mathrm{2Fe2S}}(apo) + \mathrm{IscU([2Fe\text{-}2S]_2)^{4-} + ATP + H_2O \xrightarrow{HscAB} IscU([2Fe\text{-}2S])} + P_{\mathrm{2Fe2S}}(holo) + \mathrm{ADP + P_i + H^+}$<br>$r = \dfrac{k_{cat}[\mathrm{IscU(2Fe2S)_2}][P_{\mathrm{2Fe2S}}(apo)]}{K_{P_{\mathrm{2Fe2S}}(apo)} + [P_{\mathrm{2Fe2S}}(apo)]}$	$k_{cat} = 3.5\times10^{-3}\ s^{-1}$ *<br>$K_{P2Fe2S(apo)} = 2.7\times10^{-5}$ M *	[3][b,c]<br>[3]
155	$\mathrm{DNA(dX) + H_2O \xrightarrow{AlkA} DNA(AP_G) + X}$<br>$r = \dfrac{k_{cat}[\mathrm{AlkA}][\mathrm{DNA(dX)}]}{K_{\mathrm{DNA(dX)}} + [\mathrm{DNA(dX)}]}$	$k_{cat} = 4.0\times10^{-4}\ s^{-1}$<br>$K_{DNA(dX)} = 5.3\times10^{-8}$ M *	[4][d]<br>[4]
156	$\mathrm{DNA(dI) + H_2O \xrightarrow{AlkA} DNA(AP_A) + hX}$<br>$r = \dfrac{k_{cat}[\mathrm{AlkA}][\mathrm{DNA(dI)}]}{K_{\mathrm{DNA(dI)}} + [\mathrm{DNA(dI)}]}$	$k_{cat} = 1.3\times10^{-3}\ s^{-1}$<br>$K_{DNA(dI)} = 4.2\times10^{-8}$ M *	[5]<br>[5]

157	$\mathrm{DNA(dU)} + \mathrm{H_2O} \xrightarrow{Ung} \mathrm{DNA(AP_C)} + \mathrm{U}$<br>$r = \dfrac{k_{\mathrm{cat}}[\mathrm{Ung}][\mathrm{DNA(dU)}]}{K_{\mathrm{DNA(dU)}} + [\mathrm{DNA(dU)}]}$	$k_{\mathrm{cat}} = 0.5\ \mathrm{s}^{-1}$<br>$K_{\mathrm{DNA(dU)}} = 2.78\times10^{-7}$ M *	[6]<br>[6,7][e]
158	$\mathrm{DNA(AP_G)} + 2\ \mathrm{H_2O} \xrightarrow{Xth} \mathrm{DNA(dG)_{gap}} + 2\ \mathrm{H^+} + \mathrm{dR5P}$<br>$r = \dfrac{k_{\mathrm{cat}}[\mathrm{Xth}][\mathrm{DNA(AP_G)}]}{K_{\mathrm{DNA(AP_G)}} + [\mathrm{DNA(AP_G)}]}$	$k_{\mathrm{cat}} = 0.23\ \mathrm{s}^{-1}$<br>$K_{\mathrm{DNA(APG)}} = 1.6\times10^{-8}$ M	[8][f]<br>[8]
159	$\mathrm{DNA(AP_A)} + 2\ \mathrm{H_2O} \xrightarrow{Xth} \mathrm{DNA(dA)_{gap}} + 2\ \mathrm{H^+} + \mathrm{dR5P}$<br>$r = \dfrac{k_{\mathrm{cat}}[\mathrm{Xth}][\mathrm{DNA(AP_A)}]}{K_{\mathrm{DNA(AP_A)}} + [\mathrm{DNA(AP_A)}]}$	$k_{\mathrm{cat}} = 0.23\ \mathrm{s}^{-1}$<br>$K_{\mathrm{DNA(APA)}} = 1.6\times10^{-8}$ M	[8] [f]<br>[8]
160	$\mathrm{DNA(AP_C)} + 2\ \mathrm{H_2O} \xrightarrow{Xth} \mathrm{DNA(dC)_{gap}} + 2\ \mathrm{H^+} + \mathrm{dR5P}$<br>$r = \dfrac{k_{\mathrm{cat}}[\mathrm{Xth}][\mathrm{DNA(AP_C)}]}{K_{\mathrm{DNA(AP_C)}} + [\mathrm{DNA(AP_C)}]}$	$k_{\mathrm{cat}} = 0.23\ \mathrm{s}^{-1}$<br>$K_{\mathrm{DNA(APC)}} = 1.6\times10^{-8}$ M	[8] [f]<br>[8]
161	$\mathrm{DNA(dG)_{gap}} + \mathrm{dGTP} \xrightarrow{PolI} \mathrm{DNA(dG)_{nick}} + \mathrm{PP_i}$<br>$r = \dfrac{k_{\mathrm{cat}}[\mathrm{PolI}][\mathrm{dGTP}][\mathrm{DNA(dG)_{gap}}]}{K_{\mathrm{i,DNA(dG)_{gap}}} K_{\mathrm{dGTP}} + K_{\mathrm{DNA(dG)_{gap}}}[\mathrm{dGTP}] + K_{\mathrm{dGTP}}[\mathrm{DNA(dG)_{gap}}]\left(1 + \dfrac{K_{\mathrm{i,DNA(dG)_{gap}}}}{K_{\mathrm{i,DNA(dG)_{nick}}}}\right) + [\mathrm{DNA(dG)_{gap}}][\mathrm{dGTP}]\left(1 + \dfrac{K_{\mathrm{DNA(dG)_{gap}}}}{K_{\mathrm{i,DNA(dG)_{nick}}}}\right)}$	$k_{\mathrm{cat}} = 14\ \mathrm{s}^{-1}$<br>$K_{\mathrm{DNA(dG)gap}} = 5.4\times10^{-9}$ M<br>$K_{\mathrm{i,DNA(dG)gap}} = 8.1\times10^{-9}$ M<br>$K_{\mathrm{i,DNA(dG)nick}} = 2.2\times10^{-8}$ M<br>$K_{\mathrm{dGTP}} = 1.3\times10^{-6}$ M	[9][g]<br>[9]<br>[9]<br>[9]<br>[10][h]
162	$\mathrm{DNA(dA)_{gap}} + \mathrm{dATP} \xrightarrow{PolI} \mathrm{DNA(dA)_{nick}} + \mathrm{PP_i}$<br>$r = \dfrac{k_{\mathrm{cat}}[\mathrm{PolI}][\mathrm{dATP}][\mathrm{DNA(dA)_{gap}}]}{K_{\mathrm{i,DNA(dA)_{gap}}} K_{\mathrm{dATP}} + K_{\mathrm{DNA(dA)_{gap}}}[\mathrm{dATP}] + K_{\mathrm{dATP}}[\mathrm{DNA(dA)_{gap}}]\left(1 + \dfrac{K_{\mathrm{i,DNA(dA)_{gap}}}}{K_{\mathrm{i,DNA(dA)_{nick}}}}\right) + [\mathrm{DNA(dA)_{gap}}][\mathrm{dATP}]\left(1 + \dfrac{K_{\mathrm{DNA(dA)_{gap}}}}{K_{\mathrm{i,DNA(dA)_{nick}}}}\right)}$	$k_{\mathrm{cat}} = 14\ \mathrm{s}^{-1}$<br>$K_{\mathrm{DNA(dA)gap}} = 5.4\times10^{-9}$ M<br>$K_{\mathrm{i,DNA(dA)gap}} = 8.1\times10^{-9}$ M<br>$K_{\mathrm{i,DNA(dA)nick}} = 2.2\times10^{-8}$ M<br>$K_{\mathrm{dATP}} = 3.7\times10^{-6}$ M	[9] [g]<br>[9]<br>[9]<br>[9]<br>[10] [h]

163	$\mathrm{DNA(dC)_{gap} + dCTP \xrightarrow{PolI} DNA(dC)_{nick} + PP_i}$	k_{cat} = 14 s^{-1}<br>$K_{DNA(dC)gap}$ = 5.4×10^{-9} M<br>$K_{i,DNA(dC)gap}$ = 8.1×10^{-9} M<br>$K_{i,DNA(dC)nick}$ = 2.2×10^{-8} M<br>K_{dCTP} = 2.1×10^{-6} M	[9] [g]<br>[9]<br>[9]<br>[9]<br>[10] [h]
	$r = \dfrac{k_{cat}[\mathrm{PolI}][\mathrm{dCTP}][\mathrm{DNA(dC)_{gap}}]}{K_{i,\mathrm{DNA(dC)_{gap}}} K_{\mathrm{dCTP}} + K_{\mathrm{DNA(dC)_{gap}}}[\mathrm{dCTP}] + K_{\mathrm{dCTP}}[\mathrm{DNA(dC)_{gap}}]\left(1+\dfrac{K_{i,\mathrm{DNA(dC)_{gap}}}}{K_{i,\mathrm{DNA(dC)_{nick}}}}\right) + [\mathrm{DNA(dC)_{gap}}][\mathrm{dCTP}]\left(1+\dfrac{K_{\mathrm{DNA(dC)_{gap}}}}{K_{i,\mathrm{DNA(dC)_{nick}}}}\right)}$		
164	$\mathrm{DNA(dG)_{nick} + NAD^+ \xrightarrow{LigA} DNA(dG) + AMP + NMN + H^+}$	k_{cat} = 0.023 s^{-1}<br>K_{NAD+} = 7.0×10^{-6} M<br>$K_{DNA(dG)nick}$ = 5.0×10^{-8} M	[11] [i]<br>[11]<br>[11]
	$r = \dfrac{k_{cat}[\mathrm{LigA}][\mathrm{DNA(dG)_{nick}}][\mathrm{NAD^+}]}{[\mathrm{DNA(dG)_{nick}}][\mathrm{NAD^+}] + K_{\mathrm{NAD^+}}[\mathrm{DNA(dG)_{nick}}] + K_{\mathrm{DNA(dG)_{nick}}}[\mathrm{NAD^+}]}$		
165	$\mathrm{DNA(dA)_{nick} + NAD^+ \xrightarrow{LigA} DNA(dA) + AMP + NMN + H^+}$	k_{cat} = 0.023 s^{-1}<br>K_{NAD+} = 7.0×10^{-6} M<br>$K_{DNA(dA)nick}$ = 5.0×10^{-8} M	[11] [i]<br>[11]<br>[11]
	$r = \dfrac{k_{cat}[\mathrm{LigA}][\mathrm{DNA(dA)_{nick}}][\mathrm{NAD^+}]}{[\mathrm{DNA(dA)_{nick}}][\mathrm{NAD^+}] + K_{\mathrm{NAD^+}}[\mathrm{DNA(dA)_{nick}}] + K_{\mathrm{DNA(dA)_{nick}}}[\mathrm{NAD^+}]}$		
166	$\mathrm{DNA(dC)_{nick} + NAD^+ \xrightarrow{LigA} DNA(dC) + AMP + NMN + H^+}$	k_{cat} = 0.023 s^{-1}<br>K_{NAD+} = 7.0×10^{-6} M<br>$K_{DNA(dC)nick}$ = 5.0×10^{-8} M	[11] [i]<br>[11]<br>[11]
	$r = \dfrac{k_{cat}[\mathrm{LigA}][\mathrm{DNA(dC)_{nick}}][\mathrm{NAD^+}]}{[\mathrm{DNA(dC)_{nick}}][\mathrm{NAD^+}] + K_{\mathrm{NAD^+}}[\mathrm{DNA(dC)_{nick}}] + K_{\mathrm{DNA(dC)_{nick}}}[\mathrm{NAD^+}]}$		
167	$\mathrm{GSNO + 2\ NADH + 2\ H^+ + GSH \xrightarrow{GSFDH} GSSG + NH_3 + H_2O + 2\ NAD^+}$	k_{cat} = 3.1 s^{-1}<br>K_{GSNO} = 7.4×10^{-4} M	[12] [j]<br>[12]
	$r = \dfrac{k_{cat}[\mathrm{GSFDH}][\mathrm{GSNO}]}{K_{\mathrm{GSNO}} + [\mathrm{GSNO}]}$		
168	$\mathrm{GSSG + H^+ + NADPH \xrightarrow{Gor} 2\ GSH + NADP^+}$	k_1 = 267 s^{-1}<br>k_2 = 6.55×10^5 $M^{-1}s^{-1}$<br>K_{NADPH} = 2.2×10^{-5} M<br>K_{GSSG} = 9.7×10^{-5} M<br>K_1 = 0.022<br>K_2 = 3.9×10^3 M^{-1}	[13] [k]<br>[14]<br>[13]<br>[13]<br>[14]<br>[14]
	$r = \dfrac{k_1[\mathrm{Gor}][\mathrm{GSSG}][\mathrm{NADPH}] + k_2[\mathrm{Gor}][\mathrm{GSSG}]^2[\mathrm{NADPH}]}{K_{\mathrm{NADPH}}[\mathrm{GSSG}] + K_{\mathrm{GSSG}}[\mathrm{NADPH}] + [\mathrm{GSSG}][\mathrm{NADPH}] + K_1[\mathrm{GSSG}]^2 + K_2[\mathrm{GSSG}]^2[\mathrm{NADPH}]}$		

169	$Trx_{ox} + NADPH + H^+ \xrightarrow{TrxR} Trx_{red} + NADP^+$<br>$$r = \frac{k_{cat}[TrxR][Trx_{ox}][NADPH]}{[Trx_{ox}][NADPH] + K_{NADPH}[Trx_{ox}] + K_{Trx_{ox}}[NADPH]}$$	$k_{cat} = 41.25\ s^{-1}$<br>$K_{NADPH} = 4.6\times10^{-6}$ M<br>$K_{Trxox} = 1.7\times10^{-6}$ M	[15][l]<br>[15]<br>[15]
170	Trx_{red} + GSNO → Trx_{ox} + HNO + GSH<br>$$r = \frac{k_{cat}[Trx_{red}][GSNO]}{K_{GSNO} + [GSNO]}$$	$k_{cat} = 0.02\ s^{-1}$<br>$K_{GSNO} = 1.0\times10^{-5}$ M	[16]<br>[16]
171	Cyo + NO• ⇌ Cyo(NO)<br>$$r = \frac{k_{on,NO\bullet}[NO^\bullet][Cyo]}{1 + \frac{[O_2]}{K_{O_2}}} - k_{off,NO\bullet}[Cyo(NO)]$$	$k_{on,NO\bullet} = 6.8\times10^{6}\ M^{-1}s^{-1}$<br>$k_{off,NO\bullet} = 0.03\ s^{-1}$<br>$K_{\bullet} = 6.05\times10^{-6}$ M<br>$K_{d,NO\bullet} = 4.4\times10^{-9}$ M	[17][m]<br>[17]<br>[17]<br>[17]
172	Cyd + NO• ⇌ Cyd(NO)<br>$$r = \frac{k_{on,NO\bullet}[NO^\bullet][Cyd]}{1 + \frac{[O_2]}{K_{O_2}}} - k_{off,NO\bullet}[Cyd(NO)]$$	$k_{on,NO\bullet} = 3.8\times10^{8}\ M^{-1}s^{-1}$<br>$k_{off,NO\bullet} = 0.163\ s^{-1}$<br>$K_{O2} = 2.7\times10^{-7}$ M<br>$K_{d,NO\bullet} = 5.5\times10^{-10}$ M	[17][m]<br>[17]<br>[17]<br>[17]
173	$NorV_{ox}$ + NADH → $NorV_{red} + NAD^+ + H^+$<br>$$r = \frac{k_{cat}[NorV_{ox}][NADH]}{1 + \frac{[NO^\bullet]}{K_{i,NO\bullet}}}$$	$k_{cat} = 5.5\times10^{6}\ M^{-1}s^{-1}$<br>$K_{i,NO\bullet} = 1.35\times10^{-5}$ M	[18][n]<br>[19][o]
174	$NorV_{red}$ + 2 NO• + 2 H^+ → $NorV_{ox} + N_2O + H_2O$<br>$$r = \frac{k_{cat}[NorV_{red}][NO^\bullet]}{K_{NO\bullet} + [NO^\bullet]}$$	$k_{cat} = 7.45\ s^{-1}$<br>$K_{NO\bullet} = 1.2\times10^{-6}$ M	[20][p]<br>[21]

175	$NO\bullet + 6\ H^+ + 2.5\ NADH \rightarrow NH_4^+ + H_2O + 2.5\ NAD^+ + 2.5\ H^+$<br>$$r = \frac{k_{cat}[NrfA][NO^\bullet]}{K_{NO\bullet} + [NO^\bullet]}$$	$k_{cat} = 390\ s^{-1}$<br>$K_{NO\bullet} = 3.0\times10^{-4}$ M	[22][q]<br>[23][r]
176	$O_{2,air} \rightleftharpoons O_{2,culture}$<br>$$r = k_L a_{O_2}([O_2]_{sat} - [O_2])$$	$k_L a_{O2} = 1.36\times10^3\ s^{-1}$	*s*
177	$\rightarrow Hmp_{FAD,Fe3}$<br>$$r = \frac{k_{Hmp\text{-}exp,max}[NO^\bullet]}{[NO^\bullet] + K_{Hmp\text{-}exp,NO\bullet}}$$	$k_{Hmp\text{-}exp,max}$<br>$= 1.93 \times 10^{-8}\ M\cdot s^{-1}$ *<br>$K_{Hmp\text{-}exp,NO\bullet}$<br>$= 3.38 \times 10^{-7} M$ *	*t*
178	$\rightarrow NorV_{ox}$<br>$$r = \frac{k_{NorV\text{-}exp,max}[NO^\bullet]}{[NO^\bullet] + K_{NorV\text{-}exp,NO\bullet}}$$	$k_{NorV\text{-}exp,max}$<br>$= 6.81 \times 10^{-9}\ M\cdot s^{-1}$ *<br>$K_{NorV\text{-}exp,NO\bullet}$<br>$= 9.41 \times 10^{-6}$ M *	*t*
179	$\rightarrow NrfA$<br>$$r = \frac{k_{NrfA\text{-}exp,max}[NO_2^-]}{[NO_2^-] + K_{NrfA\text{-}exp,NO_2^-}\left(1 + \frac{[O_2]}{K_{NrfA\text{-}exp,O_2}}\right)}$$	$k_{NrfA\text{-}exp,max}$<br>$= 7.72 \times 10^{-9}\ M\cdot s^{-1}$ *<br>$K_{NrfA\text{-}exp,NO2-}$<br>$= 9.32 \times 10^{-4}$ M *<br>$K_{NrfA\text{-}exp,O2}$<br>$= 3.45 \times 10^{-11}$ M *	*t*

a. Literature k_{cat} is per mol cysteine; value shown has been halved according to the stoichiometry used.

b. Rate constants shown are for the reaction in the absence of the HscAB chaperone system. When present, the chaperone system has been shown to increase the [Fe-S] cluster transfer rate by approximately 10-fold [24].

c. [2Fe-2S] cluster transfer to an apoprotein was assumed to occur with both [2Fe-2S] and 2×[2Fe-2S]-loaded forms of IscU, and at similar rates.

d. The k_{cat} was calculated from the reported V_{max} by dividing by the concentration of AlkA enzyme used in the assay (300 fmol per 10 µl, or 30 nM).

e. The Michaelis constant for the deaminated base ($K_{DNA(dU)}$) was calculated from the constants associated with binding and unbinding (k_1 and k_{-1}, respectively) of the enzyme to the DNA strand [7], and the reported k_{cat} value [6], assuming intermediate enzyme complexes are in rapid equilibrium: $K_{DNA(dU)} = (k_{cat} + k_{-1})/k_1$.

f. Kinetics constants are for the rate of excision of deoxyribose (regular AP site) in oligonucleotides by Xth. For the reaction, we assumed the AP site and adjacent phosphate were removed in a single step to release 5dRP. The rate was also assumed to be independent of the base removed to form the AP site.

g. The rate expression was obtained from [9]. $K_{DNA(dN)gap}$ is the constant associated with the damaged DNA binding as a substrate, $K_{i,DNA(dN)gap}$ is the substrate inhibition constant for the damaged DNA, and $K_{i,DNA(dN)nick}$ is the constant describing product inhibition by the repaired DNA (where N = A, C, or G).
h. K_{dNTP} is the average of the two values reported for base re-insertion into two different types of template DNA strands.
i. Based on the reported reaction mechanism [11], we assumed ping-pong kinetics, and therefore used the corresponding form of the rate equation. The rate constants were also assumed to be independent of the nucleotides adjacent to the backbone nick. A proton (H^+) was added to the products of the reported reaction to balance stoichiometry.
j. The rate constants were approximated from Figure 1b in [12].
k. The rate expression was obtained from literature [14]. Values for rate constants k_2, K_1, and K_2 are for glutathione reductase in rat liver [14], while k_1, K_{NADPH}, and K_{GSSG} were measured for the *E. coli* enzyme [13].
l. Ping-pong kinetics were assumed based on the report of ping-pong behavior for human placenta thioredoxin reductase [25].
m. Reversible binding of NO• to Cyo and Cyd was modeled as following Michaelis-Menten behavior. Due to normal respiration activity (consumption of O_2), the effective enzyme concentration will be reduced. This was taken into account by adding a competitive inhibition term to the denominator, as O_2 and NO• are competing for the same active site.
n. Reported rate constant was measured at 5°C.
o. NorV is inhibited upon binding of NO• to its oxidized form. The inhibition rate constant shown is for the NO• reductase of *Paracoccus denitrificans*.
p. Literature k_{cat} is per mol NO•; value shown has been halved according to the stoichiometry used.
q. k_{cat} is reported to range from 30 to 1000 s^{-1} [22].
r. $K_{NO•}$ value was measured at 4°C.
s. The oxygen mass transfer coefficient ($k_L a_{O2}$) was measured in our experimental system (see Figure S12 and Text S1).
t. Parameters governing enzyme expression were determined via parameter optimization with experimentally measured [NO•] curves for aerobic, wild-type *E. coli* cultures dosed with 0.5 mM DPTA (see Table S4 and Text S1).

References

1. Urbina HD, Silberg JJ, Hoff KG, Vickery LE (2001) Transfer of Sulfur from IscS to IscU during Fe/S Cluster Assembly. J Biol Chem 276: 44521-44526.
2. Prischi F, Konarev PV, Iannuzzi C, Pastore C, Adinolfi S, et al. (2010) Structural bases for the interaction of frataxin with the central components of iron-sulphur cluster assembly. Nat Commun 1: 95.
3. Bonomi F, Iametti S, Ta D, Vickery LE (2005) Multiple Turnover Transfer of [2Fe2S] Clusters by the Iron-Sulfur Cluster Assembly Scaffold Proteins IscU and IscA. J Biol Chem 280: 29513-29518.
4. Terato H, Masaoka A, Asagoshi K, Honsho A, Ohyama Y, et al. (2002) Novel repair activities of AlkA (3-methyladenine DNA glycosylase II) and endonuclease VIII for xanthine and oxanine, guanine lesions induced by nitric oxide and nitrous acid. Nucleic Acids Res 30: 4975-4984.
5. Zhao BY, O'Brien PJ (2011) Kinetic Mechanism for the Excision of Hypoxanthine by Escherichia coli AlkA and Evidence for Binding to DNA Ends. Biochemistry 50: 4350-4359.
6. Wong I, Lundquist AJ, Bernards AS, Mosbaugh DW (2002) Presteady-state Analysis of a Single Catalytic Turnover by Escherichia coli Uracil-DNA Glycosylase Reveals a "Pinch-Pull-Push" Mechanism. J Biol Chem 277: 19424-19432.
7. Stivers JT, Pankiewicz KW, Watanabe KA (1999) Kinetic Mechanism of Damage Site Recognition and Uracil Flipping by Escherichia coli Uracil DNA Glycosylase. Biochemistry 38: 952-963.
8. Faure V, Saparbaev M, Dumy P, Constant JF (2005) Action of multiple base excision repair enzymes on the 2'-deoxyribonolactone. Biochem Biophys Res Commun 328: 1188-1195.
9. McClure WR, Jovin TM (1975) The Steady State Kinetic Parameters and Non-Processivity of Escherichia coli Deoxyribonucleic Acid Polymerase I. J Biol Chem 250: 4073-4080.
10. Bertram JG, Oertell K, Petruska J, Goodman MF (2010) DNA Polymerase Fidelity: Comparing Direct Competition of Right and Wrong dNTP Substrates with Steady State and Pre-Steady State Kinetics. Biochemistry 49: 20-28.
11. Lehman IR (1974) DNA Ligase: Structure, Mechanism, and Function. Science 186: 790-797.
12. Liu L, Hausladen A, Zeng M, Que L, Heitman J, et al. (2001) A metabolic enzyme for S-nitrosothiol conserved from bacteria to humans. Nature 410: 490-494.
13. Scrutton NS, Deonarain MP, Berry A, Perham RN (1992) Cooperativity Induced by a Single Mutation at the Subunit Interface of a Dimeric Enzyme: Glutathione Reductase. Science 258: 1140-1143.
14. Carlberg I, Mannervik B (1975) Purification and Characterization of the Flavoenzyme Glutathione Reductase from Rat Liver. J Biol Chem 250: 5475-5480.
15. Mulrooney SB (1997) Application of a Single-Plasmid Vector for Mutagenesis and High-Level Expression of Thioredoxin Reductase and Its Use to Examine Flavin Cofactor Incorporation. Protein Expr Purif 9: 372-378.
16. Sengupta R, Ryter SW, Zuckerbraun BS, Tzeng E, Billiar TR, et al. (2007) Thioredoxin Catalyzes the Denitrosation of Low-Molecular Mass and Protein S-Nitrosothiols. Biochemistry 46: 8472-8483.
17. Mason MG, Shepherd M, Nicholls P, Dobbin PS, Dodsworth KS, et al. (2009) Cytochrome bd confers nitric oxide resistance to Escherichia coli. Nat Chem Biol 5: 94-96.

18. Vicente JB, Scandurra FM, Rodrigues JV, Brunori M, Sarti P, et al. (2007) Kinetics of electron transfer from NADH to the Escherichia coli nitric oxide reductase flavorubredoxin. FEBS J 274: 677-686.
19. Girsch P, deVries S (1997) Purification and initial kinetic and spectroscopic characterization of NO reductase from Paracoccus denitrificans. Biochim Biophys Acta 1318: 202-216.
20. Gomes CM, Giuffrè A, Forte E, Vicente JB, Saraiva LM, et al. (2002) A Novel Type of Nitric-oxide Reductase. Escherichia coli Flavorubredoxin. J Biol Chem 277: 25273-25276.
21. Vicente JB, Scandurra FM, Forte E, Brunori M, Sarti P, et al. (2008) Kinetic Characterization of the Escherichia coli Nitric Oxide Reductase Flavorubredoxin. Methods Enzymol 437: 47-62.
22. Poock SR, Leach ER, Moir JW, Cole JA, Richardson DJ (2002) Respiratory Detoxification of Nitric Oxide by the Cytochrome c Nitrite Reductase of Escherichia coli. J Biol Chem 277: 23664-23669.
23. van Wonderen JH, Burlat B, Richardson DJ, Cheesman MR, Butt JN (2008) The Nitric Oxide Reductase Activity of Cytochrome c Nitrite Reductase from Escherichia coli. J Biol Chem 283: 9587-9594.
24. Bonomi F, Iametti S, Morleo A, Ta D, Vickery LE (2008) Studies on the Mechanism of Catalysis of Iron-Sulfur Cluster Transfer from IscU[2Fe2S] by HscA/HscB Chaperones. Biochemistry 47: 12795-12801.
25. Gromer S, Arscott LD, Williams CH, Jr., Schirmer RH, Becker K (1998) Human Placenta Thioredoxin Reductase. Isolation of the Selenoenzyme, Steady State Kinetics, and Inhibition by Therapeutic Gold Compounds. J Biol Chem 273: 20096-20101.
