# Supplementary material for: A Kinetic Platform to Determine the Fate of Nitric Oxide in Escherichia coli
Source: PLoS Comput Biol. 2013 May 2;9(5):e1003049. doi: 10.1371/journal.pcbi.1003049 (PMC3642044; doi:10.1371/journal.pcbi.1003049)
Supplement: Table S5 — Minimum biochemical reaction network necessary to simulate NO• dynamics in aerobic, wild-type E. coli cultures. (A) Reactions. Reaction numbers correspond to those used in Tables S2, S3, and Text S1. (B) Biochemical Species. Species numbers and initial concentrations (M) correspond to those reported in Table S1. (C) Kinetic parameters. “Reaction #s” are the numbers of the reactions governed by the rate parameter, and correspond to the numbering in Tables S2, S3, and Text S1. (PDF) [file pcbi.1003049.s019.pdf]

Table S5. Minimum biochemical reaction network necessary to simulate NO• dynamics in aerobic, wild-type *E. coli* cultures.

A. Reactions. Reaction numbers correspond to those used in Tables S2–S3, and Text S1.

Reaction #	Reaction equation	Description
1	2 NO• + O_2 → 2 NO_2•	NO• autoxidation
2	NO• + NO_2• → N_2O_3	NO• reaction with NO_2• (N_2O_3 formation)
98	$Hmp_{FAD,Fe3}$ + NADH + H^+ → $Hmp_{FADH2,Fe3}$ + NAD^+	Hmp FAD reduction by NADH
100	$Hmp_{FADH2,Fe3}$ → $Hmp_{FADH,Fe2}$ + H^+	Electron transfer from Hmp FAD to Fe^{3+}
101	$Hmp_{FADH,Fe2}$ + O_2 → $Hmp_{FADH,Fe2}(O_2)$	O_2 binding to Hmp-Fe^{2+}
103	$Hmp_{FADH,Fe2}(O_2)$ + NO• → $Hmp_{FADH,Fe3}(ONOO^-)$	NO• binding to Hmp-Fe^{2+}-O_2
104	$Hmp_{FADH,Fe3}(ONOO^-)$ → $Hmp_{FADH,Fe3}$ + NO_3^-	Hmp product (NO_3^-) release
105	$Hmp_{FADH,Fe3}$ → $Hmp_{FAD,Fe2}$ + H^+	Electron transfer from Hmp FAD to Fe^{3+}
106	$Hmp_{FAD,Fe2}$ + O_2 → $Hmp_{FAD,Fe2}(O_2)$	O_2 binding to Hmp-Fe^{2+}
108	$Hmp_{FAD,Fe2}(O_2)$ + NO• → $Hmp_{FAD,Fe3}(ONOO^-)$	NO• binding to Hmp-Fe^{2+}-O_2
109	$Hmp_{FAD,Fe3}(ONOO^-)$ → $Hmp_{FAD,Fe3}$ + NO_3^-	Hmp product (NO_3^-) release
113	$Hmp_{FAD,Fe2}$ + NO• → $Hmp_{FAD,Fe2}(NO)$	Inhibitory binding of NO• to Hmp-Fe^{2+}
115	$Hmp_{FAD,Fe2}(NO)$ → $Hmp_{FAD,Fe3}$ + NO^-	Slow reduction of bound NO• to NO^-
128	NONOate → 2 NO•	NO• release from chemical donor
129	NO• → *gas*	NO• transfer to the gas phase
176	$O_{2,air} \rightleftharpoons O_{2,culture}$	O_2 transfer to/from the gas phase
177	→ $Hmp_{FAD,Fe3}$	Hmp expression

B. Biochemical Species. Species #s and initial concentrations (M) correspond to those reported in Table S1.

Species #	Species	Description	Conc. (M)	Ref.
59	$Hmp_{FAD,Fe2}$	Nitric oxide dioxygenase (FAD, ferrous)	0	*a*
60	$Hmp_{FAD,Fe3}$	Nitric oxide dioxygenase (FAD, ferric)	0	*a*
61	$Hmp_{FADH,Fe2}$	Nitric oxide dioxygenase (FADH, ferrous)	0	*a*
62	$Hmp_{FADH,Fe3}$	Nitric oxide dioxygenase (FADH, ferric)	0	*a*
64	$Hmp_{FADH2,Fe3}$	Nitric oxide dioxygenase ($FADH_2$, ferric)	0	*a*
65	$Hmp_{FAD,Fe2}(O_2)$	Nitric oxide dioxygenase (FAD, ferrous), O_2-bound	0	*a*
66	$Hmp_{FAD,Fe2}$(NO•)	Nitric oxide dioxygenase (FAD, ferrous), NO•-bound	0	*a*
67	$Hmp_{FAD,Fe3}(ONOO^-)$	Nitric oxide dioxygenase (FAD, ferric), $ONOO^-$-bound	0	*a*
68	$Hmp_{FADH,Fe2}(O_2)$	Nitric oxide dioxygenase (FADH, ferrous), O_2-bound	0	*a*
70	$Hmp_{FADH,Fe3}(ONOO^-)$	Nitric oxide dioxygenase (FADH, ferric), $ONOO^-$-bound	0	*a*
80	N_2O_3	Nitrous anhydride	0	*a*
85	NADH	Nicotinamide adenine dinucleotide (reduced)	8.3×10^{-5}	[1]
92	NO^-	Nitroxyl anion	0	*a*
93	NO•	Nitric oxide	0	*a*
95	NO_2•	Nitrogen dioxide radical	0	*a*
96	NO_3^-	Nitrate	0	*a*
100	NONOate	1-Substituted diazen-1-ium-1,2-diolate (NO• donor)	5.0×10^{-4}	*b*
101	O_2	Oxygen	1.30×10^{-4}	*c*

a. Initial concentration assumed to be negligible.
b. Concentration of NONOate (DPTA) used for the experimental NO• measurement.
c. O_2 concentration measured at time of NONOate addition to *E. coli* culture (see Figure S13).

C. Kinetic parameters. "Reaction #s" are the numbers of the reactions governed by the rate parameter, and correspond to the numbering in Tables S2–S3, and Text S1.

Parameter	Parameter description/reaction involved	Reaction #s	Value	Units	Ref.
$k_{NONOate}$	NO• release from chemical donor	128	1.34×10^{-4}	s^{-1}	*a*
$k_La_{NO•}$	NO• transfer to the gas phase	129	4.74×10^{-3}	s^{-1}	*a*
$k_{NO•\text{-}O2}$	NO• autoxidation	1	1.80×10^{6}	$M^{-2}s^{-1}$	*a*
$k_{NO•\text{-}NO2•}$	NO• reaction with NO_2• (N_2O_3 formation)	2	1.10×10^{9}	$M^{-1}s^{-1}$	[2]
k_La_{O2}	O_2 transfer to/from the gas phase	176	1.37×10^{-3}	s^{-1}	*a*
$k_{Hmp\text{-}exp,max}$	Hmp expression (maximum rate)	177	1.82×10^{-8}	$M{\cdot}s^{-1}$	*b*
$K_{Hmp\text{-}exp,NO•}$	Hmp expression (regulatory NO• interaction)	177	4.65×10^{-8}	M	*b*
$k_{Hmp,NADH}$	Hmp detoxification; FAD reduction by NADH	98,116,123	7.00×10^{7}	$M^{-1}s^{-1}$	[3]
$k_{Hmp,ET}$	Hmp detoxification; electron transfer from FAD to Fe^{3+}	100,105	150	s^{-1}	[3]
$k_{Hmp,O2\text{-}on}$	Hmp detoxification; O_2 binding to Hmp-Fe^{2+}	101,106,121	7.50×10^{6}	$M^{-1}s^{-1}$	[3]
$k_{Hmp,NO•\text{-}ox}$	Hmp detoxification; NO• binding to Hmp-Fe^{2+}-O_2	103,108,125	1.24×10^{9}	$M^{-1}s^{-1}$	*b*
$k_{Hmp,P}$	Hmp detoxification; product (NO_3^-) release	104,109,126	200	s^{-1}	[3]
$k_{Hmp,NO•\text{-}on}$	Hmp detoxification; NO• binding to Hmp-Fe^{2+}	110,113,118	8.19×10^{6}	$M^{-1}s^{-1}$	*b*
$k_{Hmp,NO•\text{-}red}$	Hmp detoxification; reduction of bound NO• to NO^-	112,115,120	0.14	s^{-1}	[3]

a. Parameter measured in our experimental system.
b. Parameter value determined via optimization with experimentally-measured [NO•] curve (aerobic, wild-type *E. coli*).

References

1. Bennett BD, Kimball EH, Gao M, Osterhout R, Van Dien SJ, et al. (2009) Absolute metabolite concentrations and implied enzyme active site occupancy in Escherichia coli. Nat Chem Biol 5: 593-599.
2. Keszler A, Zhang Y, Hogg N (2010) Reaction between nitric oxide, glutathione, and oxygen in the presence and absence of protein: How are S-nitrosothiols formed? Free Radic Biol Med 48: 55-64.
3. Gardner AM, Martin LA, Gardner PR, Dou Y, Olson JS (2000) Steady-state and Transient Kinetics of Escherichia coli Nitric-oxide Dioxygenase (Flavohemoglobin): The B10 Tyrosine Hydroxyl is Essential for Dioxygen Binding and Catalysis. J Biol Chem 275: 12581-12589.
