# Supplementary material for: A Kinetic Platform to Determine the Fate of Nitric Oxide in Escherichia coli
Source: PLoS Comput Biol. 2013 May 2;9(5):e1003049. doi: 10.1371/journal.pcbi.1003049 (PMC3642044; doi:10.1371/journal.pcbi.1003049)
Supplement: Table S6 — Experimentally-accessible model parameters varied during parametric analysis. “Reaction #s” are the numbers of the reactions governed by the rate parameter, and correspond to the numbering in Tables S2, S3. Model parameters were varied among 5 logarithmically-spaced values spanning their allowed range. Allowed parameter ranges (defined by “Min.” and “Max.”) were chosen to span the value obtained or calculated from literature, unless otherwise noted. (PDF) [file pcbi.1003049.s020.pdf]

Table S6. Experimentally-accessible model parameters varied during parametric analysis. "Reaction #s" are the numbers of the reactions governed by the rate parameter, and correspond to the numbering in Tables S2–S3, and Text S1. Model parameters were varied among 5 logarithmically-spaced values spanning their allowed range. Allowed parameter ranges (defined by "Min." and "Max.") were chosen to span the value obtained or calculated from literature, unless otherwise noted.

#	Parameter	Parameter description/reaction involved	Reaction #s	Min.	Max.	Units	References
1	k_{NONOate}	NO• release from chemical donor	128	9.63×10^{-6}	5.78×10^{-3}	s^{-1}	[1][a]
2	$[\mathrm{NONOate}]_0$	Initial concentration of NONOate	--	5.0×10^{-5}	5.0×10^{-3}	M	[b]
3	$k_{\mathrm{Hmp\text{-}exp,max}}$	Hmp expression (maximum rate)	177	0	1.0×10^{-8}	$M \cdot s^{-1}$	[c]
4	$k_{\mathrm{NorV\text{-}exp,max}}$	NorV expression (maximum rate)	178	0	1.0×10^{-8}	$M \cdot s^{-1}$	[c]
5	$k_{\mathrm{NrfA\text{-}exp,max}}$	NrfA expression (maximum rate)	179	0	1.0×10^{-8}	$M \cdot s^{-1}$	[c]
6	$[\mathrm{Ala}]_0$	Initial concentration of L-alanine	--	1.0×10^{-3}	5.0×10^{-3}	M	[2]
7	$[\mathrm{AlkA}]_0$	Initial concentration of DNA glycosylase (dX, dI)	--	0	1.0×10^{-6}	M	[3]
8	$[\mathrm{ATP}]_0$	Initial concentration of ATP	--	1.0×10^{-3}	1.5×10^{-2}	M	[2,4]
9	$[\mathrm{Cys}]_0$	Initial concentration of L-cysteine	--	1.0×10^{-6}	5.0×10^{-4}	M	[5,6]
10	$[\mathrm{Cyd}]_0$	Initial concentration of cytochrome *bo*	--	0	1.0×10^{-5}	M	[3]
11	$[\mathrm{Cyo}]_0$	Initial concentration of cytochrome *bd*	--	0	1.0×10^{-5}	M	[3]
12	$[\mathrm{Gor}]_0$	Initial concentration of glutathione reductase	--	0	1.0×10^{-5}	M	[7]
13	$[\mathrm{GS\text{-}FDH}]_0$	Initial concentration of GSH-dependent FDH	--	0	1.0×10^{-5}	M	[d]
14	$[\mathrm{GSH}]_0$	Initial concentration of glutathione	--	0.001	0.05	M	[2]
15	$[P_{\mathrm{2Fe2S}}(holo)]_0$	Initial concentration of *holo* [2Fe-2S] proteins	--	1.0×10^{-6}	1.0×10^{-4}	M	[8,9]
16	$[P_{\mathrm{4Fe4S}}(holo)]_0$	Initial concentration of *holo* [4Fe-4S] proteins	--	1.0×10^{-5}	0.001	M	[8,9]
17	$[\mathrm{IscS}]_0$	Initial concentration of IscS	--	1.0×10^{-8}	1.0×10^{-5}	M	[10,11]
18	$[\mathrm{IscU}]_0$	Initial concentration of IscU	--	1.0×10^{-8}	1.0×10^{-5}	M	[10,11]
19	$[\mathrm{NADH}]_0$	Initial concentration of NADH	--	2.0×10^{-5}	2.0×10^{-4}	M	[2]
20	$[\mathrm{NADPH}]_0$	Initial concentration of NADPH	--	5.0×10^{-5}	5.0×10^{-4}	M	[2]
21	$[O_2]_0$	Initial concentration of O_2 (media, air, and cell)	--	0	2.5×10^{-4}	M	[e]
22	$[\mathrm{SOD}]_0$	Initial concentration of superoxide dismutase	--	0	1.0×10^{-4}	M	[12]
23	$[\mathrm{Trp}]_0$	Initial concentration of L-tryptophan	--	2.0×10^{-6}	1.0×10^{-4}	M	[2]
24	$[\mathrm{TrxR}]_0$	Initial concentration of thioredoxin reductase	--	0	1.0×10^{-5}	M	[d]
25	$[\mathrm{Tyr}]_0$	Initial concentration of L-tyrosine	--	1.0×10^{-6}	1.0×10^{-4}	M	[2]
26	$[\mathrm{Ung}]_0$	Initial concentration of DNA glycosylase (dU)	--	0	1.0×10^{-6}	M	[3]
27	$[\mathrm{Xth}]_0$	Initial concentration of DNA exonuclease III	--	0	1.0×10^{-6}	M	[3]

a. The minimum and maximum NONOate release rates correspond to half-lives of 20 h and 2 min, respectively. These values were selected based on the half-lives reported for the slow NO• donor, (Z)-1-[N-(2-aminoethyl)-N-(2-ammonioethyl)amino]diazen-1-ium-1,2-diolate (DETA NONOate), and the very rapid NO• donor, (Z)-1-[N-methyl-N-[6-(N-methylammoniohexyl)amino]]diazen-1-ium-1,2-diolate (MAHMA NONOate) [1].

b. NONOate concentration range was chosen as one order of magnitude below and above the concentration of NONOate used in this study (0.5 mM).

c. The maximum protein expression rates for Hmp, NorV, and NrfA were varied from zero (representing a deletion of the encoding gene) to a value based on the maximum expression rates reported for a number of enzymes in the study by Kotte *et al* [13].

d. Concentration was not found in literature, and therefore the maximum value was chosen based on values typically found for a number of other enzymes in the model.

e. The culture/intracellular O_2 concentration and the saturation O_2 concentration (in equilibrium with the gas in contact with the media) were varied as a single parameter.

References

1. Keefer LK, Nims RW, Davies KM, Wink DA (1996) "NONOates" (1-Substituted Diazen-1-ium-1,2-diolates) as Nitric Oxide Donors: Convenient Nitric Oxide Dosage Forms. Methods Enzymol 268: 281-293.
2. Bennett BD, Kimball EH, Gao M, Osterhout R, Van Dien SJ, et al. (2009) Absolute metabolite concentrations and implied enzyme active site occupancy in Escherichia coli. Nat Chem Biol 5: 593-599.
3. Taniguchi Y, Choi PJ, Li GW, Chen H, Babu M, et al. (2010) Quantifying E. coli Proteome and Transcriptome with Single-Molecule Sensitivity in Single Cells. Science 329: 533-538.
4. Buckstein MH, He J, Rubin H (2008) Characterization of Nucleotide Pools as a Function of Physiological State in Escherichia coli. J Bacteriol 190: 718-726.
5. Wheldrake JF (1967) Intracellular Concentration of Cysteine in Escherichia coli and its Relation to Repression of the Sulphate-Activating Enzymes. Biochem J 105: 697-699.
6. Park S, Imlay JA (2003) High Levels of Intracellular Cysteine Promote Oxidative DNA Damage by Driving the Fenton Reaction. J Bacteriol 185: 1942-1950.
7. Albe KR, Butler MH, Wright BE (1990) Cellular Concentrations of Enzymes and Their Substrates. J Theor Biol 143: 163-195.
8. Fontecave M (2006) Iron-sulfur clusters: ever-expanding roles. Nat Chem Biol 2: 171-174.
9. Sundararaj S, Guo A, Habibi-Nazhad B, Rouani M, Stothard P, et al. (2004) The CyberCell Database (CCDB): a comprehensive, self-updating, relational database to coordinate and facilitate in silico modeling of Escherichia coli. Nucleic Acids Res 32: D293-295.
10. Maynard ND, Macklin DN, Kirkegaard K, Covert MW (2012) Competing pathways control host resistance to virus via tRNA modification and programmed ribosomal frameshifting. Mol Syst Biol 8: 567.
11. Urbina HD, Silberg JJ, Hoff KG, Vickery LE (2001) Transfer of Sulfur from IscS to IscU during Fe/S Cluster Assembly. J Biol Chem 276: 44521-44526.
12. Imlay JA (2008) Cellular Defenses against Superoxide and Hydrogen Peroxide. Annu Rev Biochem 77: 755-776.
13. Kotte O, Zaugg JB, Heinemann M (2010) Bacterial adaptation through distributed sensing of metabolic fluxes. Mol Syst Biol 6: 355.
