# Supplementary material for: A Kinetic Platform to Determine the Fate of Nitric Oxide in Escherichia coli
Source: PLoS Comput Biol. 2013 May 2;9(5):e1003049. doi: 10.1371/journal.pcbi.1003049 (PMC3642044; doi:10.1371/journal.pcbi.1003049)
Supplement: Table S7 — Model parameters optimized for microaerobic conditions. “Reaction #s” are the numbers of the reactions governed by the rate parameter, and correspond to the numbering in Tables S2, S3, and Text S1. Allowed parameter ranges (defined by “Min.” and “Max.”) were chosen to encompass the value(s) obtained or calculated from literature, unless otherwise noted. “Optimal” are the parameter values from the optimization yielding the lowest SSR between the predicted and experimentally-measured [NO•] curve for wild-type E. coli treated with DPTA under microaerobic (35 µM O2) conditions. Confidence intervals (C.I.) are provided for parameters that were informed by the optimization, and were calculated as the range of optimal parameter values obtained for the top 10% of optimization outcomes (those with the lowest SSR values). (PDF) [file pcbi.1003049.s021.pdf]

Table S7. Model parameters optimized for microaerobic conditions. "Reaction #s" are the numbers of the reactions governed by the rate parameter, and correspond to the numbering in Tables S2–S3, and Text S1. Allowed parameter ranges (defined by "Min." and "Max.") were chosen to encompass the value(s) obtained or calculated from literature, unless otherwise noted. "Optimal" are the parameter values from the optimization yielding the lowest SSR between the predicted and experimentally-measured [NO•] curve for wild-type *E. coli* treated with DPTA under microaerobic (35 μM O_2) conditions. Confidence intervals (C.I.) are provided for parameters that were informed by the optimization, and were calculated as the range of optimal parameter values obtained for the top 10% of optimization outcomes (those with the lowest SSR values).

#	Parameter	Parameter description/reaction involved	Reaction #s	Min.	Max.	Optimal	C.I.	Units	Ref.
1	$k_{NONOate}$	NO• release from chemical donor	128	4.8×10^{-5}	3.9×10^{-4}	9.60×10^{-5}	9.60×10^{-5}–1.84×10^{-4}	s^{-1}	[1][a]
2	$k_La_{NO•}$	NO• transfer to the gas phase	129	0.001	0.05	2.10×10^{-2}	$(2.10–3.91) \times 10^{-2}$	s^{-1}	[b]
3	$k_{NO•\text{-}O2}$	NO• autoxidation	1	9.0×10^{5}	2.4×10^{6}	9.02×10^{5}	--	$M^{-2}s^{-1}$	[2]
4	$k_{NO•\text{-}[Fe\text{-}S]}$	[Fe-S] nitrosylation by NO•	85,86	1.0×10^{4}	1.0×10^{8}	1.92×10^{7}	--	$M^{-2}s^{-1}$	[3]
5	$k_{DNIC\text{-}rem}$	DNIC removal from protein	87,89	1	100	62.7	--	$M^{-1}s^{-1}$	[4]
6	$k_{DNIC\text{-}bind}$	DNIC binding to apoprotein	88,90	1	100	59.0	--	$M^{-1}s^{-1}$	[4]
7	$k_{DNIC\text{-}deg}$	O_2-mediated DNIC degradation	91	0.1	100	83.7	--	$M^{-1}s^{-1}$	[5]
8	$k_{IscU\text{-}load\text{-}Fe}$	IscA-mediated Fe^{2+} transfer to IscU	92,93	2.5×10^{-3}	2.5	1.68	--	s^{-1}	[6]
9	$K_{IscU\text{-}load\text{-}S,Cys}$	IscS-mediated S transfer from Cys to IscU	151,152	1.0×10^{-6}	1.0×10^{-4}	3.42×10^{-5}	--	M	[7]
10	$K_{IscU\text{-}load\text{-}S,IscU}$	IscS-mediated S transfer from Cys to IscU	151,152	1.0×10^{-6}	1.0×10^{-4}	1.66×10^{-5}	--	M	[7,8]
11	$k_{IscU\text{-}2Fe2S\text{-}insert,cat}$	IscU-mediated [2Fe-2S] insertion into apoprotein	153,154	1.0×10^{-4}	0.1	7.24×10^{-2}	--	s^{-1}	[9]
12	$K_{IscU\text{-}2Fe2S\text{-}insert,P2Fe2S(apo)}$	IscU-mediated [2Fe-2S] insertion into apoprotein	153,154	1.0×10^{-6}	1.0×10^{-4}	7.38×10^{-6}	--	M	[9]
13	$k_{IscU\text{-}4Fe4S\text{-}insert}$	IscU-mediated [4Fe-4S] insertion into apoprotein	94	1	500	398	--	$M^{-1}s^{-1}$	[10]
14	$k_{dN\text{-}deam}$	N_2O_3-mediated DNA base deamination	95–97	1.0×10^{3}	1.0×10^{6}	6.53×10^{3}	--	$M^{-1}s^{-1}$	[11]
15	$K_{dX\text{-}excis,DNA(dX)}$	Excision of xanthine from DNA	155	1.0×10^{-8}	1.0×10^{-6}	6.70×10^{-7}	--	M	[12]
16	$K_{dI\text{-}excis,DNA(dI)}$	Excision of hypoxanthine from DNA	156	1.0×10^{-8}	1.0×10^{-6}	8.49×10^{-7}	--	M	[13]
17	$K_{dU\text{-}excis,DNA(dU)}$	Excision of uracil from DNA	157	1.0×10^{-8}	1.0×10^{-6}	6.15×10^{-7}	--	M	[14]
18	$k_{Hmp,NO•\text{-}on}$	Hmp detoxification; NO• binding to Hmp-Fe^{2+}	110,113,118	4.0×10^{6}	2.6×10^{7}	4.29×10^{6}	$(4.29–8.07) \times 10^{6}$	$M^{-1}s^{-1}$	[15]
19	$k_{Hmp,NO•\text{-}ox}$	Hmp detoxification; NO• binding to Hmp-Fe^{2+}-O_2	103,108,125	9.6×10^{8}	2.4×10^{9}	1.79×10^{9}	--	$M^{-1}s^{-1}$	[15]
20	$k_{Hmp\text{-}exp,max}$	Hmp expression (maximum rate)	177	2.0×10^{-10}	2.0×10^{-8}	1.55×10^{-8}	$(1.12–2.00) \times 10^{-8}$	$M \cdot s^{-1}$	[c]
21	$K_{Hmp\text{-}exp,NO•}$	Hmp expression (regulatory NO• interaction)	177	1.0×10^{-8}	1.0×10^{-5}	1.59×10^{-6}	8.19×10^{-8}–2.06×10^{-6}	M	[d]
22	$k_{NorV\text{-}exp,max}$	NorV expression (maximum rate)	178	2.0×10^{-10}	2.0×10^{-8}	2.75×10^{-9}	9.58×10^{-10}–1.82×10^{-8}	$M \cdot s^{-1}$	[c]
23	$K_{NorV\text{-}exp,NO•}$	NorV expression (regulatory NO• interaction)	178	1.0×10^{-8}	1.0×10^{-5}	2.79×10^{-7}	2.79×10^{-7}–9.59×10^{-6}	M	[d]
24	$k_{NorV\text{-}O2}$	O_2-mediated NorV inactivation	146,147	10	1000	66.8	66.8–748	$M^{-1}s^{-1}$	[16]
25	$k_{NrfA\text{-}exp,max}$	NrfA expression (maximum rate)	179	2.0×10^{-10}	2.0×10^{-8}	8.06×10^{-9}	--	$M \cdot s^{-1}$	[c]
26	$K_{NrfA\text{-}exp,NO2-}$	NrfA expression (regulatory NO_2^- interaction)	179	1.0×10^{-6}	1.0×10^{-3}	5.02×10^{-4}	--	M	[e]
27	$K_{NrfA\text{-}exp,O2}$	NrfA expression (regulatory O_2 interaction)	179	1.0×10^{-12}	1.0×10^{-10}	5.14×10^{-11}	--	M	[e]
28	$[Cys]_0$	Initial concentration of cysteine	--	5.0×10^{-5}	2.0×10^{-4}	1.15×10^{-4}	--	M	[17,18]
29	$[Trx_{red}]_0$	Initial concentration of reduced thioredoxin	--	5.0×10^{-6}	5.0×10^{-5}	3.39×10^{-5}	--	M	[19,20]
30	$[IscU]_0$	Initial concentration of IscU	--	1.0×10^{-8}	1.0×10^{-5}	2.98×10^{-6}	--	M	[7,21]
31	$[IscS]_0$	Initial concentration of IscS	--	1.0×10^{-8}	1.0×10^{-5}	4.35×10^{-6}	--	M	[7,21]
32	$[P_{2Fe2S}(holo)]_0$	Initial concentration of *holo* [2Fe-2S] proteins	--	1.0×10^{-6}	1.0×10^{-4}	2.40×10^{-5}	--	M	[22,23]
33	$[P_{4Fe4S}(holo)]_0$	Initial concentration of *holo* [4Fe-4S] proteins	--	5.0×10^{-5}	5.0×10^{-4}	8.82×10^{-5}	--	M	[22,23]
34	$[LigA]_0$	Initial concentration of DNA ligase	--	1.0×10^{-8}	1.0×10^{-5}	9.38×10^{-8}	--	M	[24]

35	$[PolI]_0$	Initial concentration of DNA polymerase	--	1.0×10^{-8}	1.0×10^{-5}	9.54×10^{-6}	--	M	[24]
36	$[DNA(dN)]_0$	Initial concentration of DNA bases (dA,dC,dG)	--	0.001	0.1	6.46×10^{-2}	--	M	[23]
37	$[Xth]_0$	Initial concentration of DNA exonuclease III	--	1.0×10^{-9}	1.0×10^{-6}	7.58×10^{-7}	--	M	[25]
38	$[GS\text{-}FDH]_0$	Initial concentration of GSH-dependent FDH	--	1.0×10^{-8}	1.0×10^{-5}	4.89×10^{-6}	--	M	*f*
39	$[AlkA]_0$	Initial concentration of DNA glycosylase (dX, dI)	--	1.0×10^{-9}	1.0×10^{-6}	8.21×10^{-7}	--	M	[25]
40	$[Ung]_0$	Initial concentration of DNA glycosylase (dU)	--	1.0×10^{-9}	1.0×10^{-6}	5.30×10^{-7}	--	M	[25]
41	$[Cyo]_0$	Initial concentration of cytochrome *bo*	--	1.0×10^{-8}	1.0×10^{-5}	8.41×10^{-6}	--	M	[25]
42	$[Cyd]_0$	Initial concentration of cytochrome *bd*	--	1.0×10^{-8}	1.0×10^{-5}	6.29×10^{-6}	--	M	[25]

a. Range chosen based on typical half-lives reported for DPTA NONOate at 37°C [1].

b. Range selected based on the parameter value determined in our experimental system under aerobic conditions, without N_2 bubbling (4.74×10^{-3} s^{-1}). The bubbling was expected to increase the rate of NO• transfer to the gas phase, so the upper bound was chosen to be an order of magnitude greater than the value measured in the non-bubbling system.

c. The maximum protein expression rates for Hmp, NorV, and NrfA were not found in literature. The allowed parameter range was chosen based on the maximum expression rates reported for a number of enzymes in the work of Kotte *et al* [26]. Values were converted from the reported units of $g_{protein}/g_{DW}\cdot s$ to $M\cdot s^{-1}$ assuming a cell density of 448 gDW/L [23], and ranged from approximately 2×10^{-10} (Acs) to 2×10^{-8} $M\cdot s^{-1}$ (PfkA).

d. The allowed range for the NO• binding constant governing Hmp and NorV expression was chosen based on the reported physiological concentrations of NO• existing in the nM to µM range [27,28].

e. The NO_2^- binding constant governing NrfA expression was allowed to vary in the µM range, while the O_2 inhibition constant was assumed to be much lower, given that NrfA expression is primarily anaerobic [29,30].

f. Concentration was not found in literature, and therefore allowed a wide range, spanning values typically found for other enzymes in the model.

References

1. Keefer LK, Nims RW, Davies KM, Wink DA (1996) "NONOates" (1-Substituted Diazen-1-ium-1,2-diolates) as Nitric Oxide Donors: Convenient Nitric Oxide Dosage Forms. Methods Enzymol 268: 281-293.
2. Lewis RS, Deen WM (1994) Kinetics of the Reaction of Nitric Oxide with Oxygen in Aqueous Solutions. Chem Res Toxicol 7: 568-574.
3. Duan XW, Yang JJ, Ren BB, Tan GQ, Ding HG (2009) Reactivity of nitric oxide with the [4Fe-4S] cluster of dihydroxyacid dehydratase from Escherichia coli. Biochem J 417: 783-789.
4. Rogers PA, Ding HG (2001) L-Cysteine-mediated Destabilization of Dinitrosyl Iron Complexes in Proteins. J Biol Chem 276: 30980-30986.
5. Yang JJ, Duan XW, Landry AP, Ding HG (2010) Oxygen is required for the L-cysteine-mediated decomposition of protein-bound dinitrosyl-iron complexes. Free Radic Biol Med 49: 268-274.
6. Layer G, Ollagnier-de Choudens S, Sanakis Y, Fontecave M (2006) Iron-Sulfur Cluster Biosynthesis: Characterization of Escherichia coli CyaY as an Iron Donor for the Assembly of [2Fe-2S] Clusters in the Scaffold IscU. J Biol Chem 281: 16256-16263.
7. Urbina HD, Silberg JJ, Hoff KG, Vickery LE (2001) Transfer of Sulfur from IscS to IscU during Fe/S Cluster Assembly. J Biol Chem 276: 44521-44526.
8. Prischi F, Konarev PV, Iannuzzi C, Pastore C, Adinolfi S, et al. (2010) Structural bases for the interaction of frataxin with the central components of iron-sulphur cluster assembly. Nat Commun 1: 95.
9. Bonomi F, Iametti S, Ta D, Vickery LE (2005) Multiple Turnover Transfer of [2Fe2S] Clusters by the Iron-Sulfur Cluster Assembly Scaffold Proteins IscU and IscA. J Biol Chem 280: 29513-29518.
10. Unciuleac MC, Chandramouli K, Naik S, Mayer S, Huynh BH, et al. (2007) In Vitro Activation of Apo-Aconitase Using a [4Fe-4S] Cluster-Loaded Form of the IscU [Fe-S] Cluster Scaffolding Protein. Biochemistry 46: 6812-6821.
11. Dong M, Wang C, Deen WM, Dedon PC (2003) Absence of 2'-Deoxyoxanosine and Presence of Abasic Sites in DNA Exposed to Nitric Oxide at Controlled Physiological Concentrations. Chem Res Toxicol 16: 1044-1055.
12. Terato H, Masaoka A, Asagoshi K, Honsho A, Ohyama Y, et al. (2002) Novel repair activities of AlkA (3-methyladenine DNA glycosylase II) and endonuclease VIII for xanthine and oxanine, guanine lesions induced by nitric oxide and nitrous acid. Nucleic Acids Res 30: 4975-4984.
13. Zhao BY, O'Brien PJ (2011) Kinetic Mechanism for the Excision of Hypoxanthine by Escherichia coli AlkA and Evidence for Binding to DNA Ends. Biochemistry 50: 4350-4359.
14. Wong I, Lundquist AJ, Bernards AS, Mosbaugh DW (2002) Presteady-state Analysis of a Single Catalytic Turnover by Escherichia coli Uracil-DNA Glycosylase Reveals a "Pinch-Pull-Push" Mechanism. J Biol Chem 277: 19424-19432.
15. Gardner AM, Martin LA, Gardner PR, Dou Y, Olson JS (2000) Steady-state and Transient Kinetics of Escherichia coli Nitric-oxide Dioxygenase (Flavohemoglobin): The B10 Tyrosine Hydroxyl is Essential for Dioxygen Binding and Catalysis. J Biol Chem 275: 12581-12589.

16. Gardner AM, Gardner PR (2002) Flavohemoglobin Detoxifies Nitric Oxide in Aerobic, but not Anaerobic, Escherichia coli. Evidence for a Novel Inducible Anaerobic Nitric Oxide-scavenging Activity. J Biol Chem 277: 8166-8171.
17. Wheldrake JF (1967) Intracellular Concentration of Cysteine in Escherichia coli and its Relation to Repression of the Sulphate-Activating Enzymes. Biochem J 105: 697-699.
18. Park S, Imlay JA (2003) High Levels of Intracellular Cysteine Promote Oxidative DNA Damage by Driving the Fenton Reaction. J Bacteriol 185: 1942-1950.
19. Sengupta R, Ryter SW, Zuckerbraun BS, Tzeng E, Billiar TR, et al. (2007) Thioredoxin Catalyzes the Denitrosation of Low-Molecular Mass and Protein S-Nitrosothiols. Biochemistry 46: 8472-8483.
20. Lillig CH, Prior A, Schwenn JD, Aslund F, Ritz D, et al. (1999) New Thioredoxins and Glutaredoxins as Electron Donors of 3 '-Phosphoadenylylsulfate Reductase. J Biol Chem 274: 7695-7698.
21. Maynard ND, Macklin DN, Kirkegaard K, Covert MW (2012) Competing pathways control host resistance to virus via tRNA modification and programmed ribosomal frameshifting. Mol Syst Biol 8: 567.
22. Fontecave M (2006) Iron-sulfur clusters: ever-expanding roles. Nat Chem Biol 2: 171-174.
23. Sundararaj S, Guo A, Habibi-Nazhad B, Rouani M, Stothard P, et al. (2004) The CyberCell Database (CCDB): a comprehensive, self-updating, relational database to coordinate and facilitate in silico modeling of Escherichia coli. Nucleic Acids Res 32: D293-295.
24. Lehman IR (1974) DNA Ligase: Structure, Mechanism, and Function. Science 186: 790-797.
25. Taniguchi Y, Choi PJ, Li GW, Chen H, Babu M, et al. (2010) Quantifying E. coli Proteome and Transcriptome with Single-Molecule Sensitivity in Single Cells. Science 329: 533-538.
26. Kotte O, Zaugg JB, Heinemann M (2010) Bacterial adaptation through distributed sensing of metabolic fluxes. Mol Syst Biol 6: 355.
27. Allen BW, Liu J, Piantadosi CA (2005) Electrochemical Detection of Nitric Oxide in Biological Fluids. Methods Enzymol 396: 68-77.
28. Wang C, Trudel LJ, Wogan GN, Deen WM (2003) Thresholds of Nitric Oxide-Mediated Toxicity in Human Lymphoblastoid Cells. Chem Res Toxicol 16: 1004-1013.
29. Browning DF, Lee DJ, Spiro S, Busby SJ (2010) Down-Regulation of the Escherichia coli K-12 nrf Promoter by Binding of the NsrR Nitric Oxide-Sensing Transcription Repressor to an Upstream Site. J Bacteriol 192: 3824-3828.
30. Spiro S (2006) Nitric oxide-sensing mechanisms in Escherichia coli. Biochem Soc Trans 34: 200-202.
