# Supplementary material for: A Kinetic Platform to Determine the Fate of Nitric Oxide in Escherichia coli
Source: PLoS Comput Biol. 2013 May 2;9(5):e1003049. doi: 10.1371/journal.pcbi.1003049 (PMC3642044; doi:10.1371/journal.pcbi.1003049)
Supplement: Text S1 — Additional details on model development and analysis. Detailed model description, parametric analysis of model parameters with uncertain values, measurement of extracellular NO• kinetic parameters, measurement of O2 volumetric mass transfer coefficient (k L a O2), determination of O2 concentration prior to NO• stress, Hmp reaction mechanism and kinetics, O2-mediated inactivation of NorV, enzyme expression and degradation, and protein-bound DNIC removal and degradation. (PDF) [file pcbi.1003049.s022.pdf]

## Text S1. Additional details on model development and analysis.

### Detailed model description

*NO• autoxidation*

Many damaging effects of NO• result from the activity of its autoxidation products, including nitrogen dioxide ($NO_2$•), nitrous anhydride ($N_2O_3$), and dinitrogen tetroxide ($N_2O_4$) [1].

$2\ NO\bullet + O_2 \rightarrow 2\ NO_2\bullet$ *(Reaction 1, Table S2)*

$NO_2\bullet + NO\bullet \rightleftharpoons N_2O_3$ *(Reactions 2–3, Table S2)*

$2\ NO_2\bullet \rightleftharpoons N_2O_4$ *(Reactions 5–6, Table S2)*

The latter two products are subject to hydrolysis, yielding nitrite ($NO_2^-$) and nitrate ($NO_3^-$) [2].

$N_2O_3 + H_2O \rightarrow 2\ NO_2^- + 2\ H^+$ *(Reaction 4, Table S2)*

$N_2O_4 + H_2O \rightarrow NO_2^- + NO_3^- + 2\ H^+$ *(Reaction 7, Table S2)*

Although included in the model, the dimerization of $NO_2$• to form $N_2O_4$ is often neglected due to its relatively slow rate, as evidenced by the absence of $NO_3^-$ as a detectable product of NO• autoxidation in aqueous solution [1].

*Enzymatic NO• detoxification*

Nitric oxide dioxygenase (Hmp) has been shown to play a major role in protecting *E. coli* from NO• under aerobic conditions [3-5]. Hmp is a flavohemoglobin capable of catalyzing the NADH- (or NADPH-) dependent dioxygenation of NO• to $NO_3^-$ [5].

$$2\ NO\bullet + 2\ O_2 + NAD(P)H \xrightarrow{Hmp} 2\ NO_3^- + NAD(P)^+ + H^+$$

*(Reactions 98–126, Table S2)*

The reaction mechanism of Hmp-mediated dioxygenation of NO• and the associated rate constants were obtained from the literature [6]. Rather than use a single kinetic expression to describe the overall reaction rate, we treated the mechanism as a sub-network of individual reactions, including those involving inhibitory effects of NO•. Substrate inhibition occurs when NO• binds the reduced (ferrous) heme out of sequence (prior to $O_2$ binding) (Reactions 110, 113, 118, Table S2), and has an appreciable effect at even modest concentrations of NO• (~1% of the $O_2$ concentration) due to its ~1,000-fold higher affinity for the active site ferrous heme than that for $O_2$ [6]. The values of the rate constants governing the binding of NO• to the ferrous heme of Hmp ($k_{\mathrm{Hmp,NO\bullet\text{-}on}}$) and the reaction of NO• with the $O_2$-bound heme ($k_{\mathrm{Hmp,NO\bullet\text{-}ox}}$) were treated as uncertain (due to variation in reported values; see “Hmp reaction mechanism and kinetics” below), and varied during parametric analysis and optimization (Table S4).

We also included the $O_2$-independent NO• reductase function of Hmp which constitutes one mechanism to relieve substrate inhibition, whereby the ferrous-bound NO• is reduced, releasing $NO^-$ and the ferric heme [3,6,7]. The overall reduction reaction can be written as

$$2\ NO\bullet + NAD(P)H \xrightarrow{Hmp} 2\ NO^- + NAD(P)^+ + H^+$$

*(Reactions 112, 115, 120, Table S2)*

The intracellular concentration of Hmp in *E. coli* is reported to exist at levels below the limit of detection, unless induced by synthetic or environmental stimuli [4,8-10]. We therefore assumed a zero basal concentration, and modeled Hmp expression (Reaction 177, Table S3) as a function of intracellular NO• concentration (see "Enzyme expression and degradation" below) [11].

Flavorubredoxin reductase (NorV) is primarily responsible for the reduction of NO• to $N_2O$ under anoxic conditions, proceeding at a rate orders of magnitude greater than the Hmp-mediated reduction [3,12].

$$2\ NO\bullet + NADH + H^+ \xrightarrow{NorV} N_2O + NAD^+ + H_2O$$ *(Reactions 173–174, Table S3)*

The activity of NorV, however, is highly sensitive to $O_2$, decaying rapidly with a 5 min half-life under normoxia [3,13,14]. We included the $O_2$-dependent inactivation of NorV in the model (Reactions 146–147, Table S2) to capture the effects of $O_2$ on NorV activity. The rate constant governing $O_2$ inactivation was approximated from the reported rate of decay in activity under aerobic conditions (see "$O_2$-mediated inactivation of NorV" below) [3]. The production of NorV (Reaction 178, Table S3) was also modeled as a function of the intracellular NO• concentration,

where the basal expression rate (in the absence of NO•) was assumed to be negligible (see "Enzyme expression and degradation" below).

Another enzyme shown to protect *E. coli* during NO• stress in an anaerobic environment is the periplasmic formate-dependent nitrite reductase (NrfA) [15,16]. In addition to its role in reducing $NO_2^-$, NrfA is able to catalyze the 5-electron reduction of NO• to $NH_4^+$ [15].

$$NO\bullet + 2.5\ NADH + 6\ H^+ \xrightarrow{NrfA} NH_4^+ + 2.5\ NAD^+ + 2.5\ H^+ + H_2O$$

*(Reaction 175, Table S3)*

We obtained rate constants for NrfA-catalyzed NO• reduction from the literature [15,17], assuming Michaelis-Menten type kinetics. The expression of NrfA (Reaction 179, Table S3) depends heavily on the absence of $O_2$ (through FNR regulation), and exhibits a positive response to increased levels of NO• end-products, namely $NO_2^-$ and $NO_3^-$, rather than NO• itself [18-20]. We therefore modeled the synthesis of NrfA as a function of $NO_2^-$ concentration, with an $O_2$ repression term (see "Enzyme expression and degradation" below).

*Thiol S-nitrosation and denitrosation*

RNS can target low molecular weight (LMW) thiols to form various products, such as S-nitrosothiols and thiyl radicals [21]. Protein thiols are also subject to modification by RNS and other S-nitrosothiols, potentially affecting protein activity or function [22,23]. Thiols do not react directly with NO• at an appreciable rate [24], but rather with its oxidized forms ($NO_2$• and $N_2O_3$) [25,26]. GSH, the most abundant LMW thiol in enteric bacteria [27,28], reacts with $NO_2$• and $N_2O_3$ to form GS• and GSNO, respectively [25,26].

$$GSH + N_2O_3 \rightarrow GSNO + NO_2^- + H^+$$ *(Reaction 36, Table S2)*

$$GSH + NO_2\bullet \rightarrow GS\bullet + NO_2^- + H^+$$ *(Reaction 34, Table S2)*

The reaction network of thiols with RNS is extensive and complex, stretching far beyond the formation of GSNO and GS• [21,29-31]. The model incorporates nitrosation/denitrosation reactions collected from a number of studies [30,32-35], providing a thorough treatment of thiol-NO• chemistry (reactions 32–64, 76, 81, and 83–84 of Table S2, and reactions 167–170 of Table S3). One such denitrosation pathway is catalyzed by glutathione-dependent formaldehyde dehydrogenase (GS-FDH), which has a high specificity for GSNO [36].

$$GSNO + 2\ NADH + 2\ H^+ + GSH \xrightarrow{GSFDH} GSSG + NH_3 + H_2O + 2\ NAD^+$$

*(Reaction 167, Table S3)*

The overall reaction oxidizes two equivalents of NADH per GSNO denitrosated, producing $NH_3$ as the stable end-product [36].

Oxidative and nitrosative stress contribute to the oxidation of GSH to GSSG [29,30,37]. An enzyme responsible for catalyzing the reduction of GSSG to GSH is the NADPH-dependent glutathione reductase (Gor) [38].

$$GSSG + NADPH + H^+ \xrightarrow{Gor} 2\ GSH + NADP^+$$ *(Reaction 168, Table S3)*

The Gor mechanism follows either ping-pong or sequential kinetics, depending on whether the GSSG concentration is low or high, respectively [39-41]. We utilized a kinetic expression that

accounts for the concentration-dependent branching of flux among the two pathways [41], with rate parameters for GSSG and NADPH obtained for *E. coli* glutathione reductase [42]. We also included the reduction of oxidized thioredoxin by thioredoxin reductase (TrxR).

$$Trx_{ox} + NADPH + H^{+} \xrightarrow{TrxR} Trx_{red} + NADP^{+}$$ *(Reaction 169, Table S3)*

TrxR, reported to follow a ping-pong type mechanism [43], was modeled as such, using kinetic parameters measured for *E. coli* thioredoxin reductase [44].

*Nitroxyl (HNO) chemistry*

HNO and its conjugate base $NO^{-}$ are generated through processes such as thiol denitrosation and Hmp-catalyzed reduction of NO• [6,7,30]. Despite its relatively high pKa (11.4), the protonation of $NO^{-}$ by $H_2O$ is spin-forbidden and proceeds slower than would be expected ($k$ = 120 $s^{-1}$), thus increasing its lifetime [45]. A dominant reaction contributing to the consumption of HNO is its dimerization and dehydration to yield $N_2O$ as a stable end-product.

$$HNO + HNO \rightarrow N_2O + H_2O$$ *(Reaction 20, Table S2)*

Although $NO^{-}$ has been reported to react with $O_2$ to form $ONOO^{-}$ [45,46], the reaction was not included in the model due to more recent work that disagrees with $ONOO^{-}$ as a valid reaction product [47].

*Cytochrome inhibition*

An important effect of NO• is its inhibition of bacterial respiration by reversibly binding the terminal respiratory oxidases [48,49]. Under normal respiratory conditions, $O_2$ binds a heme at the active site of either of *E. coli*'s two terminal quinol oxidases, cytochromes *bo* and *bd* (collectively abbreviated here as Cyo/d), and is released as $H_2O$ following a two-electron reduction [4,50]. The *bo* and *bd* hemes have an affinity for NO• approximately 1,000-fold greater than for $O_2$, and are therefore strongly inhibited at even low NO• concentrations [51]. The model includes the reversible binding of NO• to Cyo and Cyd, acting as a temporary NO• sink and indicator of respiratory inhibition.

Cyo/d + NO• ⇌ Cyo/d-NO *(Reactions 171–172, Table S3)*

The binding of NO• to Cyo/d was modeled assuming reversible Michaelis-Menten kinetics, taking into account the binding competition with $O_2$ due to normal respiration activity. Kinetic constants governing interactions of NO• and $O_2$ with *E. coli* Cyo and Cyd were obtained from literature [51].

*$O_2{\bullet}^-$ generation and $ONOO^-$ formation*

A byproduct of cellular respiration, $O_2{\bullet}^-$ will react with NO• to form $ONOO^-$ at a near diffusion-controlled rate [52-54].

$NO{\bullet} + O_2{\bullet}^- \rightarrow ONOO^-$ *(Reaction 8, Table S2)*

When present, $CO_2$ may form an adduct with $ONOO^-$, which can decompose to $NO_3^-$, or $NO_2\bullet$ and $CO_3\bullet^-$ [53]. At neutral pH, $ONOO^-$ concentration is similar to that of its protonated form, peroxynitrous acid (ONOOH), which can also decompose to form $NO_3^-$ or $NO_2\bullet$, but in a $CO_2$-independent manner.

$ONOO^- + CO_2 \rightarrow NO_2\bullet + CO_3\bullet^-$ *(Reaction 65, Table S2)*

$ONOO^- + CO_2 \rightarrow NO_3^- + CO_2$ *(Reaction 66, Table S2)*

$ONOO^- + H^+ \rightleftharpoons ONOOH$ *(Reactions 12–13, Table S2)*

$ONOOH \rightarrow NO_3^- + H^+$ *(Reaction 14, Table S2)*

$ONOOH \rightarrow NO_2\bullet + \bullet OH$ *(Reaction 15, Table S2)*

The production of $O_2\bullet^-$ was modeled as a constant generation term of 2.9 μM/s, reflective of its generation within exponential-phase *E. coli* in minimal salts media [55]. Superoxide dismutase (Reaction 127, Table S2) was included as a sink for $O_2\bullet^-$, where the enzyme concentration [56] and kinetic parameters [57] obtained from literature corresponded to a steady-state $O_2\bullet^-$ concentration of $7 \times 10^{-11}$ M (in the absence of NO•), in agreement with reported values [55].

*Iron-sulfur cluster damage and repair*

Irreversible NO•-induced inactivation of iron-sulfur clusters is known to be critical to its bacteriostatic effect [11,58-61]. The present model includes nitrosative degradation and enzyme-mediated repair of the two most common cluster types in *E. coli*, [2Fe-2S] and [4Fe-4S] (Figure S1) [62,63]. Damage occurs upon rapid binding of NO• to the [2Fe-2S] or [4Fe-4S] cluster to

form protein-bound dinitrosyl iron complexes (DNICs) and/or Roussins' red esters (RREs) [61,64-66]. The cluster sulfurs are released as either elemental sulfur ($S^0$) or sulfide ($S^{2-}$) [65,67].

$$P_{2Fe2S}(holo)^{2-} + 4\ NO\bullet \rightarrow P_{2Fe2S}(DNIC)_2^{2-} + 2\ S^0 \qquad \textit{(Reaction 85, Table S2)}$$

$$P_{4Fe4S}(holo)^{2-} + 8\ NO\bullet \rightarrow P_{4Fe4S}(RRE)_2 + 3\ S^0 + S^{2-} \qquad \textit{(Reaction 86, Table S2)}$$

$P_{2Fe2S}(holo)^{2-}$ and $P_{4Fe4S}(holo)^{2-}$ are protein-bound [2Fe-2S] and [4Fe-4S] clusters, respectively. $P_{2Fe2S}(DNIC)_2^{2-}$ represents the two protein-bound DNICs following [2Fe-2S] cluster nitrosylation, while $P_{4Fe4S}(RRE)_2$ is a protein binding two RREs formed from [4Fe-4S]. Rates of [Fe-S] nitrosylation are highly dependent on the environment of the cluster, such as its proximity to the protein exterior, and nearby amino acid functional groups [68,69]. Duan *et al*. determined nitrosylation of the [4Fe-4S] cluster of *E. coli* dihydroxyacid dehydratase (IlvD), and similarly aconitase B (AcnB), to be second order in NO• with a rate constant of $(7.0 \pm 2.0) \times 10^6\ M^{-2}s^{-1}$, but found the reaction with the [4Fe-4S] in endonuclease III (Nth) to be relatively slow [70]. Due to the absence of kinetic data describing the nitrosylation of [2Fe-2S] clusters, we assumed NO• reacts similarly with [2Fe-2S] and [4Fe-4S] clusters. To address the variation in iron-sulfur cluster sensitivity to NO•, the rate of nitrosylation was treated as uncertain, and varied during parametric analysis and optimization.

The protein-bound DNICs and RREs originating from iron-sulfur cluster nitrosylation can undergo ligand exchange with free cysteine to release the complexes [64,71].

$$P_{2Fe2S}(DNIC)_2^{2-} + 4\ Cys \rightleftharpoons P_{2Fe2S}(apo) + 2\ DNIC(Cys)_2^{-}$$

*(Reactions 87–88, Table S2)*

$$P_{4Fe4S}(RRE)_2 + 8\ Cys \rightleftharpoons P_{4Fe4S}(apo) + 4\ DNIC(Cys)_2^- + 4\ H^+$$

*(Reactions 89–90, Table S2)*

Although the rate of DNIC extrusion is known to depend on free cysteine concentration [71-73], neither a rate equation nor associated kinetic constants were found in the literature. Rogers *et al*. used electron paramagnetic resonance (EPR) to measure the effect of varying cysteine concentration on the rate of disappearance of protein-bound DNICs in cell extracts of NO•-treated *E. coli* [73]. Using data from their work, we were able to estimate rate constants for the reactions (see "Protein-bound DNIC removal and degradation" below). Cysteine-mediated removal of RREs was assumed to proceed at the same rate as DNICs since the two species have been shown to interconvert prior to extrusion [71]. Free cysteine-bound DNICs are unstable in the presence of $O_2$ (~5 min half-life), decomposing to yield $Fe^{2+}$, $NO_2^-$, and cysteine (Reaction 91, Table S2) [72,74]. Since the mechanism and kinetics governing $O_2$-mediated DNIC degradation are poorly understood, we assumed bimolecular kinetics, and calculated the apparent rate constant from the reported half-life [72].

The repair of nitrosylated iron-sulfur clusters is reported to require *de novo* [Fe-S] cluster synthesis, proceeding through assembly on a scaffold protein, followed by insertion of the cluster into an apoprotein [75]. In *E. coli*, this process is mediated by a collection of enzymes comprising the Isc system, encoded by the *iscSUA-hscAB-fdx* gene cluster [76,77]. The alternative Suf system (*sufABCDSE*) is also capable of [Fe-S] assembly when Isc is unavailable, such as in conditions of oxidative stress or iron limitation [78]. Because the Isc and Suf systems exhibit similarities in their kinetics and mechanism of iron-sulfur cluster repair [77,79,80], only the Isc system was considered in the model.

The Isc-mediated assembly of iron-sulfur clusters begins with sulfur transfer from cysteine to an IscU scaffold protein by a cysteine desulfurase, IscS. Urbina *et al*. measured $k_{cat}$ and $K_{m,cys}$ (cysteine Michaelis-Menten constant) for the combined cysteine desulfurization and IscS-IscU sulfur transfer, but provide only the $K_d$ value for IscU [81]. A similar study [82] reported the $k_{off}$ value for the dissociation of IscU from the IscS-IscU complex, from which we were able to calculate $K_{m,IscU}$ using the relationship $K_m = (1 + k_{cat}/k_{off})K_d$. We modeled the reaction as a ping-pong mechanism, where cysteine binds to IscS, alanine is released, IscU binds to IscS, and sulfur-bound IscU is released. The overall reaction can be written as

$$\text{IscU} + 2\ \text{Cys} \xrightarrow{IscS} \text{IscU(2S)}^{2-} + 2\ \text{Ala} + 2\ \text{H}^{+}$$ *(Reaction 151, Table S3)*

This type of mechanism has also been observed for the analogous sulfur transfer reaction mediated by the Suf system [83,84]. The recruitment and transfer of $Fe^{2+}$ to IscU remains poorly understood, but has recently been attributed to IscA due to its high affinity for iron ($K_a = 3.0 \times 10^{19}\ M^{-1}$), and ability to supply iron for cluster assembly [80,85,86]. Frataxin (CyaY) has also been identified as a potential iron chaperone for [Fe-S] assembly [87], but its role appears to be more regulatory, as it demonstrates an inhibitory effect on cluster formation under certain conditions [88,89]. Due to the absence of kinetic data describing the IscA-mediated iron transfer, we instead used an apparent first-order rate constant reported for iron delivery by CyaY [87], recognizing that the actual rate of iron transfer to IscU will likely be faster. This potentially higher rate of iron delivery is assessed during parametric analysis.

After sulfur and $Fe^{2+}$ have been delivered to IscU, formation of a [2Fe-2S] cluster requires two electrons (in addition to the two supplied by the oxidation of both $Fe^{2+}$ to $Fe^{3+}$) to reduce the

sulfurs to sulfides ($S^{2-}$) [90]. Many studies performed experiments in the presence of reductants such as dithiothreitol or dithionite, and thus did not use the physiological reductant [80,91,92]. Ding *et al*. demonstrated that the thioredoxin reductase system (thioredoxin, thioredoxin reductase, and NADPH) was able to mediate iron binding and delivery to IscU, and provide a reducing environment sufficient for cluster assembly [93]. We therefore modeled $Fe^{2+}$ transfer and cluster reduction on IscU as a single reaction, using oxidation of reduced thioredoxin as the electron source.

$$\mathrm{IscU(2S)^{2-} + 2\ Fe^{2+} + Trx_{red}} \xrightarrow{IscA} \mathrm{IscU([2Fe\text{-}2S])^{2-} + Trx_{ox} + 4\ H^{+}}$$

*(Reactions 92–93, Table S2)*

Insertion of the IscU-bound [2Fe-2S] cluster into an apoprotein is mediated by the ATP-dependent HscAB system, which serves as a chaperone to increase efficiency of cluster delivery [94,95].

$$\mathrm{IscU([2Fe\text{-}2S])^{2-}} + P_{\mathrm{2Fe2S}}(apo) + \mathrm{ATP + H_2O} \xrightarrow{HscAB} \mathrm{IscU} + P_{\mathrm{2Fe2S}}(holo)^{2-} + \mathrm{ADP + P_i + H^{+}}$$

*(Reactions 153–154, Table S3)*

We obtained kinetic parameters for the [2Fe-2S] transfer from IscU to apo-ferredoxin, which was shown to exhibit Michaelis-Menten behavior [96]. The measurements, however, were in the absence of HscAB, so it is expected that the actual rate of [2Fe-2S] delivery is greater. The kinetic parameters were therefore varied during parametric analysis to assess their influence on system dynamics.

IscU forms a homo-dimer capable of housing two individual [2Fe-2S] clusters [75,92,97]. We assumed construction of the second [2Fe-2S] cluster on IscU follows the same kinetics as the first, and that both [2Fe-2S] and 2×[2Fe-2S] forms of IscU can transfer a [2Fe-2S] cluster to an apoprotein. The formation of a [4Fe-4S] cluster on IscU follows reductive coupling of two [2Fe-2S] clusters, where the electron source *in vivo* is suspected to be ferredoxin [63,75,97]. This reaction is relatively rapid [97], and was therefore merged with the subsequent reaction in which the [4Fe-4S] cluster is inserted into an apoprotein.

$$\mathrm{IscU([2Fe\text{-}2S]_2)^{4-}} + P_{\mathrm{4Fe4S}}(apo) + 2\ \mathrm{Fdx_{red}} + 4\ \mathrm{H^+} \rightarrow \mathrm{IscU} + P_{\mathrm{4Fe4S}}(holo)^{2-} + 2\ \mathrm{Fdx_{ox}}$$

*(Reaction 94, Table S2)*

Although the rate of [2Fe-2S] insertion was modeled to follow Michaelis-Menten kinetics [96], only an apparent second-order rate constant could be found for the insertion of [4Fe-4S] in the literature [92], and was therefore modeled as a bimolecular reaction.

*DNA deamination and repair*

DNA bases are subject to deamination upon exposure to nitrous anhydride ($N_2O_3$) which can cause transition mutations (AT → CG and CG → AT), strand breaks, and cross-links [98-101]. We included the deamination of adenine (A), cytosine (C), and guanine (G), to form hypoxanthine (hX), uracil (U), and xanthine (X), respectively, as well as their excision and replacement by the DNA base excision repair (BER) machinery (Figure S2) [102,103].

$$dA + N_2O_3 \rightarrow dhX + NO_2^- + N_2 + H^+ \qquad \textit{(Reaction 95, Table S2)}$$

$$dC + N_2O_3 \rightarrow dU + NO_2^- + N_2 + H^+ \qquad \textit{(Reaction 96, Table S2)}$$

$$dG + N_2O_3 \rightarrow dX + NO_2^- + N_2 + H^+ \qquad \textit{(Reaction 97, Table S2)}$$

To model deamination of DNA bases, second-order rate constants for the reaction of $N_2O_3$ with plasmid DNA were obtained from literature [104], and were essentially identical among the three bases (A,C, and G).

Repair of deaminated bases is primarily mediated by the BER system [103,105,106]. The model assumes that each of the deaminated residues follow the same BER pathway: (1) glycosydic cleavage of the deaminated base to generate an apurinic/apyrimidinic (AP) site [107-110], (2) cleavage of the DNA backbone and subsequent AP site excision by AP endonuclease [111,112], (3) nucleotide re-insertion catalyzed by DNA polymerase I [113,114], and (4) ligation of the nicked sugar-phosphate backbone [115] (Figure S2). The mechanism of base removal and backbone cleavage can vary depending on the deaminated base present and the enzymes involved (for example, bypassing the intermediate AP site, or simultaneous removal of an adjacent base/s) [103,111,112,116]. These alternative pathways were not considered, as they are upstream of DNA ligation, which has been identified as the rate-limiting step in *E. coli* BER [114].

If proceeding through an intermediate AP site, the removal of deaminated bases follows a multi-step mechanism whereby DNA glycosylase binds the affected site, “flips” the base out of the DNA helical space, cleaves the glycosidic bond, and releases the damaged base [117,118].

$$DNA(dN_{deam}) + H_2O \xrightarrow{Ung/AlkA} DNA(AP) + N_{deam} \qquad \textit{(Reactions 155–157, Table S3)}$$

Where DNA($dN_{deam}$) is the DNA-bound deaminated base, DNA(AP) is the resulting AP site, and $N_{deam}$ is the released deaminated base. Since the final step of this process, enzyme dissociation from the AP site, is rate limiting [119,120], we assumed the intermediate enzyme-substrate complexes exist in rapid equilibrium, and modeled the kinetics as Michaelis-Menten. In *E. coli*, uracil glycosylation is catalyzed by Ung, hypoxanthine by AlkA, and xanthine by AlkA or Nei [107].

The primary *E. coli* enzyme responsible for AP site removal under normal growth conditions is DNA exonuclease III (Xth) [112]. The enzyme processes the AP site via 5′ AP endonuclease and phosphatase activity, leaving 3′ OH and 5′ phosphate terminals, and releasing a free 2-deoxyribose 5-phosphate (dR5P) [110].

$$\mathrm{DNA(AP)} + 2\,\mathrm{H_2O} \xrightarrow{Xth} \mathrm{DNA_{gap}} + 2\,\mathrm{H^+} + \mathrm{dR5P} \qquad \textit{(Reactions 158–160, Table S3)}$$

The kinetics were modeled as Michaelis-Menten, using literature rate constants measured for Xth-mediated incision of an AP site [112]. Other possible mechanisms include cleavage of the DNA 5′ to the AP site, leaving the dR5P to be later removed by DNA polymerase, or a 3′ cleavage via β-elimination catalyzed by the enzyme AP lyase function [112]. These additional pathways were not included, however, as DNA ligation (downstream process) is reported to be rate-limiting step in the BER pathway [114].

After the damaged base or AP site has been processed by the upstream BER machinery, DNA polymerase I (PolI) facilitates the insertion of a new deoxyribonucleoside triphosphate (dNTP). The kinetics of the reaction were modeled as a sequential-type mechanism [113], where

DNA polymerase first binds DNA, then dNTP, followed by the release of $PP_i$, and dissociation of the enzyme from the DNA product.

$$DNA_{gap} + dNTP \xrightarrow{PolI} DNA(dN)_{nick} + PP_i$$ *(Reactions 161–163, Table S3)*

Rate constants for PolI-catalyzed insertion of dATP, dCTP, and dGTP were obtained from literature [113,121].

The final and rate-limiting [114] step in the BER pathway is ligation of the DNA backbone by DNA ligase. Unlike the DNA polymerase mechanism, the two-substrate (nicked DNA and $NAD^+$) ligation reaction was modeled as a ping-pong mechanism, where the first product (nicotinamide mononucleotide, NMN) is released prior to binding of the second substrate (nicked DNA) [115].

$$DNA(dN)_{nick} + NAD^+ \xrightarrow{LigA} DNA(dN) + AMP + NMN + H^+$$

*(Reactions 164–166, Table S3)*

The enzyme dissociates from the ligated strand, concluding the BER process.

**Parametric analysis of model parameters with uncertain values**

The majority (35 out of 39) of uncertain parameters had a negligible effect on the sum of the squared residuals (SSR) between the predicted and experimentally-measured NO• concentration profile for wild-type cells treated with DPTA (Figure S5). These 35 parameters were further analyzed, assessing their effect on the predicted distribution of NO• consumption among its

available pathways (such as autoxidation, loss to the gas phase, [Fe-S] nitrosylation, and Hmp-mediated detoxification). We first performed an individual parametric analysis, whereby each of the 35 parameters was varied among 5 logarithmically-spaced values spanning their allowed range (Table S4), and the resulting predicted distribution of NO• consumption was calculated. The analysis showed that none of the parameters had an appreciable effect on the overall or intracellular distribution of NO• (Figure S10). To further explore the effect of these parameters on the predicted NO• distribution, we also performed a combinatorial parametric analysis. Unfortunately, the size of the parametric space ($5^{35} = 3 \times 10^{24}$ possible combinations) precluded an exhaustive combinatorial parametric analysis. Therefore, we chose to analyze the solution space via random sampling. Parameters were randomly assigned a value within their allowed range (Table S4) for each simulation. A total of 100,000 different parameter sets were simulated, and the NO• distribution was calculated for each. Although greater changes in the distribution were observed for the combinatorial analysis than for the individual parametric analysis, the effects were still negligible (Figure S11).

**Measurement of extracellular NO• kinetic parameters**

Four parameters specific to the extracellular environment and growth media conditions were measured in our experimental apparatus: those associated with the rate of NO• donor dissociation ($k_{NONOate}$), NO• and $O_2$ transport to the gas phase ($k_L a_{NO\bullet}$ and $k_L a_{O2}$, respectively), and the rate of NO• autoxidation ($k_{NO\bullet\text{-}O2}$). The measurement of $k_L a_{O2}$ is discussed in the "$O_2$ volumetric mass transfer coefficient" section below. Under experimental conditions identical to those used for the NO• consumption assay described in Materials and methods (main text), we measured the concentration of NO• (ISO-NOP NO• sensor, World Precision Instruments, Inc.) for 2 h

following addition of 0.5 mM (Z)-1-[N-(3-aminopropyl)-N-(3-ammoniopropyl)amino]diazen-1-ium-1,2-diolate (DPTA NONOate) to MOPS buffer with 10 mM glucose (without cells) at 37°C. The final (10 h) concentrations of $NO_2^-$ and $NO_3^-$ were also measured (Nitrate/Nitrite Colorimetric Assay Kit, Cayman Chemical Company). The release of NO• in the media was simulated using the model, with the cellular volume fraction set to zero (to eliminate all intracellular reaction activity). Using a nonlinear least squares optimization algorithm (Materials and methods, main text), values were determined for $k_{NONOate}$ ($1.34 \times 10^{-4}$ $s^{-1}$, a 1.4 h half-life), $k_L a_{NO\bullet}$ ($4.74 \times 10^{-3}$ $s^{-1}$), and $k_{NO\bullet\text{-}O2}$ ($1.80 \times 10^6$ $M^{-2}s^{-1}$) which minimized the SSR between the predicted and measured [NO•] and [$NO_2^-$] data. The concentration of $NO_3^-$ measured was not found to differ significantly from the limit of detection (one sample *t*-test, 95% confidence), and thus considered negligible. Using the values determined for the three parameters, the predicted [NO•] curve and 10 h $NO_2^-$ and $NO_3^-$ concentrations were in excellent agreement with the experimental data (Figure S3).

The value of $k_{NONOate}$ for the alternate NO• donor used in this study, (Z)-1-[N-(3-aminopropyl)-N-(n-propyl)amino]diazen-1-ium-1,2-diolate (PAPA NONOate), was determined using a similar procedure as described above. MOPS media (without cells) was treated with 0.5 mM PAPA, and the resulting [NO•] curve was measured. The values of $k_L a_{NO\bullet}$, and $k_{NO\bullet\text{-}O2}$ were fixed to their previously-measured quantities, while only $k_{NONOate}$ was optimized, yielding a value of $1.35 \times 10^{-3}$ $s^{-1}$ (8.6 min half-life). The simulated NO• dynamics following media treatment with PAPA were in excellent agreement with the experimental data (Figure S7).

**Measurement of $O_2$ volumetric mass transfer coefficient ($k_L a_{O2}$)**

We calculated the $O_2$ volumetric mass transfer coefficient ($k_L a_{O2}$) for our experimental system from $O_2$ concentration measurements following $N_2$ flush of culture media. Experimental conditions were identical to those used for the NO• consumption assays (see Materials and methods, main text), except there were no cells present. 10 ml of MOPS glucose (10 mM) media in a 50 ml Falcon tube was stirred with a sterile magnetic stir bar, and immersed in a stirred water bath to maintain a constant temperature of 37°C. $N_2$ gas was bubbled into the media until most of the dissolved $O_2$ had been removed (~2% air saturation), after which the media was allowed to re-equilibrate with the surrounding air while $O_2$ concentration was measured with an $O_2$ sensor (FireStingO2, PyroScience GmbH). To calculate the $k_L a_{O2}$, an $O_2$ mass balance was applied on the system:

$$\frac{d[\mathrm{O}_2]}{dt} = k_{\mathrm{L}} a_{O_2} \left( [\mathrm{O}_2]_{\mathrm{sat}} - [\mathrm{O}_2] \right)$$

where $[O_2]$ is concentration of $O_2$ in the liquid media, $[O_2]_{sat}$ is the concentration of $O_2$ in the media in equilibrium with $O_2$ in the headspace air, assumed to be 185 µM [122]. Integration of the above equation yields the expression

$$\ln\left( [\mathrm{O}_2]_{\mathrm{sat}} - [\mathrm{O}_2] \right) = \ln\left( [\mathrm{O}_2]_{\mathrm{sat}} - [\mathrm{O}_2]_0 \right) - k_{\mathrm{L}} a_{\mathrm{O}_2} \cdot t$$

where $[O_2]_0$ is the initial concentration of $O_2$ in the media at time zero. Thus, during oxygenation of a degassed ($N_2$-flushed) solution, a plot of $\ln([O_2]_{sat} – [O_2])$ vs. time yields a line with slope

equal to $-k_L a_{O2}$. From our measurements, the $k_L a_{O2}$ for our system was calculated to be 4.92 $h^{-1}$ ($1.37 \times 10^{-3}$ $s^{-1}$) (Figure S12).

**Determination of $O_2$ concentration prior to NO• stress**

For the NO• consumption assays, cells were diluted to an $OD_{600}$ of 0.03 and allowed to grow to an $OD_{600}$ of 0.05 before the NONOate was delivered (to ensure cells remained in exponential phase). During this time, however, cellular respiration caused a modest decrease in the culture $O_2$ concentration. We therefore measured the $O_2$ concentration during this time, and found it to decrease to approximately 130 μM by the time the culture reached an $OD_{600}$ of 0.05 (the $OD_{600}$ at which NONOate was delivered) (Figure S13).

**Hmp reaction mechanism and kinetics**

The mechanism and kinetic parameters governing the Hmp-mediated detoxification of NO• were obtained from the literature [6]. The enzyme was modeled as a network of individual reactions, in accordance with their proposed scheme, rather than a single, lumped reaction (Table S2, Reactions 98–126). This allowed for us to more accurately represent Hmp activity by accounting for its NO• reductase activity, and the inhibitory effects of high NO• concentrations. To verify that the mechanism and rate parameters provided by Gardner and colleagues were in agreement with their experimental measurements [6], we simulated NO• consumption by the Hmp sub-network alone (no other reactions were included) using the Matlab *ode15s* function to numerically integrate the system of ODEs. The predicted Hmp activity (calculated as the net rate of NO• consumption per heme) was functionally equivalent to the overall rate equation provided (in the absence of NO• inhibition), and was able to reproduce their experimentally-measured

rates at a high $O_2$ concentration (670 μM). At lower $O_2$ concentrations (≤ 200 μM), calculations were in better agreement with the experimental data when using a $k_{\text{Hmp,NO•-ox}}$ (rate constant for the reaction of NO• with the $O_2$-bound active site) value of approximately half that reported. As such, the value of this parameter was treated as uncertain, and allowed to vary between the reported value ($2.4 \times 10^9$ $M^{-1}s^{-1}$) and the lower value calculated to agree with experimental data at lower $O_2$ concentrations ($9.6 \times 10^8$ $M^{-1}s^{-1}$). Unlike the overall rate expression describing Hmp activity, the simulated network was able to reproduce their experimental data under conditions of NO• inhibition. Experimental measurements by Gardner and colleagues report the inhibitory binding of NO• to the ferrous heme as exhibiting a heterogeneous rate, and provide two rate constant ($k_{\text{Hmp,NO•-on}}$) values ($4.0 \times 10^6$ $M^{-1}s^{-1}$ and $2.6 \times 10^7$ $M^{-1}s^{-1}$). This parameter was therefore allowed to vary during parametric analysis, between the two reported values [6].

**$O_2$-mediated inactivation of NorV**

NorV is irreversibly inactivated under aerobic conditions, where the activity has been measured to decay with a half-life ($t_{1/2}$) of 5 min in the presence of 200 μM $O_2$ [3]. A rate constant for the inactivation reaction ($k_{\text{NorV-O2}}$) was approximated from the bimolecular rate expression:

$$r = -k_{\text{NorV-O}_2}[\text{O}_2][\text{NorV}]$$

Following integration and rearrangement:

$$k_{\text{NorV-O}_2} = \frac{\ln(2)}{[\text{O}_2]\cdot t_{1/2}}$$

Using the values provided for $[O_2]$ and $t_{1/2}$, $k_{\text{NorV-O2}}$ was calculated to be 11.6 $M^{-1}s^{-1}$. This measured rate, however, did not fully account for the effects of autoxidation, which will reduce the $O_2$ concentration, and increase the rate of NO• consumption, both of which decrease the apparent rate of inactivation [19]. Furthermore, because Hmp is still expressed under anaerobic conditions [20,123], it is possible that some of the NO• consumption activity could be attributed to Hmp-mediated removal. Therefore, the $k_{\text{NorV-O2}}$ value of 11.6 $M^{-1}s^{-1}$ is likely an underestimate, and was treated as a lower bound during parametric analysis.

**Enzyme expression and degradation**

The concentration of enzymes involved in the detoxification of NO• (Hmp, NorV, and NrfA) change in response to NO• and/or its products. We therefore modeled their concentration as dynamic, incorporating expression and degradation terms to more accurately represent their participation in detoxifying NO•. The regulation of expression of each enzyme was approximated using a Hill equation with Hill coefficient $n = 1$, a functional form used previously to model protein expression as a function of transcription factor concentration [124]. A general protein degradation rate of $2.8 \times 10^{-5}$ $s^{-1}$ [124] was assumed for each of the enzymes.

*Hmp*

The regulation of Hmp is complex, involving a number of transcriptional regulators, such as FNR, NsrR, MetR, and NorR [11]. The expression of Hmp has been observed to increase significantly in response to NO• exposure [10,125,126]. We modeled the expression rate of Hmp (Reaction 177, Table S3) as a function of NO• concentration,

$$r = \frac{k_{\text{Hmp-exp,max}}[\text{NO}^\bullet]}{[\text{NO}^\bullet] + K_{\text{Hmp-exp,NO}\bullet}}$$

where $k_{\text{Hmp-exp,max}}$ is the maximum expression rate of Hmp, and $K_{\text{Hmp-exp,NO}\bullet}$ is a constant representing the interaction of NO• with transcriptional regulators. These parameters were treated as uncertain, and varied during parametric analysis and optimization (Table S4).

*NorV*

NorV activity is under the control of the NorR regulator, and is expressed in response to elevated concentrations of NO• [14,19,127]. Although NorV is inactivated under aerobic conditions [3], its expression does not appear to be a function of $O_2$ concentration [14]. The generation of NorV (Reaction 178, Table S3) was modeled as a function of NO• concentration.

$$r = \frac{k_{\text{NorV-exp,max}}[\text{NO}^\bullet]}{[\text{NO}^\bullet] + K_{\text{NorV-exp,NO}\bullet}}$$

As with the Hmp expression parameters, the NorV constants were varied during the analysis and optimization of model parameters (Table S4).

*NrfA*

NrfA expression is activated by the $O_2$-responsive regulator FNR, restricting its role to anaerobic environments [18,19]. Interestingly, it has been demonstrated that FNR is inactivated by NO• through nitrosylation of its [4Fe-4S] cluster [126], which may explain why NrfA expression has been shown, in some cases, to decrease in response to elevated levels of NO• [20]. Positive regulation of NrfA in response to NO• stress seen in other experiments may be the result of higher concentrations of NO• end-products, namely $NO_2^-$ [20]. Therefore, we modeled the expression of NrfA (Reaction 179, Table S3) as a function of $NO_2^-$ concentration, with an added $O_2$ inhibition term to capture its restriction to anaerobic environments.

$$r = \frac{k_{\text{NrfA-exp,max}}[\text{NO}_2^-]}{[\text{NO}_2^-] + K_{\text{NrfA-exp,NO}_2^-}\left(1 + \frac{[\text{O}_2]}{K_{\text{NrfA-exp,O}_2}}\right)}$$

The three parameters governing NrfA expression were varied during parametric analysis, and when optimizing model parameters (Table S4).

## Protein-bound DNIC removal and degradation

Protein-bound DNICs are extruded prior to reassembly of the active [Fe-S] cluster. This reversible process proceeds via ligand exchange with free cysteine [72].

$$P_{\text{FeS}}(\text{DNIC}) + 2\ \text{Cys} \rightleftharpoons P_{\text{FeS}}(apo) + \text{DNIC(Cys)}_2^-$$

Where $P_{FeS}$(DNIC) represents a protein-bound DNIC, and $P_{FeS}$(*apo*) is the apoprotein lacking its [Fe-S] cluster. Under aerobic conditions, the free cysteine-bound DNIC is unstable and degrades via an unknown mechanism to drive the equilibrium of the above reaction to the right [72].

$$\mathrm{DNIC(Cys)_2^{-} + O_2 + 2\ H^{+} + e^{-} \rightarrow 2\ Cys + Fe^{2+} + 2\ NO_2^{-}}$$

The rate constants governing the removal of protein-bound DNICs and their subsequent degradation were estimated from experimental data in the literature [72,73]. Rogers *et al.* measured relative EPR amplitudes corresponding to DNIC concentration after 20 minutes of aerobic incubation with varying thiol concentrations [73]. The cysteine-mediated DNIC removal was assumed to follow bimolecular kinetics, governed by the following expression:

$$\frac{d[P_{\mathrm{FeS}}(\mathrm{DNIC})]}{dt} = -k_{\mathrm{rem}}[P_{\mathrm{FeS}}(\mathrm{DNIC})][\mathrm{Cys}]$$

where $k_{rem}$ is the rate constant for DNIC removal, [$P_{FeS}$(DNIC)] and [Cys] are protein-bound DNIC and cysteine concentrations, respectively, and the reaction was assumed to be irreversible under aerobic conditions. We assumed a similar kinetic form for the $O_2$-dependent degradation of cysteine-bound DNICs:

$$\frac{d[\mathrm{DNIC(Cys)_2}]}{dt} = -k_{\mathrm{deg}}[\mathrm{DNIC(Cys)_2}][\mathrm{O_2}]$$

Given that both the free (cysteine-bound) and protein-bound DNICs are EPR-active [128], the relative amplitude of the EPR signal ($A_{\mathrm{EPR}}$) can be described by the following system of equations:

$$A_{\mathrm{EPR}} = \frac{[P_{\mathrm{FeS}}(\mathrm{DNIC})] + [\mathrm{DNIC(Cys)_2}]}{[P_{\mathrm{FeS}}(\mathrm{DNIC})]_0} = [P_{\mathrm{FeS}}(\mathrm{DNIC})]_{\mathrm{rel}} + [\mathrm{DNIC(Cys)_2}]_{\mathrm{rel}}$$

$$\frac{d[P_{\mathrm{FeS}}(\mathrm{DNIC})]_{\mathrm{rel}}}{dt} = -k_{\mathrm{rem}}[P_{\mathrm{FeS}}(\mathrm{DNIC})]_{\mathrm{rel}}[\mathrm{Cys}]$$

$$\frac{d[\mathrm{DNIC(Cys)_2}]_{\mathrm{rel}}}{dt} = k_{\mathrm{rem}}[P_{\mathrm{FeS}}(\mathrm{DNIC})]_{\mathrm{rel}}[\mathrm{Cys}]^2 - k_{\mathrm{deg}}[\mathrm{DNIC(Cys)_2}]_{\mathrm{rel}}[\mathrm{O_2}]$$

where subscripts "0" and "rel" indicate initial and relative DNIC concentrations, respectively. The degradation rate constant $k_{\mathrm{deg}}$ was calculated from the reported half-life of 5 minutes for $\mathrm{DNIC(Cys)_2}$ degradation in cell extracts under aerobic conditions [72]:

$$\frac{\ln(2)}{[\mathrm{O_2}]t_{1/2}} = k_{\mathrm{deg}}$$

Assuming an $O_2$ concentration of 185 µM [122], $k_{\mathrm{deg}}$ was calculated to be 12.5 $\mathrm{M^{-1}s^{-1}}$. We generated a Matlab function to numerically integrate (using the *ode15s* function) the above system of equations, returning a value for $A_{\mathrm{EPR}}$ at $t$ = 20 minutes (the time at which the experimental data were obtained). The concentration of cysteine was assumed constant in the simulations, as it is regenerated upon DNIC decomposition. The function was evaluated over a

range of cysteine concentrations to generate an $A_{EPR}(t = 20\ \text{min})$ vs. [Cys] curve to compare with the literature data [73]. The Matlab *lsqcurvefit* function was used to fit the value of $k_{rem}$ to the experimental data, which we calculated to be 15.3 $M^{-1}s^{-1}$. The comparison of simulation results with literature is shown in Figure S14. Under anaerobic environments, the process of DNIC removal has been shown to occur in the reverse direction, whereby free DNICs are transferred to iron-sulfur proteins [72]. We were unable to obtain kinetic data describing the reverse process, and thus treated the corresponding rate constant as uncertain. Its effect on system dynamics was evaluated during individual and combinatorial parametric analyses (Figures S10 and S11).

**References**


1. Lewis RS, Deen WM (1994) Kinetics of the Reaction of Nitric Oxide with Oxygen in Aqueous Solutions. Chem Res Toxicol 7: 568-574.
2. Goldstein S, Czapski G, Lind J, Merenyi G (2000) Tyrosine Nitration by Simultaneous Generation of (.)NO and O-(2) under Physiological Conditions. How the Radicals do the Job. J Biol Chem 275: 3031-3036.
3. Gardner AM, Gardner PR (2002) Flavohemoglobin Detoxifies Nitric Oxide in Aerobic, but not Anaerobic, Escherichia coli. Evidence for a Novel Inducible Anaerobic Nitric Oxide-scavenging Activity. J Biol Chem 277: 8166-8171.
4. Stevanin TM, Ioannidis N, Mills CE, Kim SO, Hughes MN, et al. (2000) Flavohemoglobin Hmp Affords Inducible Protection for Escherichia coli Respiration, Catalyzed by Cytochromes bo' or bd, from Nitric Oxide. J Biol Chem 275: 35868-35875.
5. Gardner PR, Gardner AM, Martin LA, Salzman AL (1998) Nitric oxide dioxygenase: An enzymic function for flavohemoglobin. Proc Natl Acad Sci U S A 95: 10378-10383.
6. Gardner AM, Martin LA, Gardner PR, Dou Y, Olson JS (2000) Steady-state and Transient Kinetics of Escherichia coli Nitric-oxide Dioxygenase (Flavohemoglobin): The B10 Tyrosine Hydroxyl is Essential for Dioxygen Binding and Catalysis. J Biol Chem 275: 12581-12589.
7. Kim SO, Orii Y, Lloyd D, Hughes MN, Poole RK (1999) Anoxic function for the Escherichia coli flavohaemoglobin (Hmp): reversible binding of nitric oxide and reduction to nitrous oxide. FEBS Lett 445: 389-394.
8. Anjum MF, Ioannidis N, Poole RK (1998) Response of the NAD(P)H-oxidising flavohaemoglobin (Hmp) to prolonged oxidative stress and implications for its physiological role in Escherichia coli. FEMS Microbiol Lett 166: 219-223.
9. Poole RK, Ioannidis N, Orii Y (1996) Reactions of the Escherichia coli flavohaemoglobin (Hmp) with NADH and near-micromolar oxygen: oxygen affinity of NADH oxidase activity. Microbiology 142: 1141-1148.
10. Poole RK, Anjum MF, Membrillo-Hernández J, Kim SO, Hughes MN, et al. (1996) Nitric Oxide, Nitrite, and Fnr Regulation of hmp (Flavohemoglobin) Gene Expression in Escherichia coli K-12. J Bacteriol 178: 5487-5492.
11. Spiro S (2007) Regulators of bacterial responses to nitric oxide. FEMS Microbiol Rev 31: 193-211.
12. Gomes CM, Giuffrè A, Forte E, Vicente JB, Saraiva LM, et al. (2002) A Novel Type of Nitric-oxide Reductase. Escherichia coli Flavorubredoxin. J Biol Chem 277: 25273-25276.
13. Gardner AM, Helmick RA, Gardner PR (2002) Flavorubredoxin, an Inducible Catalyst for Nitric Oxide Reduction and Detoxification in Escherichia coli. J Biol Chem 277: 8172-8177.
14. Gardner AM, Gessner CR, Gardner PR (2003) Regulation of the Nitric Oxide Reduction Operon (norRVW) in Escherichia coli. Role of NorR and sigma54 in the Nitric Oxide Stress Response. J Biol Chem 278: 10081-10086.
15. Poock SR, Leach ER, Moir JW, Cole JA, Richardson DJ (2002) Respiratory Detoxification of Nitric Oxide by the Cytochrome c Nitrite Reductase of Escherichia coli. J Biol Chem 277: 23664-23669.

16. Mills PC, Rowley G, Spiro S, Hinton JC, Richardson DJ (2008) A combination of cytochrome c nitrite reductase (NrfA) and flavorubredoxin (NorV) protects Salmonella enterica serovar Typhimurium against killing by NO in anoxic environments. Microbiology 154: 1218-1228.
17. van Wonderen JH, Burlat B, Richardson DJ, Cheesman MR, Butt JN (2008) The Nitric Oxide Reductase Activity of Cytochrome c Nitrite Reductase from Escherichia coli. J Biol Chem 283: 9587-9594.
18. Browning DF, Lee DJ, Spiro S, Busby SJ (2010) Down-Regulation of the Escherichia coli K-12 nrf Promoter by Binding of the NsrR Nitric Oxide-Sensing Transcription Repressor to an Upstream Site. J Bacteriol 192: 3824-3828.
19. Spiro S (2006) Nitric oxide-sensing mechanisms in Escherichia coli. Biochem Soc Trans 34: 200-202.
20. Pullan ST, Gidley MD, Jones RA, Barrett J, Stevanin TA, et al. (2007) Nitric Oxide in Chemostat-Cultured Escherichia coli Is Sensed by Fnr and Other Global Regulators: Unaltered Methionine Biosynthesis Indicates Lack of S Nitrosation. J Bacteriol 189: 1845-1855.
21. Hogg N, Singh RJ, Kalyanaraman B (1996) The role of glutathione in the transport and catabolism of nitric oxide. FEBS Lett 382: 223-228.
22. Hess DT, Matsumoto A, Kim SO, Marshall HE, Stamler JS (2005) Protein S-nitrosylation: purview and parameters. Nat Rev Mol Cell Biol 6: 150-166.
23. Seth D, Hausladen A, Wang YJ, Stamler JS (2012) Endogenous Protein S-Nitrosylation in E. coli: Regulation by OxyR. Science 336: 470-473.
24. Wink DA, Nims RW, Darbyshire JF, Christodoulou D, Hanbauer I, et al. (1994) Reaction Kinetics for Nitrosation of Cysteine and Glutathione in Aerobic Nitric Oxide Solutions at Neutral pH. Insights into the Fate and Physiological Effects of Intermediates Generated in the NO/O2 Reaction. Chem Res Toxicol 7: 519-525.
25. Ford E, Hughes MN, Wardman P (2002) Kinetics of the reactions of nitrogen dioxide with glutathione, cysteine, and uric acid at physiological pH. Free Radic Biol Med 32: 1314-1323.
26. Keshive M, Singh S, Wishnok JS, Tannenbaum SR, Deen WM (1996) Kinetics of S-Nitrosation of Thiols in Nitric Oxide Solutions. Chem Res Toxicol 9: 988-993.
27. Fang FC (2004) Antimicrobial reactive oxygen and nitrogen species: concepts and controversies. Nat Rev Microbiol 2: 820-832.
28. Bennett BD, Kimball EH, Gao M, Osterhout R, Van Dien SJ, et al. (2009) Absolute metabolite concentrations and implied enzyme active site occupancy in Escherichia coli. Nat Chem Biol 5: 593-599.
29. Singh SP, Wishnok JS, Keshive M, Deen WM, Tannenbaum SR (1996) The chemistry of the S-nitrosoglutathione/glutathione system. Proc Natl Acad Sci U S A 93: 14428-14433.
30. Wong PS-Y, Hyun J, Fukuto JM, Shirota FN, DeMaster EG, et al. (1998) Reaction between S-Nitrosothiols and Thiols: Generation of Nitroxyl (HNO) and Subsequent Chemistry. Biochemistry 37: 5362-5371.
31. Hogg N (2002) The Biochemistry and Physiology of S-nitrosothiols. Annu Rev Pharmacol Toxicol 42: 585-600.
32. Lim CH, Dedon PC, Deen WA (2008) Kinetic Analysis of Intracellular Concentrations of Reactive Nitrogen Species. Chem Res Toxicol 21: 2134-2147.

33. Keszler A, Zhang Y, Hogg N (2010) Reaction between nitric oxide, glutathione, and oxygen in the presence and absence of protein: How are S-nitrosothiols formed? Free Radic Biol Med 48: 55-64.
34. Kirsch M, Lehnig M, Korth HG, Sustmann R, de Groot H (2001) Inhibition of Peroxynitrite-Induced Nitration of Tyrosine by Glutathione in the Presence of Carbon Dioxide through both Radical Repair and Peroxynitrate Formation. Chemistry 7: 3313-3320.
35. Singh RJ, Hogg N, Joseph J, Kalyanaraman B (1996) Mechanism of Nitric Oxide Release from S-Nitrosothiols. J Biol Chem 271: 18596-18603.
36. Liu L, Hausladen A, Zeng M, Que L, Heitman J, et al. (2001) A metabolic enzyme for S-nitrosothiol conserved from bacteria to humans. Nature 410: 490-494.
37. Folkes LK, Wardman P (2004) Kinetics of the reaction between nitric oxide and glutathione: Implications for thiol depletion in cells. Free Radic Biol Med 37: 549-556.
38. Vanoni MA, Wong KK, Ballou DP, Blanchard JS (1990) Glutathione Reductase: Comparison of Steady-State and Rapid Reaction Primary Kinetic Isotope Effects Exhibited by the Yeast, Spinach, and Escherichia coli Enzymes. Biochemistry 29: 5790-5796.
39. Mannervik B (1973) A branching reaction mechanism of glutathione reductase. Biochem Biophys Res Commun 53: 1151-1158.
40. Ulusu NN, Tandoğan B (2007) Purification and kinetic properties of glutathione reductase from bovine liver. Mol Cell Biochem 303: 45-51.
41. Carlberg I, Mannervik B (1975) Purification and Characterization of the Flavoenzyme Glutathione Reductase from Rat Liver. J Biol Chem 250: 5475-5480.
42. Scrutton NS, Deonarain MP, Berry A, Perham RN (1992) Cooperativity Induced by a Single Mutation at the Subunit Interface of a Dimeric Enzyme: Glutathione Reductase. Science 258: 1140-1143.
43. Gromer S, Arscott LD, Williams CH, Jr., Schirmer RH, Becker K (1998) Human Placenta Thioredoxin Reductase. Isolation of the Selenoenzyme, Steady State Kinetics, and Inhibition by Therapeutic Gold Compounds. J Biol Chem 273: 20096-20101.
44. Mulrooney SB (1997) Application of a Single-Plasmid Vector for Mutagenesis and High-Level Expression of Thioredoxin Reductase and Its Use to Examine Flavin Cofactor Incorporation. Protein Expr Purif 9: 372-378.
45. Shafirovich V, Lymar SV (2002) Nitroxyl and its anion in aqueous solutions: Spin states, protic equilibria, and reactivities toward oxygen and nitric oxide. Proc Natl Acad Sci U S A 99: 7340-7345.
46. Liochev SI, Fridovich I (2003) The mode of decomposition of Angeli's salt (Na2N2O3) and the effects thereon of oxygen, nitrite, superoxide dismutase, and glutathione. Free Radic Biol Med 34: 1399-1404.
47. Jackson MI, Han TH, Serbulea L, Dutton A, Ford E, et al. (2009) Kinetic feasibility of nitroxyl reduction by physiological reductants and biological implications. Free Radic Biol Med 47: 1130-1139.
48. Yu H, Sato EF, Nagata K, Nishikawa M, Kashiba M, et al. (1997) Oxygen-dependent regulation of the respiration and growth of Escherichia coli by nitric oxide. FEBS Lett 409: 161-165.
49. Mason MG, Holladay RS, Nicholls P, Shepherd M, Cooper CE (2008) A Quantitative Approach to Nitric Oxide Inhibition of Terminal Oxidases of the Respiratory Chain. Methods Enzymol 437: 135-159.

50. Poole RK, Cook GM (2000) Redundancy of Aerobic Respiratory Chains in Bacteria? Routes, Reasons and Regulation. Adv Microb Physiol 43: 165-224.
51. Mason MG, Shepherd M, Nicholls P, Dobbin PS, Dodsworth KS, et al. (2009) Cytochrome bd confers nitric oxide resistance to Escherichia coli. Nat Chem Biol 5: 94-96.
52. Blough NV, Zafiriou OC (1985) Reaction of Superoxide with Nitric Oxide to Form Peroxonitrite in Alkaline Aqueous Solution. Inorg Chem 24: 3502-3504.
53. Squadrito GL, Pryor WA (1998) Oxidative chemistry of nitric oxide: The roles of superoxide, peroxynitrite, and carbon dioxide. Free Radic Biol Med 25: 392-403.
54. Kissner R, Nauser T, Bugnon P, Lye PG, Koppenol WH (1997) Formation and Properties of Peroxynitrite as Studied by Laser Flash Photolysis, High-Pressure Stopped-Flow Technique, and Pulse Radiolysis. Chem Res Toxicol 10: 1285-1292.
55. Imlay JA, Fridovich I (1991) Assay of Metabolic Superoxide Production in Escherichia coli. J Biol Chem 266: 6957-6965.
56. Imlay JA (2008) Cellular Defenses against Superoxide and Hydrogen Peroxide. Annu Rev Biochem 77: 755-776.
57. Falconi M, O'Neill P, Stroppolo ME, Desideri A (2002) Superoxide Dismutase Kinetics. Methods Enzymol 349: 38-49.
58. Ren B, Zhang N, Yang J, Ding H (2008) Nitric oxide-induced bacteriostasis and modification of iron-sulphur proteins in Escherichia coli. Mol Microbiol 70: 953-964.
59. Gardner PR, Costantino G, Szabo C, Salzman AL (1997) Nitric Oxide Sensitivity of the Aconitases. J Biol Chem 272: 25071-25076.
60. Reddy D, Lancaster JR, Jr., Cornforth DP (1983) Nitrite Inhibition of Clostridium botulinum: Electron Spin Resonance Detection of Iron-Nitric Oxide Complexes. Science 221: 769-770.
61. Foster HW, Cowan JA (1999) Chemistry of Nitric Oxide with Protein-Bound Iron Sulfur Centers. Insights on Physiological Reactivity. J Am Chem Soc 121: 4093-4100.
62. Fontecave M (2006) Iron-sulfur clusters: ever-expanding roles. Nat Chem Biol 2: 171-174.
63. Ayala-Castro C, Saini A, Outten FW (2008) Fe-S Cluster Assembly Pathways in Bacteria. Microbiol Mol Biol Rev 72: 110-125.
64. Butler AR, Glidewell C, Hyde AR, Walton JC (1985) Formation of Paramagnetic Mononuclear Iron Nitrosyl Complexes from Diamagnetic Di- and Tetranuclear Iron-Sulphur Nitrosyls: Characterisation by EPR Spectroscopy and Study of Thiolate and Nitrosyl Ligand Exchange Reactions. Polyhedron 4: 797-809.
65. Crack JC, Smith LJ, Stapleton MR, Peck J, Watmough NJ, et al. (2011) Mechanistic Insight into the Nitrosylation of the [4Fe-4S] Cluster of WhiB-like Proteins. J Am Chem Soc 133: 1112-1121.
66. Tinberg CE, Tonzetich ZJ, Wang HX, Do LH, Yoda Y, et al. (2010) Characterization of Iron Dinitrosyl Species Formed in the Reaction of Nitric Oxide with a Biological Rieske Center. J Am Chem Soc 132: 18168-18176.
67. Harrop TC, Tonzetich ZJ, Reisner E, Lippard SJ (2008) Reactions of Synthetic [2Fe-2S] and [4Fe-4S] Clusters with Nitric Oxide and Nitrosothiols. J Am Chem Soc 130: 15602-15610.
68. Varghese S, Tang Y, Imlay JA (2003) Contrasting Sensitivities of Escherichia coli Aconitases A and B to Oxidation and Iron Depletion. J Bacteriol 185: 221-230.
69. Crack JC, Green J, Thomson AJ, Le Brun NE (2012) Iron-sulfur cluster sensor-regulators. Curr Opin Chem Biol 16: 35-44.

70. Duan XW, Yang JJ, Ren BB, Tan GQ, Ding HG (2009) Reactivity of nitric oxide with the [4Fe-4S] cluster of dihydroxyacid dehydratase from Escherichia coli. Biochem J 417: 783-789.
71. Lin ZS, Lo FC, Li CH, Chen CH, Huang WN, et al. (2011) Peptide-Bound Dinitrosyliron Complexes (DNICs) and Neutral/Reduced-Form Roussin's Red Esters (RREs/rRREs): Understanding Nitrosylation of [Fe-S] Clusters Leading to the Formation of DNICs and RREs Using a De Novo Design Strategy. Inorg Chem 50: 10417-10431.
72. Yang JJ, Duan XW, Landry AP, Ding HG (2010) Oxygen is required for the L-cysteine-mediated decomposition of protein-bound dinitrosyl-iron complexes. Free Radic Biol Med 49: 268-274.
73. Rogers PA, Ding HG (2001) L-Cysteine-mediated Destabilization of Dinitrosyl Iron Complexes in Proteins. J Biol Chem 276: 30980-30986.
74. Boese M, Mordvintcev PI, Vanin AF, Busse R, Mülsch A (1995) S-Nitrosation of Serum Albumin by Dinitrosyl-Iron Complex. J Biol Chem 270: 29244-29249.
75. Bandyopadhyay S, Chandramouli K, Johnson MK (2008) Iron-sulfur cluster biosynthesis. Biochem Soc Trans 36: 1112-1119.
76. Zheng LM, Cash VL, Flint DH, Dean DR (1998) Assembly of Iron-Sulfur Clusters: Identification of an iscSUA-hscBA-fdx Gene Cluster from Azotobacter vinelandii. J Biol Chem 273: 13264-13272.
77. Takahashi Y, Nakamura M (1999) Functional Assignment of the ORF2-iscS-iscU-iscA-hscB-hscA-fdx-ORF3 Gene Cluster Involved in the Assembly of Fe-S Clusters in Escherichia coli. J Biochem 126: 917-926.
78. Jang S, Imlay JA (2010) Hydrogen peroxide inactivates the Escherichia coli Isc iron-sulphur assembly system, and OxyR induces the Suf system to compensate. Mol Microbiol 78: 1448-1467.
79. Lu JX, Yang JJ, Tan GQ, Ding HG (2008) Complementary roles of SufA and IscA in the biogenesis of iron-sulfur clusters in Escherichia coli. Biochem J 409: 535-543.
80. Ding HG, Clark RJ (2004) Characterization of iron binding in IscA, an ancient iron-sulphur cluster assembly protein. Biochem J 379: 433-440.
81. Urbina HD, Silberg JJ, Hoff KG, Vickery LE (2001) Transfer of Sulfur from IscS to IscU during Fe/S Cluster Assembly. J Biol Chem 276: 44521-44526.
82. Prischi F, Konarev PV, Iannuzzi C, Pastore C, Adinolfi S, et al. (2010) Structural bases for the interaction of frataxin with the central components of iron-sulphur cluster assembly. Nat Commun 1: 95.
83. Albrecht AG, Peuckert F, Landmann H, Miethke M, Seubert A, et al. (2011) Mechanistic characterization of sulfur transfer from cysteine desulfurase SufS to the iron-sulfur scaffold SufU in Bacillus subtilis. FEBS Lett 585: 465-470.
84. Selbach B, Earles E, Dos Santos PC (2010) Kinetic Analysis of the Bisubstrate Cysteine Desulfurase SufS from Bacillus subtilis. Biochemistry 49: 8794-8802.
85. Ding B, Smith ES, Ding H (2005) Mobilization of the iron centre in IscA for the iron-sulphur cluster assembly in IscU. Biochem J 389: 797-802.
86. Ding H, Clark RJ, Ding B (2004) IscA Mediates Iron Delivery for Assembly of Iron-Sulfur Clusters in IscU under the Limited Accessible Free Iron Conditions. J Biol Chem 279: 37499-37504.

87. Layer G, Ollagnier-de Choudens S, Sanakis Y, Fontecave M (2006) Iron-Sulfur Cluster Biosynthesis: Characterization of Escherichia coli CyaY as an Iron Donor for the Assembly of [2Fe-2S] Clusters in the Scaffold IscU. J Biol Chem 281: 16256-16263.
88. Ding HG, Yang JJ, Coleman LC, Yeung S (2007) Distinct Iron Binding Property of Two Putative Iron Donors for the Iron-Sulfur Cluster Assembly: IscA and the bacterial frataxin ortholog CyaY under physiological and oxidative stress conditions. J Biol Chem 282: 7997-8004.
89. Adinolfi S, Iannuzzi C, Prischi F, Pastore C, Iametti S, et al. (2009) Bacterial frataxin CyaY is the gatekeeper of iron-sulfur cluster formation catalyzed by IscS. Nat Struct Mol Biol 16: 390-396.
90. Krebs C, Agar JN, Smith AD, Frazzon J, Dean DR, et al. (2001) IscA, an Alternate Scaffold for Fe-S Cluster Biosynthesis. Biochemistry 40: 14069-14080.
91. Yang J, Bitoun JP, Ding H (2006) Interplay of IscA and IscU in Biogenesis of Iron-Sulfur Clusters. J Biol Chem 281: 27956-27963.
92. Unciuleac MC, Chandramouli K, Naik S, Mayer S, Huynh BH, et al. (2007) In Vitro Activation of Apo-Aconitase Using a [4Fe-4S] Cluster-Loaded Form of the IscU [Fe-S] Cluster Scaffolding Protein. Biochemistry 46: 6812-6821.
93. Ding HG, Harrison K, Lu JX (2005) Thioredoxin Reductase System Mediates Iron Binding in IscA and Iron Delivery for the Iron-Sulfur Cluster Assembly in IscU. J Biol Chem 280: 30432-30437.
94. Bonomi F, Iametti S, Morleo A, Ta D, Vickery LE (2008) Studies on the Mechanism of Catalysis of Iron-Sulfur Cluster Transfer from IscU[2Fe2S] by HscA/HscB Chaperones. Biochemistry 47: 12795-12801.
95. Silberg JJ, Tapley TL, Hoff KG, Vickery LE (2004) Regulation of the HscA ATPase Reaction Cycle by the Co-chaperone HscB and the Iron-Sulfur Cluster Assembly Protein IscU. J Biol Chem 279: 53924-53931.
96. Bonomi F, Iametti S, Ta D, Vickery LE (2005) Multiple Turnover Transfer of [2Fe2S] Clusters by the Iron-Sulfur Cluster Assembly Scaffold Proteins IscU and IscA. J Biol Chem 280: 29513-29518.
97. Chandramouli K, Unciuleac MC, Naik S, Dean DR, Huynh BH, et al. (2007) Formation and Properties of [4Fe-4S] Clusters on the IscU Scaffold Protein. Biochemistry 46: 6804-6811.
98. Wink DA, Kasprzak KS, Maragos CM, Elespuru RK, Misra M, et al. (1991) DNA Deaminating Ability and Genotoxicity of Nitric Oxide and its Progenitors. Science 254: 1001-1003.
99. Dedon PC, Tannenbaum SR (2004) Reactive nitrogen species in the chemical biology of inflammation. Arch Biochem Biophys 423: 12-22.
100. Tamir S, Burney S, Tannenbaum SR (1996) DNA Damage by Nitric Oxide. Chem Res Toxicol 9: 821-827.
101. Burney S, Caulfield JL, Niles JC, Wishnok JS, Tannenbaum SR (1999) The chemistry of DNA damage from nitric oxide and peroxynitrite. Mutat Res 424: 37-49.
102. Richardson AR, Soliven KC, Castor ME, Barnes PD, Libby SJ, et al. (2009) The Base Excision Repair System of Salmonella enterica serovar Typhimurium Counteracts DNA Damage by Host Nitric Oxide. PLoS Pathog 5: e1000451.
103. Baute J, Depicker A (2008) Base Excision Repair and its Role in Maintaining Genome Stability. Crit Rev Biochem Mol Biol 43: 239-276.

104. Dong M, Wang C, Deen WM, Dedon PC (2003) Absence of 2'-Deoxyoxanosine and Presence of Abasic Sites in DNA Exposed to Nitric Oxide at Controlled Physiological Concentrations. Chem Res Toxicol 16: 1044-1055.
105. Berti PJ, McCann JAB (2006) Toward a Detailed Understanding of Base Excision Repair Enzymes: Transition State and Mechanistic Analyses of N-Glycoside Hydrolysis and N-Glycoside Transfer. Chem Rev 106: 506-555.
106. Kow YW (2002) Repair of deaminated bases in DNA. Free Radic Biol Med 33: 886-893.
107. Terato H, Masaoka A, Asagoshi K, Honsho A, Ohyama Y, et al. (2002) Novel repair activities of AlkA (3-methyladenine DNA glycosylase II) and endonuclease VIII for xanthine and oxanine, guanine lesions induced by nitric oxide and nitrous acid. Nucleic Acids Res 30: 4975-4984.
108. Zhao BY, O'Brien PJ (2011) Kinetic Mechanism for the Excision of Hypoxanthine by Escherichia coli AlkA and Evidence for Binding to DNA Ends. Biochemistry 50: 4350-4359.
109. Saparbaev M, Laval J (1994) Excision of hypoxanthine from DNA containing dIMP residues by the Escherichia coli, yeast, rat, and human alkylpurine DNA glycosylases. Proc Natl Acad Sci U S A 91: 5873-5877.
110. Demple B, Harrison L (1994) Repair of Oxidative Damage to DNA: Enzymology and Biology. Annu Rev Biochem 63: 915-948.
111. Garcin ED, Hosfield DJ, Desai SA, Haas BJ, Bjoras M, et al. (2008) DNA apurinic-apyrimidinic site binding and excision by endonuclease IV. Nat Struct Mol Biol 15: 515-522.
112. Faure V, Saparbaev M, Dumy P, Constant JF (2005) Action of multiple base excision repair enzymes on the 2'-deoxyribonolactone. Biochem Biophys Res Commun 328: 1188-1195.
113. McClure WR, Jovin TM (1975) The Steady State Kinetic Parameters and Non-Processivity of Escherichia coli Deoxyribonucleic Acid Polymerase I. J Biol Chem 250: 4073-4080.
114. Sung JS, Mosbaugh DW (2003) Escherichia coli Uracil- and Ethenocytosine-Initiated Base Excision DNA Repair: Rate-Limiting Step and Patch Size Distribution. Biochemistry 42: 4613-4625.
115. Lehman IR (1974) DNA Ligase: Structure, Mechanism, and Function. Science 186: 790-797.
116. Doetsch PW, Cunningham RP (1990) The enzymology of apurinic apyrimidinic endonucleases. Mutat Res 236: 173-201.
117. Wong I, Lundquist AJ, Bernards AS, Mosbaugh DW (2002) Presteady-state Analysis of a Single Catalytic Turnover by Escherichia coli Uracil-DNA Glycosylase Reveals a "Pinch-Pull-Push" Mechanism. J Biol Chem 277: 19424-19432.
118. Slupphaug G, Mol CD, Kavli B, Arvai AS, Krokan HE, et al. (1996) A nucleotide-flipping mechanism from the structure of human uracil-DNA glycosylase bound to DNA. Nature 384: 87-92.
119. Stivers JT, Pankiewicz KW, Watanabe KA (1999) Kinetic Mechanism of Damage Site Recognition and Uracil Flipping by Escherichia coli Uracil DNA Glycosylase. Biochemistry 38: 952-963.
120. Fedorova OS, Kuznetsov NA, Koval VV, Knorre DG (2010) Conformational Dynamics and Pre-Steady-State Kinetics of DNA Glycosylases. Biochemistry (Mosc) 75: 1225-1239.

121. Bertram JG, Oertell K, Petruska J, Goodman MF (2010) DNA Polymerase Fidelity: Comparing Direct Competition of Right and Wrong dNTP Substrates with Steady State and Pre-Steady State Kinetics. Biochemistry 49: 20-28.
122. Schmidt K, Desch W, Klatt P, Kukovetz WR, Mayer B (1997) Release of nitric oxide from donors with known half-life: a mathematical model for calculating nitric oxide concentrations in aerobic solutions. Naunyn Schmiedebergs Arch Pharmacol 355: 457-462.
123. Justino MC, Vicente JB, Teixeira M, Saraiva LM (2005) New genes implicated in the protection of anaerobically grown Escherichia coli against nitric oxide. J Biol Chem 280: 2636-2643.
124. Kotte O, Zaugg JB, Heinemann M (2010) Bacterial adaptation through distributed sensing of metabolic fluxes. Mol Syst Biol 6: 355.
125. Hyduke DR, Jarboe LR, Tran LM, Chou KJ, Liao JC (2007) Integrated network analysis identifies nitric oxide response networks and dihydroxyacid dehydratase as a crucial target in Escherichia coli. Proc Natl Acad Sci U S A 104: 8484-8489.
126. Cruz-Ramos H, Crack J, Wu GG, Hughes MN, Scott C, et al. (2002) NO sensing by FNR: regulation of the Escherichia coli NO-detoxifying flavohaemoglobin, Hmp. EMBO J 21: 3235-3244.
127. Hutchings MI, Mandhana N, Spiro S (2002) The NorR Protein of Escherichia coli Activates Expression of the Flavorubredoxin Gene norV in Response to Reactive Nitrogen Species. J Bacteriol 184: 4640-4643.
128. Vanin AF, Serezhenkov VA, Mikoyan VD, Genkin MV (1998) The 2.03 Signal as an Indicator of Dinitrosyl-Iron Complexes with Thiol-Containing Ligands. Nitric Oxide-Biol Ch 2: 224-234.
